# Tumor Suppressor Function of Syk in Human MCF10A *In Vitro* and Normal Mouse Mammary Epithelium *In Vivo*

**DOI:** 10.1371/journal.pone.0007445

**Published:** 2009-10-15

**Authors:** You Me Sung, Xuehua Xu, Junfeng Sun, Duane Mueller, Kinza Sentissi, Peter Johnson, Elana Urbach, Françoise Seillier-Moiseiwitsch, Michael D. Johnson, Susette C. Mueller

**Affiliations:** 1 Department of Oncology, Lombardi Comprehensive Cancer Center, Georgetown University Medical Center, Washington, D. C., United States of America; 2 Department of Biostatistics, Bioinformatics and Biomathematics, Lombardi Comprehensive Cancer Center, Georgetown University Medical Center, Washington, D. C., United States of America; Health Canada, Canada

## Abstract

The normal function of Syk in epithelium of the developing or adult breast is not known, however, Syk suppresses tumor growth, invasion, and metastasis in breast cancer cells. Here, we demonstrate that in the mouse mammary gland, loss of one Syk allele profoundly increases proliferation and ductal branching and invasion of epithelial cells through the mammary fat pad during puberty. Mammary carcinomas develop by one year. Syk also suppresses proliferation and invasion *in vitro*. siRNA or shRNA knockdown of Syk in MCF10A breast epithelial cells dramatically increased proliferation, anchorage independent growth, cellular motility, and invasion, with formation of functional, extracellular matrix-degrading invadopodia. Morphological and gene microarray analysis following Syk knockdown revealed a loss of luminal and differentiated epithelial features with epithelial to mesenchymal transition and a gain in invadopodial cell surface markers CD44, CD49F, and MMP14. These results support the role of Syk in limiting proliferation and invasion of epithelial cells during normal morphogenesis, and emphasize the critical role of Syk as a tumor suppressor for breast cancer. The question of breast cancer risk following systemic anti-Syk therapy is raised since only partial loss of Syk was sufficient to induce mammary carcinomas.

## Introduction

Understanding the normal function of Syk in the developing and adult breast, its upstream activators and downstream effectors, and epigenetic and genetic factors that cause its loss are vital for breast cancer prognosis, prediction and treatment. However, Syk plays an integral role in immune cell development and activation and it has been identified as a drug target for autoimmune disease, allergy and asthma. Syk is a tumor suppressor in breast cancer and may well also be in gastric cancer and melanoma, although it may be a tumor promoter in some invasive head and neck cancers and certain lymphomas [for review, [1] and [2], [3]]. A complete understanding of the role of Syk as a tumor suppressor is vital for assessing breast cancer risk in patients who might be candidates for therapeutic suppression of Syk to control immune-related disease.

Syk mRNA is increasingly lost from normal adjacent to hyperplastic to ductal carcinoma *in situ* (DCIS) to invasive tumors compared with normal, disease free breast epithelium [4]. The gene is hypermethylated in invasive breast cancer and loss of Syk has been tied to poor outcome in breast cancer [for review, [1]]. Functional studies in nude mice and *in vitro* illustrate the role of Syk in suppressing motility, invasion, and metastasis [1], [5]. Syk has been shown to block breast cancer cell migration and chemoinvasion via mechanisms involving down regulation of secreted growth factor GRO-1 [6] and suppression of NF-κB, PI3K [7], Src [4], and EGFR signaling pathways [8] [see also for review, [1]].

Studies on the role of Syk in cancer to date have all been conducted using tumor cell lines with reintroduction of Syk into highly invasive Syk negative cell lines or interference with endogenous Syk in otherwise non-invasive tumor cells. However, the normal function of Syk in mammary epithelium has not been addressed, nor have experiments been performed to determine the consequences of Syk loss from normal epithelial cells. MCF10A immortalized breast epithelial cells and cells derived from them serve as an *in vitro* model system to understand normal epithelial function and development [9], [10]. MCF10A, MCF10AneoT, and DCIS.com form a series with which to model early cancer progression [11]. MCF10A are Syk positive, but the Syk status or functionality in this progression series is not known. The mouse mammary gland is another system to model normal epithelial behavior. The branching network of epithelial ducts arises from the combination of proliferation and epithelial ductal invasion through the fat pad via the formation of terminal end buds (TEB), TEB bifurcation and lateral branching, and ductal elongation. Unfortunately, nothing is known concerning Syk function in the adult or developing breast. Syk knockout mice have been described [12], [13]. However, homozygous deletion of Syk is perinatal lethal, due to failure of lymphatic and blood vessels to properly separate [14]. Other aspects of development such as formation of mammary gland were not reported. Here we determine the role of Syk in normal epithelium using these *in vitro* and *in vivo* models. Taken together our results are consistent with a critical role for Syk in blocking pre-neoplastic cell proliferation and invasion and suggest that Syk does so by affecting several major signaling pathways important for normal breast development and maintenance.

## Results

### *In vitro* studies

#### Knockdown of Syk induces increased migration and cell proliferation in human breast epithelial cells

**Figure 1:**
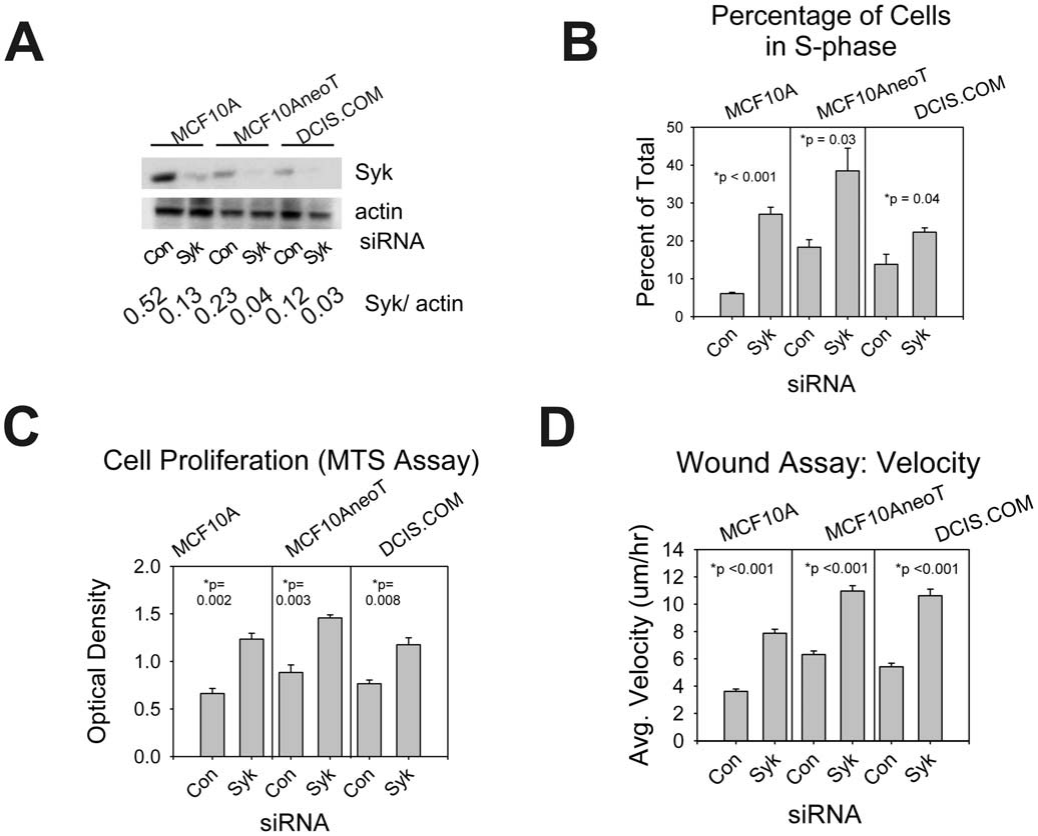
Cell proliferation and migration of MCF10A, MCF10AneoT, and DCIS.com human mammary epithelial cells. (A) Decreases in Syk protein levels are shown for control versus Syk siRNA transfection of normal MCF10A cells and tumorigenic MCF10AneoT and DCIS.com cells. Increased cell proliferation (B) and cell growth (C) was observed following Syk knockdown. (D) Increased velocity of MCF10A, MCF10AneoT, and DCIS.com cell migration was observed in Syk knockdown cells using a scratch/wound assay. (B–D) Data is represented as means +/− SEM. P values are significant by the student's t-test for all cell lines, average of 3 independent experiments.

First, we investigated whether Syk loss in normal human breast epithelial cells would have an impact on the ability of these cells to proliferate and invade. For these studies, we utilized non-transformed, immortalized MCF10A human breast epithelial cells (MCF10A1) and two cell lines derived from them, MCF10AneoT, and MCF10DCIS.com (DCIS.com), with characteristics of normal, hyperplastic, and ductal carcinoma in situ (DCIS), respectively. MCF10A contained the greatest amount of Syk and MCF10AneoT and DCIS.com each had progressively less (Figure 1A). Syk siRNA treatment effectively lowered Syk protein levels up to 90% in all three cell lines (Figure 1A). Analysis of cell proliferation and growth revealed that loss of Syk in each cell line led to enhanced cell proliferation, approximately 4-fold greater in MCF10A cells, 2 fold greater in MCF10AneoT, and 0.4 greater in DCIS.com (Figures 1B and 1C).

Next, we asked whether normal cells or the transformed MCF10A derivatives migrated more efficiently following Syk knockdown using a scratch/wound assay. Cell velocities and rate of gap closure of the monolayer were significantly increased in each cell line following Syk siRNA knockdown (Figures 1D and S1). Thus, loss of Syk enhances migration in normal breast epithelial cells as well as in tumor-forming cell lines.

#### Effects of Syk knockdown on invasion and branching in 3D matrices

**Figure 2:**
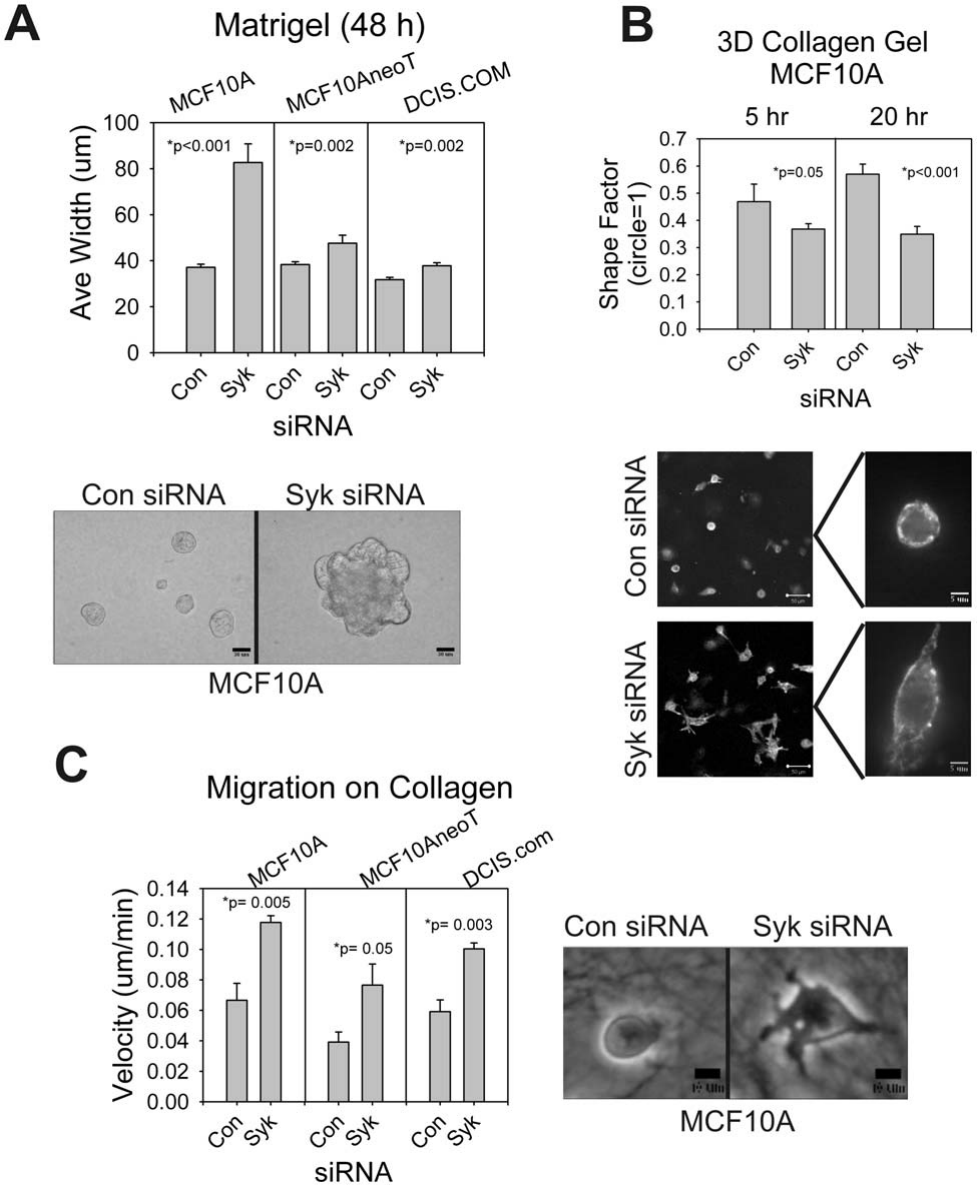
siRNA knockdown of Syk increases motility and invasive cell morphology in three dimensional cultures. (A) Cells were transfected with control or Syk siRNA and cultured in Matrigel for 48 hours. MCF10A colony size was the most dramatically influenced by Syk knockdown. Scale bars correspond to 200 µm. (B) Cells were seeded at sub-confluent densities on top of bovine collagen I gels (3.0 mg/ml), cultured 5 or 20 hours, fixed and stained with AlexaFluor488-phalloidin. Images of cells cultured for 20 h are shown. Scale bars are 50 µm (at left) and 5 µm in the higher magnification views at right. The invasive phenotype was quantified by measuring shape factor. A shape factor of 1 is equivalent to a circle. (C) In a separate experiment, the velocity of cell migration on collagen I was determined. Scale bars are 10 µm. (A–C) P values are significant by the student's t-test for all three cell lines. Data are represented as mean +/− SEM.

To determine whether loss of Syk in normal breast epithelial cells would lead to acquisition of an invasive phenotype in 3D Matrigel, we cultured cells in Matrigel following siRNA knockdown. After 48 hours of culture, MCF10A cells treated with control siRNA formed small round cell aggregates whereas Syk siRNA dramatically increased the size of the colonies which took on an irregular shape similar to initial stages of *in vitro* branching (Figure 2A). The effect was not as dramatic in the MCF10AneoT and DCIS.com cells, but in all three cell lines, the differences were statistically significant (Figure 2A).

Similarly, we determined the morphology of the epithelial cells in 3.0 mg/ml bovine collagen I gels, a setting that is more closely akin to the stromal cell environment. Following Syk knockdown, MCF10A cells took on a more fibroblast-like morphology in collagen, extending more cellular protrusions (Figure 2B). Cells became significantly less round in shape as shown in the representative phalloidin-stained cells and as quantified by cell shape (Figure 2B). Cell migration on the collagen I gels was significantly enhanced by siRNA targeting Syk compared with control siRNA (Figure 2C). siRNA knockdown experiments, in summary, have demonstrated that loss of Syk significantly enhances cell proliferation, migration, and invasive morphologies in normal and transformed breast epithelial cells alike, and the changes in migration and invasion can be observed in assays on plastic, or in 3D matrix settings such as Matrigel or collagen I mats.

#### Loss of Syk increases tumorigenic and metastatic potential

**Figure 3:**
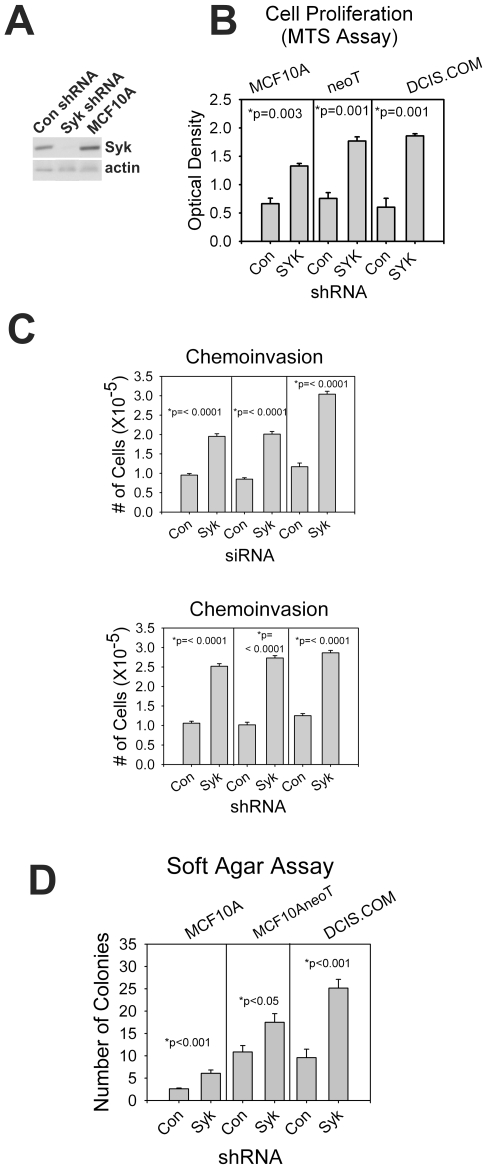
siRNA and shRNA knockdown of Syk enhances chemoinvasion and anchorage independent proliferation. (A) MCF10A, MCF10AneoT, and DCIS.com cells were transduced with Syk or control shRNA in a lentiviral vector. Western blot analysis of puromycin-selected cell populations is shown. (B) The MTS proliferation assay revealed that shRNA cell lines behave similarly to the siRNA transiently transfected cells with respect to Syk knockdown; cell proliferation was enhanced. (C) Chemoinvasion assays were performed in parallel using cells transiently transfected with control or Syk siRNA or stably transduced with lentiviral control or Syk shRNA. Loss of Syk increases chemoinvasion in every case. (D). In soft agar assays, each of the Syk shRNA knockdown cell lines experienced increased colony formation. Representative images are shown in Figure S3. (B–D) Data are represented as means +/− SEM. P values are significant by the student's t-test for each cell line.

Soft agar assay and chemoinvasion assays through Matrigel in Boydon chambers are more accurate predictors of increased metastatic potential. Therefore, we created shRNA knockdown cell lines for MCF10A, MCF10AneoT, and DCIS.com to obtain stably transfected cells for use in longer term experiments (Figure 3A). Similar to siRNA knockdown of Syk, shRNA knockdown enhanced cell proliferation by 2–3 fold in each of the cell lines measured by the MTS assay (Figure 3B). In Boydon chamber chemoinvasion assays, both siRNA and shRNA Syk were effective in substantially increasing chemoinvasion (Figure 3C). Similarly, loss of Syk in shRNA stably-expressing cell lines was associated with significantly enhanced colony growth within soft agar (Figures 3D and S2). We conclude from this series of experiments, that Syk loss results in increased chemoinvasion and enhanced colony formation in normal and transformed human mammary epithelial cells.

#### Syk knockdown gene expression changes in MCF10A cells related to epithelial-to-mesenchymal transition (EMT), growth factor and stress pathways, and invadopodia formation

**Figure 4:**
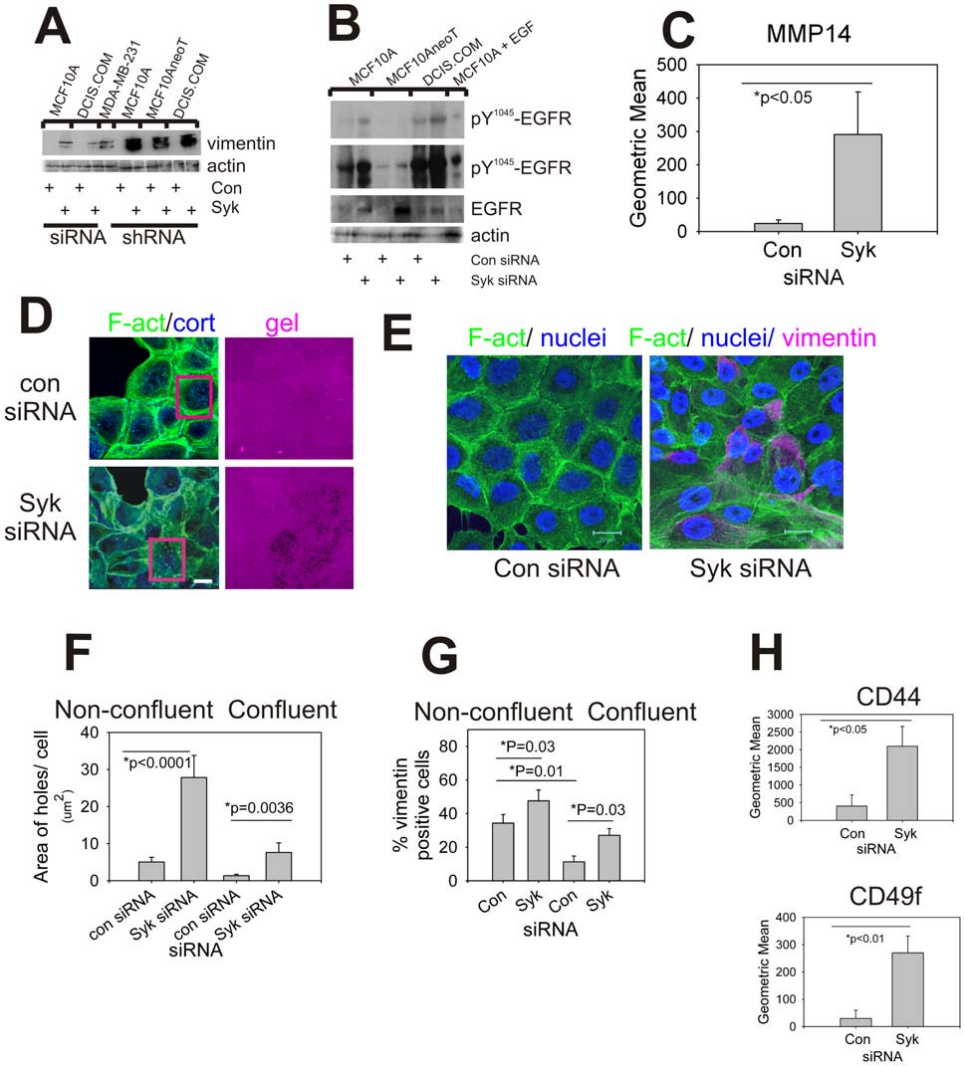
Syk knockdown MCF10A cells express EMT marker, invadopodia, and stem/progenitor marker proteins, and invadopodia. (A) Cell extracts from cells cultured on plastic were blotted with anti-vimentin antibodies and then re-probed with anti-α-actin antibodies. Vimentin protein was up-regulated following Syk knockdown. (B) Cell extracts were also probed for anti-pY^1045^-EGFR, and then re-probed for anti-EGFR and anti-α-actin. Overall, activated EGFR was up-regulated by Syk knockdown in MCF10 and DCIS.com, but not markedly in MCF10AneoT. The top most blot is a shorter exposure than the one below it. Results for shRNA are shown in Figure S5A. (C) Flow cytometry was used to measure cell surface MMP14 staining of control versus Syk siRNA knockdown in MCF10A. Three experiments were averaged. BT549 breast cancer cells were used as positive control for cell surface MT1-MMP (mean 420.8). (D) Images from the gelatin-degradation assay for invadopodia from MCF10A cells from three color confocal imaging (phalloidin, green; cortactin, blue; gelatin, magenta). Holes are formed in the gelatin matrix (gel) following Syk siRNA knockdown. Scale bars  = 20 µm. A higher magnification view of the area within the red boxes is shown in Figure S5B. (E) Three color confocal images were taken of cells cultured on crosslinked gelatin showing the distribution of DAPI (nuclei, blue), phalloidin (F-act, green), and vimentin (magenta) in control versus Syk siRNA transfected MCF10A cells. Scale bars  = 20 µm. (F) Invadopodia activity was upregulated in Syk siRNA transfected MCF10A. (G) Number of vimentin positive cells was increased in Syk siRNA transfected MCF10A cells in non-confluent and confluent cultures. (H) Results for two color flow cytometry from three experiments each for cell surface CD44/CD24 and CD49f/CD24 demonstrate significant elevation in CD44 and CD49f at the cell surface following Syk knockdown by siRNA in MCF10A cells.

To explore the mechanisms whereby loss of Syk promotes invasion, microarray analysis was performed on MCF10A cells transiently transfected with control or Syk siRNA and cultured on a mat of collagen I. After Syk knockdown and culture on collagen, the phenotype of cells does become less epithelial and more mesenchymal (Figure 2) as indicated by loss of keratin genes (Keratin 6B, 10, 23), genes associated with stratified epithelium (small proline-rich protein 1A, involucrin, sciellin) and luminal mammary epithelium (CD24), the extracellular matrix (CD36, the thrombospondin receptor) and gain of genes related to integrin/ECM signaling (integrin beta 5, integrin-linked kinase, laminin gamma 1), collagen biosynthesis and crosslinking (lysyl oxidase (LOX), lysyl oxidase-like 2 (ENTPD4), procollagen-lysine, 2-oxoglutarate 5-dioxygenase 2 (PLOD2), procollagen-proline, 2-oxoglutarate 4-dioxygenase (proline 4-hydroxylase), alpha polypeptide I (P4HA1)), and growth factor signaling (EGFR, VEGF, TGFβ1) (Table S1). This suggests that Syk loss releases inhibition of signaling pathways that are normally repressed in epithelial cells and that promote increased proliferation, motility, and invasiveness. Consistent with the changes in phenotype that we observed, i.e., following epithelial-to-mesenchymal transition (EMT), we found that vimentin protein expression was dramatically increased following Syk siRNA knockdown in MCF10A, MCF10AneoT, and DCIS.COM (Figure 4A).

To assess overall gene expression changes due to Syk loss in more detail, we performed an Ingenuity Pathway analysis using as input all of the genes that were differentially expressed on collagen following Syk knockdown. The analysis resulted in the identification of a number of networks and their interactions (Table S2). Several of these networks were linked and identified differentially expressed genes related to EMT and Syk signaling including EGFR, TGFβ1, and NFkB networks (Figure S3 and Table S2). These interactive networks are summarized in Figure S4 with “hubs” that include EGFR, TGFβ1, NFkB, integrin, collagen, and Rac. Therefore, microarray analysis supports the role of these pathways and their contribution to the changes in phenotype and behavior induced following Syk loss.

#### Invadopodia-related gene expression

Using the same microarray data, we asked whether expression of invadopodia-related genes was enhanced in MCF10A Syk knockdown cells cultured on collagen. We prepared a list of proteins (total 63, 117 probe sets) required for localized matrix degradation, a functional identifier of the presence of invadopodia [15] and determined that a subset were differentially expressed following Syk knockdown and culture on collagen. CORO1A, WASL, GRB2, and PLAUR were down regulated and ITGB1, PAK1, MAPK7, LAMP1, WASF1, VEGFA, ADAM12, and TGFβ1 were up regulated (Table S3). The multilayered relationships connecting the gene networks differentially regulated by Syk identified in Figures S1 and S2 and the invadopodia gene network (data not shown) are shown in Figure S1 (blue lines).

Since the epidermal growth factor receptor (EGFR) mRNA was up-regulated (Table S1), but did not appear on the invadopodia network (not shown), we validated up regulation of EGFR at the protein level. We found that EGFR levels increased markedly in the MCF10AneoT (ras-transformed) cells (Figures 4B and S5A). Phosphorylation of Y1045 EGFR was up regulated slightly (less than 2 fold) in both Syk siRNA and Syk shRNA knockdown cells on collagen in each of the cell lines (Figures 4B and S5A).

#### Functional invadopodia

To determine whether invadopodia formation is linked to EMT and increased cellular invasion, we asked whether invadopodia were induced or increased following Syk loss. Since MMP14 is required for invadopodia-mediated matrix degradation and invasion on both collagen and gelatin [16], [17], we first measured MMP 14 (MT1-MMP) using flow cytometry (FACS). Cell surface expression was dramatically increased following Syk knockdown in cells cultured on plastic (Figure 4C).

Next, we performed a localized degradation assay to measure invadopodia activity by culturing cells on fluorescent, crosslinked films [18]. Evidence that cells underwent EMT was further obtained by immuno-staining cell monolayers with phalloidin to detect F-actin and cortactin (Figure 4D) in cells seeded on fluorescent crosslinked gelatin to detect invadopodia, or F-actin, cortactin, and vimentin (Figure 4E). We found in cells seeded at confluent density that control siRNA-treated cells were cobblestone in appearance and polarized and the cell-cell borders were delineated by adjacent rings of actin filaments (Figures 4D, E, and S5B, con siRNA). In contrast, the Syk siRNA-treated cells were disorganized in appearance with regions of fibroblast-like cell spreading (Figures 4D, E and S5B, Syk siRNA).

The formation of invadopodia-induced holes in the fluorescent matrix was quantified. Syk siRNA knockdown followed by plating at non-confluent (40–45%) conditions elevated hole formation 5.6 fold over the control siRNA-treated cells, whereas at confluent (90–95%) conditions, Syk siRNA knockdown elevated hole formation 5.9 fold over the control cells (Figures 4D and F). Thus, loss of Syk increased invadopodia-mediated matrix degradation.

Invadopodia formation and vimentin expression are correlated with Syk negativity in breast cancer cells [19]–[21]. To determine the relationship between vimentin expression and the ability to form invadopodia, we stained cells with anti-vimentin antibody, phalloidin, and DAPI (Figure 4E). 34% of cells cultured at sub-confluent density on gelatin films contained vimentin-positive filaments and the percentage of vimentin-positive cells increased to 48% after Syk knockdown (Figure 4G, Non-confluent). The percentage of vimentin-positive cells increased from 11% to 27% after Syk knockdown in confluent cells (Figure 4G, Confluent). Examining individual cells and the subtending matrix, we determined that there was no correlation between cells that expressed vimentin filaments and those that formed invadopodia since 43% of Syk knockdown cells forming holes were vimentin filament-positive and the remaining cells were vimentin filament-negative.

#### Overlap of invadopodia and breast cancer stem cell markers

Participation of CD44 (hyaluronan receptor) and CD49f (α6 integrin) in invadopodia (tumor cells) or podosomes (immune cells) has been previously reported [22]–[24]. We asked whether the cell surface expression of CD44 or CD49f was altered in MCF10A following Syk knockdown in concert with CD24 expression changes. CD24 is a luminal epithelial marker, and is used as a breast cancer stem cell marker in combination with other stem/progenitor cell markers such as CD49f (integrin alpha6) and CD44 [25]–[27]. FACS co-staining analysis of CD44/CD24 and CD49f/CD24 revealed that CD44 and CD49f were dramatically enhanced at the cell surface, whereas CD24 was modestly suppressed or unaffected (Figures 4H and S5C).

### *In vivo* studies

#### Syk loss increases ductal branching and proliferation in the Syk ^+/−^ mouse mammary gland

To determine the effect of Syk loss during normal mammary gland development *in vivo*, we examined Syk hetero-knockout (Syk ^+/−^) mice (129 background) [12]. Homozygous Syk knockout mice are not viable, with most mice dying at approximately 16.5 days of gestation [12]. At 10 and 12 weeks of age, mammary glands from virgin Syk ^+/−^ female mice showed increased ductal branches and alveolar development compared with Syk ^+/+^ mice in the 4rth inguinal gland as shown in the whole mount analysis (Figures S6A–C). As expected, Syk protein expression was strongly decreased in mammary gland and spleen from virgin Syk ^+/−^ female mice compared with those tissues of wild type (WT) female mice (129 back ground) (Figure S6D). These data suggest that Syk loss might enhance ductal development in mammary gland.

#### Syk ^+/−^ (FVB) mammary glands show strongly enhanced branching morphogenesis

**Figure 5:**
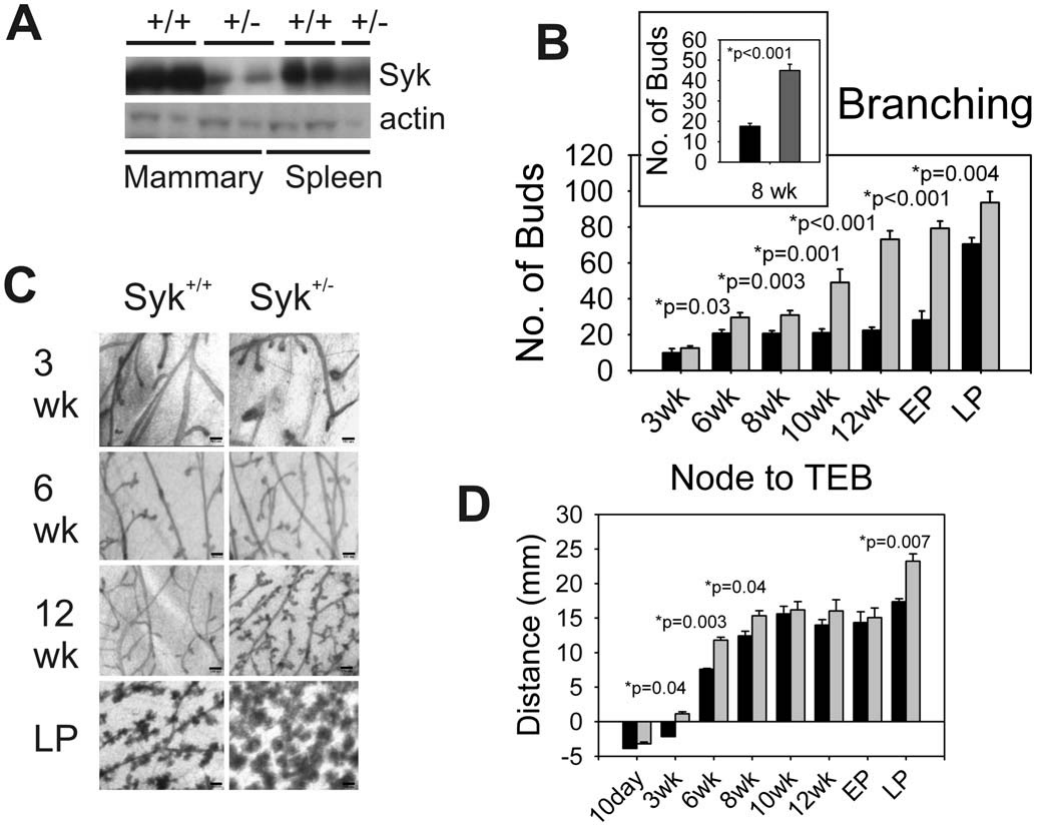
Enhanced branching morphogenesis in mammary glands of Syk ^+/−^ mice. (A) Syk protein is decreased in mammary gland extracts (minus the mammary gland lymph node) and spleen from 12-week virgin Syk ^+/−^ heterozygous compared with Syk ^+/+^ wild type females. Spleen extracts were used as positive control. (B) The average number of buds were counted in mammary glands from Syk ^+/+^ wild type (black bars) and Syk ^+/−^ heterozygous mice (gray bars) from 3-, 6-, 8-, 10-, 12-week virgin and early and late pregnancy females (EP and LP, respectively). P value was significant by the student's t-test for 6-, 8-, 10-, 12-week virgin females and early- and late-pregnancy females (n = 3 animals per time point). In a separate experiment, mammary glands from 10 additional wild type and 10 heterozygous knockout Syk mice were examined at 8 weeks confirming the increase in bud formation after partial Syk loss (B, inset). Data are represented as means from three mice +/− SEM except for B, inset, where n = 10 mice. (C) Representative images of mammary glands from Syk ^+/+^ wild type and Syk ^+/−^ heterozygote knockout mice. Scale bars correspond to 150 µm in all panels. (D) The mean distance was determined from lymph node to the distal end of ducts in mammary glands from Syk ^+/+^ wild type (black bars) and Syk ^+/−^ heterozygous mice (gray bars) at 10-day and 3-,6-,8-,10, 12-week virgin and early and late pregnancy females (n = 3 animals per time point). P value is significant by the student's t-test for 3-, 6-, 8-week virgin females and late-pregnancy females. Data are represented as means +/− SEM for three glands each.

The Syk ^+/−^ mice (129 background) were crossed with eGFP ^+/+^ mice (FVB background) and loss of Syk expression in heterozygote female mammary glands was confirmed (Figure 5A). Using immunohistochemistry, Syk expression was strongly detected in mammary glands of WT female mice where it localized to luminal epithelium and mammary gland lymph nodes in subsets of lymphocytes (Figure S5A, Syk ^+/+^) and in the spleen (not shown). At those same sites, Syk was decreased in Syk ^+/−^ mice (Figure S7A, Syk ^+/−^).

To examine the morphology of ductal development during puberty, whole mount analysis was performed on Syk ^+/−^ and WT female mammary glands at different ages. In strong agreement with the evident change in morphology of Syk ^+/−^ mice on a 129 background, mammary glands from virgin Syk ^+/−^ female mice displayed more abundant ductal branches and end buds compared with the wild type glands as shown in the whole mount analysis at 3, 6, 8, 10, 12 weeks and pregnancy (EP, early pregnancy, E6.5 and LP, late pregnancy, E16.5) (Figures 5B and 5C). Next, we found that the distance from lymph node to TEB in the mammary gland was significantly increased in the Syk ^+/−^ mice compared to that of WT mice during pre-puberty (3 weeks), puberty (6 and 8 weeks) as well as in late pregnancy glands (Figure 5D). The differences were not significant at other time points.

We next analyzed mammary gland morphology of pubertal and multiparous mice to determine if Syk loss affects end bud morphology or results in epithelial hyperplasia and abnormal ducts or end buds. In 10 week animals, the branches and end buds of Syk ^+/−^ mice formed lumens similar to the wild type animals, although in the heterozygote mammary glands, many more terminal ends formed along the length and tips of the ducts (Figure S7B, X-Y, 10 wk). Similarly, glands from late pregnancy (16.5) Syk ^+/−^ mice had many more lobules (Figure S7B, X-Y, LP). The lobules, like those from the wild type pregnant females, were hollow balls, however, the size and density of formation was significantly enhanced compared with wild type (Figure S7B, X-Y view, LP).

We conclude that the effect of heterozygotic Syk loss in mammary gland development is present in two different strains of mice. Taken together, these data indicate that Syk is haploinsufficient and reduced levels can significantly impact murine mammary gland development.

#### Increased proliferation and invasiveness of mouse mammary epithelial cells *in vivo* and *in vitro*

**Figure 6:**
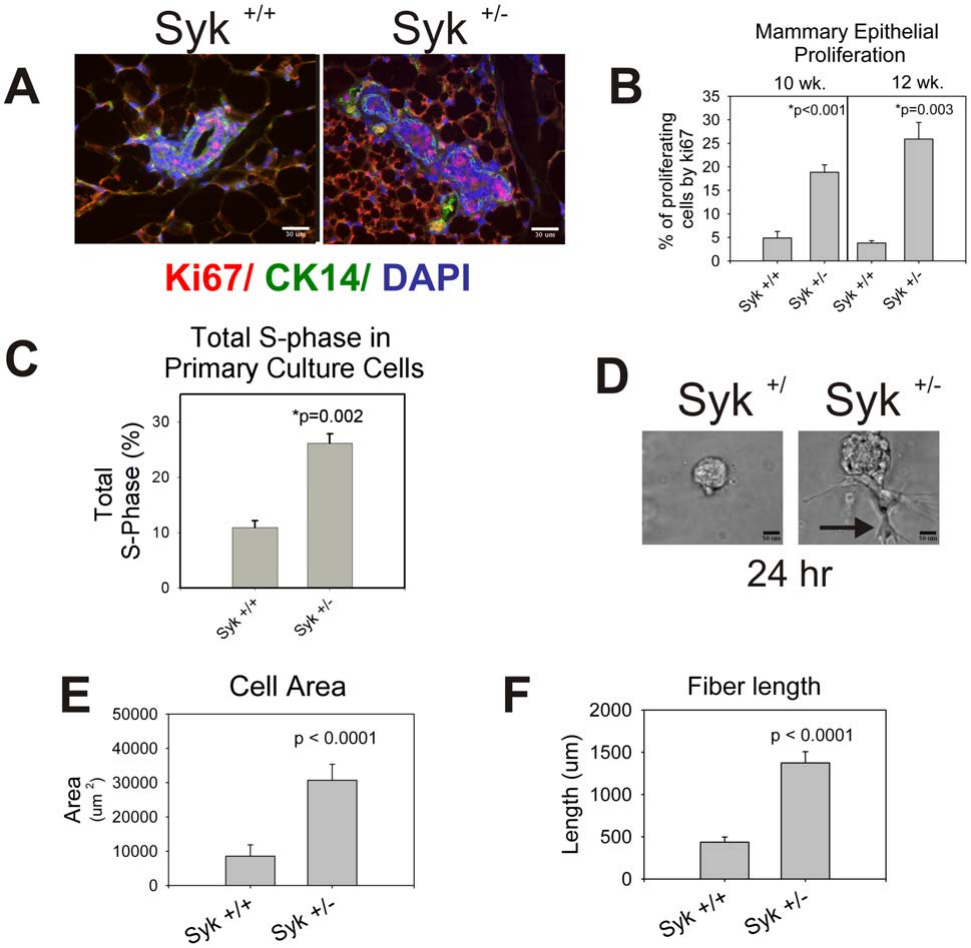
Heterozygous Syk knockout accelerates cell proliferation and invasiveness of mouse mammary epithelial cells. (A) Representative images illustrate sections triple stained with anti-Ki67 (red), anti-keratin14 (CK14m green), and DAPI (blue) from mammary glands of Syk ^+/+^ wild type and Syk ^+/−^ heterozygous mice from 12-week virgin females. Scale bars correspond to 30 µm. (B) Increased mammary epithelial cell proliferation was quantified from images illustrated in (A) in three animals for each point. (C) Primary cell cultures also exhibit increased cell proliferation. Total % of cells in S-phase was quantified in primary cells from Syk ^+/+^ wild type and Syk ^+/−^ heterozygous mice from 12-week virgin females. (D) Freshly isolated primary mammary epithelial cells were embedded Matrigel. Organoids were imaged at 24 hrs and show greatly enhanced size in Syk heterozygous knockdown compared with wild type glands. A typical invasive leading edge is indicated (arrow). Scale bars correspond to 50 µm. Increased cellular area (E) and fiber length (F) of organoids was assessed using Metamorph Image software. In all cases (B–F), data is represented by means +/− SEM. P values are significant by the student's t-test.

Following the observation of increased ductal development in Syk ^+/−^ mice, we performed an assessment of cell proliferation in mammary gland tissues to determine whether epithelial cells were more proliferative after Syk loss. In both 10 and 12 week animals, the % positive Ki67 nuclei were significantly and dramatically increased in the heterozygote knockout Syk ^+/−^ virgin mice compared with the wild type (Figures 6A and B).

Next, we isolated primary mouse epithelial cells to determine *in vitro* their proliferative and invasive capacity. The percent of primary epithelial cells isolated from mammary glands of the Syk ^+/−^ virgin females in S-phase was 2.4 fold higher than wild type animals (Figure 6C). We used a three-dimensional *in vitro* model consisting of primary mammary epithelial cells embedded in a reconstituted basement membrane matrix (Matrigel) to assess growth and invasion. Epithelial cells extracted from mammary gland(s) of wild type mice formed spherical ‘cysts’ without branches during the course of 24 hour culture (Figure 6D). In contrast, cells isolated from Syk ^+/−^ mice formed invasive, branching processes after 24 h (Figures 6D, arrow and 6E, F). Thus, both proliferation and invasion were enhanced in primary mouse epithelial cells in heterozygote glands compared with wild type.

#### Mammary hyperplasia and tumor formation in heterozygote animals

**Figure 7:**
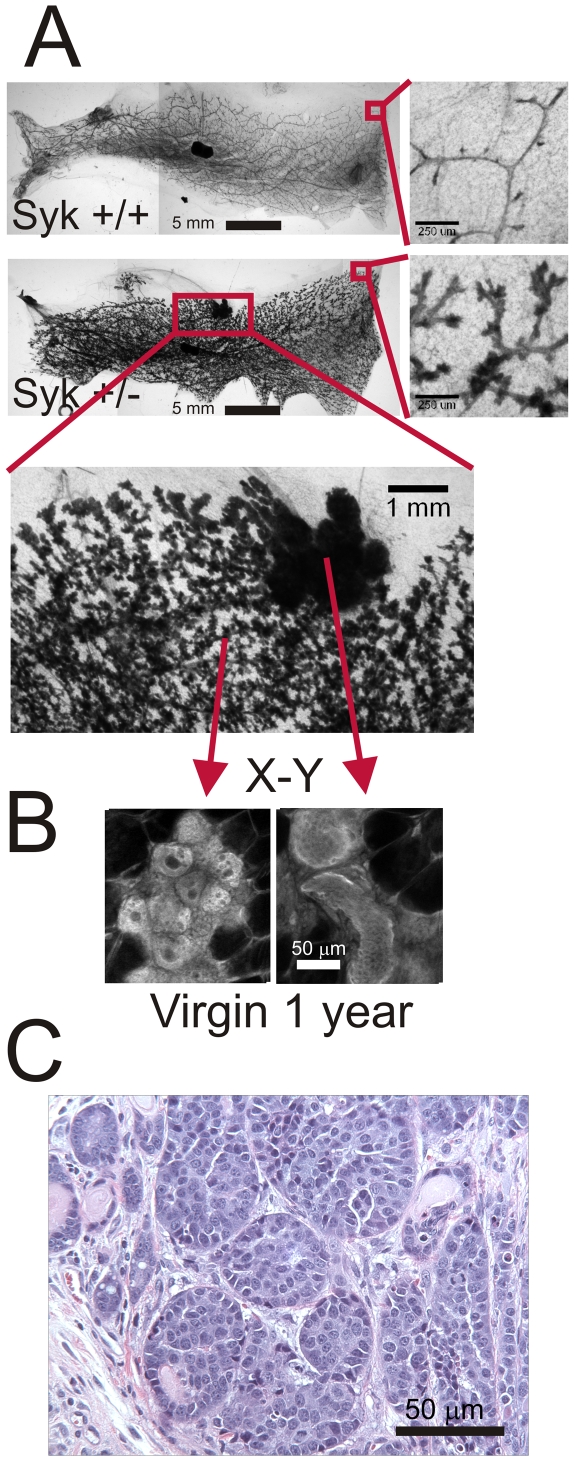
Heterozygous Syk ^+/−^ mice develop mammary gland hyperplasia and ductal carcinoma. (A) At one year in virgin females, 4^th^ inguinal mammary glands reveal a denser epithelial network in heterozygote compared to wild type glands. Insets at right are higher magnification views of the distal ductal tree. An area of hyperplasia in the heterozygote gland (Syk +/−) indicated by a red box is enlarged. Scale bars correspond to 5 mm and 250 µm insets at right and 1 mm inset below. (B) Multiphoton imaging of carmine red autofluorescence in whole mounts of the Syk +/− gland revealed that the branching epithelium is over developed but lumens are visible (X-Y, inset at left). The hyperplastic portion contains no lumens (X-Y, inset at right). Scale bars correspond to 50 µm. (C) Hemotoxylin/eosin stained paraffin section from an invasive ductal carcinoma taken from a one year, virgin female Syk ^+/−^. Scale bar is 50 µm.

In order to determine whether aged heterozygote animals develop tumors, five virgin Syk ^+/−^ mice and virgin wild type mice were examined at one year. Whole mounts of the fourth inguinal wild type mammary glands revealed limited ductal structure in wild type glands, whereas those of the Syk ^+/−^ animals were densely filled with epithelial structure (Figure 7A). Loci of hyperplasias were found in the mammary gland whole mounts of the Syk ^+/−^ animals (Figures 7A and B). The epithelial structures were clusters of lobules each containing a small central lumen, whereas the hyperplasias contained densely packed solid cores of irregularly shaped lobules (Figure 7B, X-Y views).

At one year, each of the five Syk ^+/−^ females had formed mammary tumors associated with the 3^rd^ or 4^th^ inguinal glands whereas none of the Syk ^+/+^ wild type females had formed them. The tumors, one in each animal, were attached to the mammary fat pad (not shown). The tumors were invasive, intraductal carcinoma (IDC). A representative view of one of the tumors is shown in Figure 7C. At one year, at least one animal has shown the development of lung metastases.

## Discussion

The progression cell line series of MCF10A, MCF10AneoT, and DCIS.com exhibited a progressive loss of Syk. This result mirrors the loss of Syk observed in breast tissues from patients [4]. Strikingly, knockdown of Syk using either siRNA or shRNA consistently enhanced proliferation, motility, invasion, and anchorage-independent growth in each of these cell lines along the progression series to DCIS.com, a cell line modeling ductal carcinoma in situ (DCIS). Thus, changes in levels of Syk potently mediate their effects on these activities and suggest that Syk must be present in epithelial cells at all stages of progression including benign tissues in order to suppress invasive, tumor-like cell behavior. The results in human breast epithelial cells are supported by the results in the mouse mammary gland where loss of one allele had an impressive effect on mammary ductal structure and ultimately on carcinoma development. In both human and mouse epithelial cells, we demonstrated increased cellular proliferation and invasion. The mouse heterozygote model further demonstrates that partial systemic loss of Syk can result in hyperplastic growth, tumor formation, and in some cases metastasis. The results on Syk knockdown in MCF10A cells support the interpretation that Syk is required in the epithelial cells of normal mouse mammary glands to control proper growth and invasion during puberty although it is possible that additional factors contribute since Syk is present in other tissues including immune cells and endothelium [1].

Multiple lines of evidence suggest that loss of Syk triggers EMT. EMT-related phenotypic changes are evidenced by change in cellular morphology and size in Matrigel and collagen I matrix. In addition, vimentin expression was strongly up-regulated following loss of Syk in the MCF10A cell lines. Vimentin loss is associated with the invasive and mesenchymal phenotype observed following EMT in multiple breast cancer cell types [19], [28]. Our observations of spontaneous EMT and vimentin upregulation in subconfluent cultures of MCF10a are consistent with previous reports [29]–[33] and we now demonstrate that both can be suppressed by Syk. Interestingly, although vimentin expression in cell lines is correlated with their invasive capacity, vimentin filament expression was not correlated with invadopodia formation and degradation in individual cells. This is consistent with the report that although vimentin is associated with early sites of formation of podosomes in smooth muscle and osteoclasts, it does not seem to be directly involved in podosome formation and vimentin does not localize at the puncta of actin [34], [35]. Thus, a more intensive study must be undertaken to determine the molecular relationship between vimentin and invadopodia structures and cell motility and invasion.

Microarray analysis revealed that Syk regulates a large number of genes in the TGFβ1, NFκB, and EGFR pathways and that TGFβ1 and VEGF are upregulated. These pathways constitute important regulators of cell invasion. In particular, TGFβ-mediated EMT requires NF-κB [36]. MCF10CA1, a tumor forming cell line derived from MCF10A, has been reported to form invadopodia and undergo EMT in a process requiring PI3K and c-Src in response to TGFβ1 [37], [38]. Both PI3K and NF-κB are reported to be inhibited by Syk in breast cancer cell lines [7] and they contribute to invadopodia formation [15] directly or indirectly. And, EGFR activity is downregulated by Syk as is the downstream kinase c-Src [4], [8]. Thus, our results reveal that Syk is likely to control a TGFβ1 signaling pathway in addition to EGFR/NFκB and their interacting genes (Figures S3–S4). Thus, Syk potentially regulates at least three major signaling pathways to suppress proliferation and invasion. These pathways have important functions in the development and maintenance of the normal mammary gland and their disruption gives rise to EMT, invadopodia formation, and cancer progression. Consequently, the significance of the overlap between invadopodia-related proteins and cell surface stem cell markers (CD44 and CD49F) is not known but is highly suggestive of the involvement of invadopodia cell surface proteins in stem/progenitor cell function during development.

In conclusion, the present study demonstrates for the first time that the presence of wild type levels of Syk in normal breast epithelial cells is critical to suppress proliferation and invasion, and that partial loss promotes hyperplasia and the eventual formation of mammary tumors *in vivo*. The significance of oral or locally applied Syk inhibitors for breast cancer risk is of critical concern in the context of the current development of agents targeting Syk for therapeutic treatment of allergy, asthma and autoimmune disease [39], [40].

## Materials and Methods

### Mice

#### Ethics Statement

All animals were handled in strict accordance with good animal practice as defined by the Georgetown University Care and Use of Animals Committee (GUGUAC, protocol no. 07–020) and by the Association for the Assessment and Accreditation of Laboratory Animal Care (AAALAC).

Syk heterozygous (+/−) 129 background mice [41] were crossed with homozygous eGFP ^+/+^ (Enhanced Green Fluorescent Protein) transgenic FVB mice and the progeny genotyped for Syk. eGFP mice were purchased from Jackson Laboratory (Bar Harbor, Maine). Syk ^+/−^/eGFP ^+/+^ were back crossed six times with homozygous eGFP ^+/+^ mice. Homozygous eGFP ^+/+^ mice were selected based on fluorescence intensity. These mice were genotyped for Syk and the Syk ^+/−^/eGFP ^+/+^ (referred to as Syk ^+/−^) were used for this study. Genotypes of the mice were confirmed by PCR analysis using the oligomers 5′-AGA GAA GCC CTG CCC ATG GAC-3′, 5′-CCT TGG GAA AAG CGC CTC CCC TAC CC -3′ and 5′-GTC CAG GTA GAC CTC TTT GGG C-3′. Methods for microscopic analysis of mammary gland morphologies are found in *Supplementary File S1*.

#### Cell culture

MCF10A [42], MCF10AneoT [43], and MCF10DCIS.com [44] cell lines were acquired from Barbara Ann Karmanos Cancer Institute (obtained at passage number 174, 49, 30, respectively), passaged upon approaching confluence using standard techniques and were maintained in complete media (DMEM/F12 (50∶50 mix) supplemented with 5% horse serum, 10 mM HEPES, 10 µg/ml insulin, 20 ng/ml epidermal growth factor, 0.5 µg/ml hydrocortisone, 100 ng/ml cholera toxin). Details and minor modifications of the methods for culture of cells on collagen I matrices, Matrigel, soft agar, and primary culture of mouse mammary epithelial cells and the assays used to quantify proliferation, migration, invasion, and morphologies are found in the *Supplementary File S1* section.

#### Microarray Analysis

Global gene expression profiles were measured using Affymetrix U133A 2.0 microarrays (with 22,283 probe sets) in MCF10A cell lines (control and Syk siRNA knockdown cells on collagen gels or plastic). Total RNA was extracted from cells seeded on collagen or plastic, using standard TRIZOL-based techniques. Fragmented cRNA was prepared as described in the Affymetrix Genechip Expression Analysis Technical Manual. Hybridization, washing, and imaging steps were performed in the Lombardi Comprehensive Cancer Center Macromolecular Shared Resource. Raw images were pre-processed using Microarray Suite 5.0 software (Affymetrix, Santa Clara, CA). For the preprocessing of the microarray data, we used SemiRMA [45] for background adjustment, loess for normalization and median polish for summarization. The preprocessed data (log-intensities) were then analyzed using a two-way ANOVA model with interaction to assess differential gene expression. The two factors are indicators for collagen coated dish (versus plastic dish) and Syk siRNA (versus empty vector). Due to the small number of replicates (3), we used empirical Bayes approach to borrow information across genes [46]. The method of Benjamini-Hochberg [47] was used to control FDR at 5%. Bioconductor packages “affy” and “limma” were used in the analysis. All data is MIAME compliant. Microarray data reported herein have been deposited at the NCBI Gene Expression Omnibus (http://www.ncbi.nlm.nih.gov/geo/) with the accession number GSE16200.

#### Other statistical analysis

Results are shown as the mean ± standard error of the mean (S.E.M.). Statistical significance was calculated by using the Student's *t*-test and *P*<0.05 was accepted as a significant value.

## Supporting Information

File S1 Additional material and methods.
(0.09 MB DOC)

Table S1 Gene probes differentially regulated following Syk knockdown in MCF10A cells cultured on collagen. MCF10A cells cultured on collagen were subjected to microarray analysis to determine gene probe sets whose expression was differentially regulated following Syk knockdown. 1136 probe sets were significantly up- or down-regulated at the FDR level of 0.05 as described in Materials and Methods.
(0.18 MB PDF)

Table S2 List of gene probe sets from merged networks 1, 2, and 10. Gene probe sets that were differentially regulated following Syk knockdown of MCF10A cells cultured on collagen (Supplementary Table 1) were submitted to Ingenuity for analysis. Networks 1, 2 and 10 identified by analysis with Ingenuity were merged and the gene probes listed here. The majority of entries were represented by gene probe sets whose expression was differentially regulated following Syk knockdown of MCF10A cells cultured on collagen as indicated.
(0.01 MB PDF)

Table S3 Invadopodia gene probes that were differentially regulated following Syk knockdown in cells cultured on collagen. 139 gene probes were linked with 63 invadopodia-related proteins (proteins whose role in invadopodia function was determined by the criteria of matrix degradation on gelatin crosslinked films or localization of MT1-MMP at invadopodia [1]). Levels of 17 gene probes were significantly changed at the FDR level of 0.05 as described in Materials and Methods.
(0.01 MB PDF)

Figure S1 Time course of scrape/wound closure for MCF10A, MCF10AneoT, and DCIS.com human mammary epithelial cells. A scratch wound was made and the degree of closure achieved over 24-hr incubation was imaged by time lapse microscopy and measured using Metamorph Image Analysis software. Graphs and representative images from cell lines MCF10A (A), MCF10AneoT (B), and DCIS.com (C) are shown at 0 hr and 16 hrs. Scale bars correspond to 75 µm in all panels.
(1.06 MB TIF)

Figure S2 Soft agar growth assays. In soft agar assays, each of the Syk shRNA knockdown cell lines experienced increased colony formation. Representative images are shown. Scale bars are all 30 µm.
(0.40 MB TIF)

Figure S3 Detailed map of three merged networks. Cells cultured on collagen were analyzed to determine up- or down-regulated gene probes following Syk knockdown. TGFβ1, NFκB, and EGFR constitute major “hubs” of three networks (networks 1, 2 and 10) identified by Ingenuity (Supplementary Table 2). The links between these three networks are shown in yellow. The intersection of the invadopodia network (not shown) with the EGFR-NFκB-TGFβ1 linked networks is indicated by dashed blue lines. Probe sets up-regulated are shown in red, those down-regulated are shown in green.
(0.98 MB TIF)

Figure S4 Summary of major nodes in three networks. A summary of the major nodes of interest in networks 1, 2, and 10 are shown, taken from Supplementary Figure 1. Orange lines indicate interactions among the networks.
(0.37 MB TIF)

Figure S5 EGFR, invadopodia, and cell surface invadopodia/stem cell markers. (A) Extracts of MCF10A, MCF10AneoT, and DCIS.com transfected with control or Syk siRNA were probed for anti-pY1045-EGFR, and then stripped and re-probed for anti-EGFR and then again for anti-α-actin. Overall, activated EGFR was up-regulated by Syk knockdown. The last two lanes are two different loadings of EGF-treated MCF10A positive control cell lysates. (B) Images from the gelatin-degradation assay for invadopodia from MCF10A cells from three color confocal imaging (phalloidin, green; cortactin, blue; gelatin, magenta). Higher magnification images selected are shown here and indicated in Fig. 4D by the box. Scale bars  = 10 µm. (C). Two color flow cytometry for CD44/CD24 (CD44) and CD49f (α6 integrin)/CD24 (CD49f) demonstrates increased cell surface CD44 and CD49f with unchanged or slightly decreased cell surface CD24 following Syk knockdown by siRNA and culture of MCF10A cells on plastic.
(2.17 MB TIF)

Figure S6 Enhanced branching morphogenesis in mammary glands of Syk +/− mice (129 background). (A) Mammary gland branching and end buds are more prominent in Syk +/− heterozygote. Scale bars correspond to 150 µm in all panels. The average number of total tube area (B) and buds (C) were counted in mammary glands from Syk +/+ wild type and Syk +/− heterozygous mice. (D) Syk protein is decreased in Western blots of mammary glands extracts (minus the mammary gland lymph node) and spleen from 12-week virgin Syk +/− heterozygous compared with Syk +/+ wild type females. Spleen extracts were used as positive control.
(0.83 MB TIF)

Figure S7 Enhanced branching morphogenesis in mammary glands of Syk +/− mice. (A) Immunohistochemistry staining using N-19 rabbit polyclonal anti-Syk antibody was performed on paraffin sections of mammary glands from 10 week females. Decreased expression of Syk protein is observed in mammary epithelial cells in Syk +/− heterozygous versus Syk +/+ wild type mice. Mammary gland lymph nodes (LN) serve as internal controls where Syk is expressed in different subpopulations of lymphocytes. Adjacent ducts are indicated by arrows. Higher magnification views of end buds are shown below, illustrating positively stained luminal epithelium. Images were taken using identical microscope settings for comparison. In the lower panel, scale bars correspond to 10 µm and in their higher magnification insets to 30 µm. (B) Mammary glands from Syk +/+ wild type and Syk +/− heterozygote knockout mice of 10 week (10 wk) virgin females and late pregnancy (LP) females were dissected out and stained with carmine red in whole mounts. Mammary gland branching and end buds are more prominent in Syk +/− heterozygote as illustrated in these micrographs. Scale bars correspond to 150 µm in all panels.
(4.42 MB TIF)
