# Supplementary material for: Tumor Suppressor Function of Syk in Human MCF10A In Vitro and Normal Mouse Mammary Epithelium In Vivo
Source: PLoS One. 2009 Oct 15;4(10):e7445. doi: 10.1371/journal.pone.0007445 (PMC2759536; doi:10.1371/journal.pone.0007445)
Supplement: File S1 — Additional material and methods. (0.09 MB DOC) [file pone.0007445.s001.doc]

**Supplementary Materials and Methods.**

**Antibodies and Chemicals.** Syk N-19 antibody was from Santa Cruz Biotechnology (Santa Cruz, CA), mouse anti-actin antibody was from Chemicon International (Temecula, CA), monoclonal mouse anti-vimentin was from SIGMA (St Louis, MO), anti-pY1045 epidermal growth factor receptor and anti- epidermal growth factor receptor were from Upstate (Lake Placid, NY), and mouse anti-Syk 4D10 was purchased from NeoMarkers (Fremont, California). Goat anti-mouse secondary and donkey anti-rabbit peroxidase-conjugated IgG were from Jackson Immunoresearch Laboratories, Inc. (West Grove, PA). Anti-CD24-PE (BD Biosciences), anti-CD44-PE-Cy5 (BD Biosciences), and anti-CD49-FITC (BD Biosciences) were used for flow cytometry.

Triton X-100 and Nonidet P-40 were purchased from Sigma (St Louis, MO); and ECL reagents and Hyperfilm-ECL were from GE Healthcare (Piscataway, NJ). Nitrocellulose membranes were from Schleicher and Schüell (Dassel, Germany). BD Fluoroblok insert (non-coated, 8μm pore size, 24 well format), BD 24 well companion plates, and collagen (Bovine, Type I) were purchased from BD Biosciences (San Diego, CA). Growth factor reduced Matrigel was obtained from (BD Biosciences, San Jose, CA).

**Cell lines.** MCF10 arose spontaneously in culture from primary cells derived from a patient with fibrocystic disease and an adherent cell line MCF10A was established [10]. A number of related cell lines have been derived from MCF10A providing models of preneoplastic and neoplastic progression [5]. MCF10AneoT (MCF10AT) were generated by transformation with T24-*Ha-ras* [3]. These cells when injected with Matrigel into nude mice will form persistent tumors of varying ductal cell types and with a range from hyperplasia, to atypical hyperplasia, DCIS and eventually invasive carcinoma. The stem-cell-like features of these cells have been noted [3] and the MCF10AneoT cells have the interesting property of expressing functional estrogen receptors in contrast to the parent MCF10A cells which are ER negative [for review see [5]]. Following multiple passage and selection in animals, one clone with comedo-DCIS morphology following tumor formation was isolated, the DCIS.com line [8].

**Whole mount staining and histological analysis.** For whole mount analysis, mammary glands were removed and spread onto glass slides, fixed with 100% ETOH/glacial acetic acid (3:1) fixative and stained with carmine alum, dehydrated, cleared in xylene, and mounted with Permount. Whole mounts were examined and photographed under a stereo fluorescence microscope (Nikon SMZ1500) or using multiphoton microscopy (see below). For histological analysis, mammary glands were fixed in 10% buffered formalin (3.7% formaldehyde), embedded in paraffin, sectioned, and stained with hematoxylin and eosin (H&E) or double immunostained using anti-keratin 14 and anti-Ki67 antibodies followed by fluorescent secondary antibodies and counterstained with DAPI.

**Western blot analysis.** The following method was used in the extraction of protein from both the mammary gland (after removal of the lymph node) and spleen. In each case, the sample tissue was homogenized using 1.0 ml of RIPA buffer supplemented with complete protease inhibitor tablet (Roche Diagnostics) (Radioimmunoprecipitation assay (RIPA) buffer: 50 mM Tris (pH 8.0), 0.1% SDS, 1% NP40, 0.5% deoxycholate, 150 mM NaCl) and centrifuged at 4°C for 10 min to remove tissue and cell debris. Polypeptides were separated using 4 - 20% Tris-HCl Criterion TM Precast Gels (Bio-Rad Laboratories, Hercules, CA) and run until the samples reached the bottom of the gel. The polypeptides were transferred to a nitrocellulose membrane, blocked in 5% dry milk in PBS and incubated in primary anti–Syk antibody (N-19, 1: 200 dilution, rabbit polyclonal IgG, Santa Cruz Biotechnology) or mouse anti-Syk 4D10 (1:2000) followed by secondary HRP-conjugated antibody. The blots were developed, imaged, and then stripped and immunostained with antibodies against alpha-actin as loading controls.

**Syk siRNA transfection and culture on collagen.** A pool of Syk small interfering RNA (siRNA) duplexes were designed to target human Syk (accession no. NM_003177; Ambion, Austin, TX). As a negative control, cells were transfected with a nonspecific siRNA pool (Dharmacon, Lafayette, CO). All siRNA transfections were done with Lipofectamine 2000 (Invitrogen, Carlsbad, CA). MCF10A cell lines were seeded in complete medium at 40-50% confluency in a six well plate and incubated at 37 ^o^C overnight. The next morning, a confluency of 70-75% was reached. siRNA was diluted to 250 μl with OPTIMEM for each transfection (Invitrogen). Lipofectamine 2000 was pre-equilibrated by diluting 10 μl of Lipofectamine 2000 to 250 μl with OPTIMEM. Then, 0.5 ml containing both DNA and liposome complex was added and the plate was gently swirled for uniform mixing. After 6 hrs, the media was replaced with 2.5 ml of complete medium. The plate was incubated for 48 hrs at 37 ^o^C and cells were collected using trypsin and then washed in complete medium. Cells were placed on a layer of bovine collagen gel (3.0 mg/ml) or on plastic tissue culture dishes without collagen and cultured for an additional 20 hrs at 37 ^o^C.

**MTS assay.** The MTS assay is composed of solutions of a tetrazolium compound (3-(4, 5-dimethylthiazol-2-yl)-5-(3-carboxymethoxyphenyl)-2-(4-sulfophenyl)-2H-tetrazolium, inner salt; MTS) and an electron coupling reagent (phenazine methosulfate; PMS). MTS is bioreduced by cells into a formazan product that is soluble in tissue culture medium. Optical density (OD) in the absorbance of the formazan at 490nm was measured as an indicator of cell proliferation.

**Matrigel assay.** Growth factor reduced Matrigel was used to perform a three dimensional (3D) morphogenesis assay of MCF10A cells or primary cells [7,4]. This assay was performed in a 4-well chamber (Lab-Tek® II chamber #1.5 German, Nalge Nunc International Corp., Naperville, IL). The culture has two matrix layers and is the “on-top” method [6]. First, an underlying layer of 50 μL of Matrigel is established and allowed to set at 37 ^o^C. Cells were mixed with basal medium (DMEM/F12 (50:50) medium) containing 2 % Matrigel and 5 ng/ml EGF, and then the cells in 80 μL are layered on top of the Matrigel layer (final concentration 5,000 cells/well) followed by an incubation of 30 min at 37 ºC. All wells were then treated with 200 μl of basal media for 48 hrs. Every other day all samples were replenished with basal media. Cell morphology was monitored by phase contrast microscopy. Cysts formed in Matrigel after 48 hours were imaged using phase contrast microscopy and object area and object fiber lengths were determined using Metamorph Image analysis software ver. 7.0.

**Lentiviral shRNA.** Constructs for stable depletion of Syk were obtained from the RNAi Consortium [9] via SIGMA-Aldrich (NM_003177). For the Syk gene, five pre-made constructs were obtained and individually tested to identify those able to achieve efficient knockdown at the protein level. Negative control constructs in the same vector system (vector alone, pLKO.1 puro) were obtained from SIGMA-Aldrich (NM_003177). The lentiviral helper plasmids pHR′8.2ΔR and pCMV-VSV-G were also obtained from Robert Weinberg via Addgene. All plasmids were prepped using the QIAGEN Maxi Prep kit and their quality was confirmed on an agarose gel. The integrity of all shRNA inserts was confirmed by sequencing.

To prepare transient virus stocks, 1.5 × 10^6^ 293T cells were plated in 10-cm dishes. The next day, the cells were co-transfected with shRNA constructs (3 μg), together with pHR′8.2ΔR and pCMV-VSV-G helper constructs (3 μg and 0.3 μg, respectively), using FuGENE 6 (Roche, Indianapolis, IN). The media were changed the next day, and the following day, virus-containing media was harvested. The viral stocks were centrifuged and filtered (0.45 micrometer filter) to remove any nonadherent 293T cells.

Next, MCF10A, MCF10AneoT, DCIS.COM cells were infected with shRNA lentiviruses. To do this, the cells were plated at sub confluent densities. The next day, the cells were infected with a cocktail of 500 μl virus-containing medium, 1 ml regular medium, and 8 μg/ml Polybrene. The medium was changed 1 day post-infection, and selection medium was added 2 days post-infection (5 μg/ml puromycin for MCF10A, MCF10AneoT, DCIS.COM cells). After 3 days of puromycin selection, the mock-infected cells had all died. Stably infected pooled clones were studied.

**Soft Agar Growth Assay**. 5,000 cells were plated in 0.3% agar layered on top of 0.6% agar in a 6 well plate. After 2 weeks, colonies were stained with 0.005% crystal violet and images taken to count and measure colony size using MetaMorph Offline Image Analysis software, ver. 7.0 (Molecular Devices, Sunnyvale, CA).

**Fluorescent-gelatin degradation assay.** To assess the ability of cells to form invadopodia and degrade matrix, cells were plated on coverslips coated with fluorescent gelatin matrix [2,1] in 12-well plates at 75 x 10^3^/mL per well and incubated at 37 °C. Confocal images were collected using a Zeiss LSM510/META/NLO laser scanning confocal microscope (Carl Zeiss, Thornwood, NY) with Plan-Apochromat 63x/1.4 N.A. oil objective. Foci of degraded matrix were visible as dark areas or "holes" in the bright fluorescent gelatin matrix. The area of the holes per cell was determined using Metamorph Image analysis software ver. 7.0, Count Nuclei application to identify holes, and then thresholding for these objects in the resulting segmented image to determine total area of holes. Cells were automatically counted by identifying DAPI-stained nuclei.

**Wounding assay.** Migration of MCF10A, MCF10AneoT, and MCF10DCIS.com cells following Syk siRNA knockdown was assessed by measuring the movement of cells into a scraped area, a "wound assay". Cells were imaged immediately after scraping using a Nikon TE300 microscope with environmental chamber and equipped with a 10X lens. Time lapse imaging was controlled using the Multidimensional Analysis tool of Metamorph Image Acquisition software. The assay was terminated when the first wounds were completely closed, which occurred at 16~48 h. Velocities were determined using the Track Points application of Metamorph applied to calibrated images.

**Flow cytometry**. Cells were fixed in 70% ethanol and stained in PBS containing 0.1% Triton X-100, 50 μg/mL RNase, and 50 μg/mL propidium iodide. DNA content was measured on a FACSort flow cytometer (Becton Dickinson, Franklin Lakes, NJ), and data were analyzed using ModFit software (Verity Software House, Topsham, ME). At least 1x10^6^ cells were analyzed per sample.

To perform the analysis of cell surface markers (CD44+/CD24-, CD49+/CD24-), MCF10A cells were detached with trypsin, washed in blocking buffer (PBS containing 3% FBS), then stained with anti-CD24-PE (BD Biosciences, San Diego, CA, USA) and anti-CD44-PE-Cy5 (BD Biosciences) or anti-CD49-FITC (BD Biosciences) using 1 μl of antibody per 10^6^ cells, and incubated at room temperature for 1 hr. Following incubation, cells were washed twice with 1 ml PBS. Cells were re-suspended in 1 ml PBS and then were analyzed by flow cytometry using the FACSort (Becton Dickinson, San Jose, CA, USA). Data were collected using ModFit software (Verity Software House, Topsham, ME).

**Boyden chamber chemoinvasion assay.** Invasion of MCF10A, MCF10AneoT, and MCF10DCIS.com cells following siRNA knockdown was assessed using BD Fluoroblok inserts (non-coated, 8μm pore size, 24-well format, BD Biosciences). Sterile forceps were used to remove cell wall inserts from their packaging and place them into wells of a 24 well plate. 30μl of 1:5 diluted Matrigel was added to the center of each cell well inserts. Coated inserts were placed in the incubator to allow the Matrigel to solidify for 20-30 min. 2 X10^5^ cells per sample were plated in 200 μl DMEM/F12 into the upper chamber. Immediately 400 μl of complete medium containing 20 nM EGF was added to each well in the lower chamber. EGF acted as a chemo-attractant for the cells. After 36 hours, cells that passed through the pores on the membranes and attached themselves to the bottom side of the membranes, were stained with Calcein AM (Invitrogen) and fixed using 10% formalin/0.1 % Triton X-100. The underside of the membranes was imaged using the 25X/0.8 N.A. lens on the Zeiss LSM510 microscope. Area of cells per image was measured using MetaMorph and used to determine the number of migrated cells.

**Collagen gel assay.** A pre-set layer of bovine collagen gel (Final volume 25 μl per well; final collagen concentration 3.0 mg/ml, 10X Optimem (pH 7.0), 0.027% Sodium Bicarbonate, 0.1% 1X antibiotic) was prepared in wells of a 96-well plate. It was allowed to polymerize for 30 minutes at 37 ºC. Cells (control or Syk siRNA transfected MCF10A cells, 10,000 cells/well) were layered on top of the pre-set layer and were allowed to adhere. After 30 minutes at 37 ºC, an additional 50 μl collagen was added. It was allowed to polymerize for 30 minutes at 37 ºC and then the collagen-cell sandwich was covered with complete media and incubated at 37 ºC for an additional 20 hours. The collagen gel was fixed using 10% formalin/0.1% Triton X-100 in PBS for 30 min at room temperature. AlexaFluor 488-phalloidin (1:200 dilution) was applied and confocal images were obtained using a Zeiss 25X/ 0.8 N.A. lens on a Zeiss LSM510/META confocal microscope. Metamorph was used to determine object size and shape (circularity).

**Primary culture.** The 4^th^ inguinal mammary glands were removed from 8 weeks old virgin GFP ^+/+^ / Syk ^+/+^ and GFP ^+/+^ / Syk ^+/-^ mice and minced with blades. Minced tissue (4 glands) was gently shaken for 1.5 hrs at 37 °C in a 10 ml 1 X collagenase mixture **(**0.001% penicillin/streptomycin, 10μg/ml insulin, 5% fetal bovine serum, 0.5 μg/ml Fungizone, 10 μ/ml gentamycin, collagenase final concentration 500 U/ml, hyaluronidase final concentration 500 U/ml in 10 ml of DMEM/F12 (1:1)). The collagenase solution was discarded after centrifugation at low speed for 10 min and the pellet was re-suspended in 10 ml DMEM/F12**.** The suspension was pelleted again for 10 min, re-suspended in 4 ml of DMEM/F12 +40 μl of DNase (2U/ μl) and incubated for 5 min at ambient temperature with shaking. The DNase solution was removed after centrifugation at low speed for 10 min. The DNase solution was discarded and the epithelial pieces were separated from the single cells through differential centrifugation. The pellet was re-suspended in 10 ml of DMEM/F12 and pulsed to at low speed. The supernatant was then removed and the pellet was re-suspended in 10 ml DMEM/F12. Differential centrifugation was performed at least 4 times. The final pellet was resuspended in the desired amount of medium or Matrigel (growth factor reduced Matrigel, BD Biosciences)

**Confocal and Multiphoton Microscopy**. Confocal images were collected using a Zeiss LSM510/META/NLO laser scanning microscope (Carl Zeiss, Thornwood, NY) with Zeiss Plan-Neofluar 100x/1.3 N.A. oil or Plan-Apochromat 63x/1.4 N.A. oil objectives. Dapi staining was imaged at 770 nm excitation of the Ti-Sapphire laser and emission of 435-485 nm, AlexaFluor488 was imaged with excitation of 488 nm using an Argon laser and emission of 500-530 nm, and AlexaFluor568 or Cy3 stained samples were imaged with excitation of 543 nm using a HeNe laser and emission of 565-615 nm. For imaging of Cy5 conjugated secondary antibodies, excitation was 633 nm using a HeNe laser and emission was collected at 650-704 nm. For multiphoton imaging of carmine red fluorescence, excitation of the Ti-Sapphire Cameleon XR laser was set to 750 nm and emission was collected in the red channel, 565-615 nm. Images were collected using a Zeiss 25X/ 0.8 N.A. lens.

## References

1 Artym, V V, Yamada, K M, & Mueller, S C (2009) Methods Mol.Biol. 522:211-9.: 211-219.

2 Artym, V V, Zhang, Y, Seillier-Moiseiwitsch, F, Yamada, K M, & Mueller, S C (2006) Cancer Res. 66: 3034-3043.

3 Dawson, P J, Wolman, S R, Tait, L, Heppner, G H, & Miller, F R (1996) Am.J.Pathol. 148: 313-319.

4 Debnath, J, Muthuswamy, S K, & Brugge, J S (2003) Methods 30: 256-268.

5 Heppner, G H, Miller, F R, & Shekhar, P M (2000) Breast Cancer Res. 2: 331-334.

6 Lee, G Y, Kenny, P A, Lee, E H, & Bissell, M J (2007) Nat.Methods. 4: 359-365.

7 Lee, G Y, Kenny, P A, Lee, E H, & Bissell, M J (2007) Nat.Methods. 4: 359-365.

8 Miller, F R, Santner, S J, Tait, L, & Dawson, P J (2000) J.Natl.Cancer Inst. 92: 1185-1186.

9 Moffat, J, Grueneberg, D A, Yang, X, Kim, S Y, Kloepfer, A M et al. (2006) Cell. 124: 1283-1298.

10 Soule, H D, Maloney, T M, Wolman, S R, Peterson, W D, Jr., Brenz, R et al. (1990) Cancer Res. 50: 6075-6086.
