# Supplementary material for: Tumor Suppressor Function of Syk in Human MCF10A In Vitro and Normal Mouse Mammary Epithelium In Vivo
Source: PLoS One. 2009 Oct 15;4(10):e7445. doi: 10.1371/journal.pone.0007445 (PMC2759536; doi:10.1371/journal.pone.0007445)
Supplement: Table S1 — Gene probes differentially regulated following Syk knockdown in MCF10A cells cultured on collagen. MCF10A cells cultured on collagen were subjected to microarray analysis to determine gene probe sets whose expression was differentially regulated following Syk knockdown. 1136 probe sets were significantly up- or down-regulated at the FDR level of 0.05 as described in Materials and Methods. (0.18 MB PDF) [file pone.0007445.s002.pdf]

Differentially expressed genes on collagen

probesetID	Name	Symbol	logFC.col	Fold Change Collagen	adj.P.Val
205199_at	carbonic anhydrase IX	CA9	3.299453256	9.845423	0.002990116
219410_at	transmembrane protein 45A	TMEM45A	2.753742271	6.744644	0.007988424
200632_s_at	N-myc downstream regulated gene 1	NDRG1	2.688283097	6.445459	0.007043637
221478_at	BCL2/adenovirus E1B 19kDa interacting protein 3-like	BNIP3L	2.355828184	5.11888	0.003930777
204595_s_at	stanniocalcin 1	STC1	2.331592801	5.033608	0.004387256
215446_s_at	lysyl oxidase	LOX	2.227402498	4.682901	0.02543191
204597_x_at	stanniocalcin 1	STC1	2.121759431	4.352244	0.006921301
202998_s_at	lysyl oxidase-like 2 /// ectonucleoside triphosphate diphosphohydrolase 4	ENTPD4 //,	2.118145508	4.341355	0.010789862
202022_at	aldolase C, fructose-bisphosphate	ALDOC	2.083954485	4.239677	0.007308892
202219_at	solute carrier family 6 (neurotransmitter transporter, creatine), member 8	SLC6A8	2.037283638	4.10472	0.007337164
204298_s_at	lysyl oxidase	LOX	2.02909412	4.081485	0.015091187
203729_at	epithelial membrane protein 3	EMP3	1.999103197	3.997514	0.027885596
219434_at	triggering receptor expressed on myeloid cells 1	TREM1	1.94597187	3.852972	0.005627889
210854_x_at	solute carrier family 6 (neurotransmitter transporter, creatine), member 8	SLC6A8	1.853352748	3.613389	0.007271766
213397_x_at	ribonuclease, RNase A family, 4	RNASE4	1.83820444	3.575647	0.024017189
201849_at	BCL2/adenovirus E1B 19kDa interacting protein 3	BNIP3	1.808999226	3.503991	0.005627889
213843_x_at	solute carrier family 6 (neurotransmitter transporter, creatine), member 8	SLC6A8	1.758162859	3.382671	0.00887733
203963_at	carbonic anhydrase XII	CA12	1.755610704	3.376692	0.008546266
201848_s_at	BCL2/adenovirus E1B 19kDa interacting protein 3	BNIP3	1.750101671	3.363823	0.003930777
210735_s_at	carbonic anhydrase XII	CA12	1.724284151	3.304161	0.006949425
201163_s_at	insulin-like growth factor binding protein 7	IGFBP7	1.630067563	3.095275	0.014733441
215867_x_at	carbonic anhydrase XII	CA12	1.611997724	3.056748	0.007257672

215812_s_at	solute carrier family 6 (neurotransmitter transporter, creatine), member 8 /// solute carrier family 6 (neurotransmitter transporter, creatine), member 10 (pseudogene) /// hypothetical LOC653562	LOC65356[illegible]	1.587810585	3.005928	0.005627889
214164_x_at	carbonic anhydrase XII	CA12	1.576030486	2.981484	0.007610795
201313_at	enolase 2 (gamma, neuronal)	ENO2	1.574055942	2.977406	0.006293525
205158_at	ribonuclease, RNase A family, 4	RNASE4	1.553832302	2.93596	0.02449946
213640_s_at	lysyl oxidase	LOX	1.497766649	2.824052	0.026683706
204508_s_at	carbonic anhydrase XII	CA12	1.479552769	2.788623	0.017071662
210512_s_at	vascular endothelial growth factor A	VEGFA	1.47723116	2.784139	0.020112851
201185_at	HtrA serine peptidase 1	HTRA1	1.474539838	2.77895	0.016278635
209822_s_at	very low density lipoprotein receptor	VLDLR	1.466523662	2.763552	0.006994923
205780_at	BCL2-interacting killer (apoptosis-inducing)	BIK	1.456313137	2.744062	0.001616605
202620_s_at	procollagen-lysine, 2-oxoglutarate 5-dioxygenase 2	PLOD2	1.435060093	2.703934	0.020959453
213010_at	protein kinase C, delta binding protein	PRKCDBP	1.429329966	2.693216	0.001616605
202364_at	MAX interactor 1	MXI1	1.425838466	2.686706	0.012932821
207543_s_at	procollagen-proline, 2-oxoglutarate 4-dioxygenase (proline 4-hydroxylase), alpha polypeptide I	P4HA1	1.418199538	2.672518	0.018699219
221479_s_at	BCL2/adenovirus E1B 19kDa interacting protein 3-like	BNIP3L	1.401482172	2.641728	0.009110746
219888_at	sperm associated antigen 4	SPAG4	1.366358041	2.578189	0.004926805
205364_at	acyl-Coenzyme A oxidase 2, branched chain	ACOX2	1.357728182	2.562813	0.004895765
219232_s_at	egl nine homolog 3 (C. elegans)	EGLN3	1.332916288	2.519114	0.008546266
207030_s_at	cysteine and glycine-rich protein 2	CSRP2	1.315815549	2.48943	0.005627889
218507_at	hypoxia-inducible protein 2	HIG2	1.295911215	2.45532	0.001616605
202619_s_at	procollagen-lysine, 2-oxoglutarate 5-dioxygenase 2	PLOD2	1.278971216	2.426659	0.026201143

215813_s_at	prostaglandin-endoperoxide synthase 1 (prostaglandin G/H synthase and cyclooxygenase)	PTGS1	1.277701242	2.424524	0.006293525
201162_at	insulin-like growth factor binding protein 7	IGFBP7	1.267966477	2.408219	0.023195616
202464_s_at	6-phosphofructo-2-kinase/fructose-2,6-biphosphatase 3	PFKFB3	1.265426572	2.403983	0.009825362
210933_s_at	fascin homolog 1, actin-bundling protein (Strongylocentrotus purpuratus)	FSCN1	1.262105186	2.398455	0.039958616
203085_s_at	transforming growth factor, beta 1	TGFB1	1.261478857	2.397414	0.015628504
205128_x_at	prostaglandin-endoperoxide synthase 1 (prostaglandin G/H synthase and cyclooxygenase)	PTGS1	1.253758494	2.384619	0.007257672
221123_x_at	zinc finger protein 395	ZNF395	1.242957854	2.366833	0.022792248
213201_s_at	troponin T type 1 (skeletal, slow)	TNNT1	1.240175725	2.362273	0.01323345
205141_at	angiogenin, ribonuclease, RNase A family, 5	ANG	1.219137479	2.328075	0.012973447
209230_s_at	nuclear protein 1	NUPR1	1.189803901	2.281217	0.035341012
203574_at	nuclear factor, interleukin 3 regulated	NFIL3	1.188370314	2.278952	0.005627889
218149_s_at	zinc finger protein 395	ZNF395	1.153308196	2.224233	0.03601083
209183_s_at	chromosome 10 open reading frame 10	C10orf10	1.130009521	2.188602	0.030495392
204596_s_at	stanniocalcin 1	STC1	1.117980918	2.17043	0.005646347
202236_s_at	solute carrier family 16, member 1 (monocarboxylic acid transporter 1)	SLC16A1	1.109297398	2.157406	0.020505006
201250_s_at	solute carrier family 2 (facilitated glucose transporter), member 1	SLC2A1	1.091455997	2.13089	0.022794017
202234_s_at	solute carrier family 16, member 1 (monocarboxylic acid transporter 1)	SLC16A1	1.076506669	2.108923	0.022346379
208753_s_at	nucleosome assembly protein 1-like 1	NAP1L1	1.071212806	2.101199	0.030495392
209566_at	insulin induced gene 2	INSIG2	1.064253158	2.091087	0.0102878
209900_s_at	solute carrier family 16, member 1 (monocarboxylic acid transporter 1)	SLC16A1	1.063229562	2.089604	0.037121712
203643_at	Ets2 repressor factor	ERF	1.062395991	2.088397	0.00887733

222238_s_at	polymerase (DNA directed), mu	POLM	1.06218116	2.088086	0.009110746
203409_at	damage-specific DNA binding protein 2, 48kDa	DDB2	1.056348239	2.079661	0.005627889
208716_s_at	transmembrane and coiled-coil domains 1	TMCO1	1.053289209	2.075256	0.028694993
218384_at	calcium regulated heat stable protein 1, 24kDa	CARHSP1	1.046935815	2.066137	0.030686502
213629_x_at	metallothionein 1F	MT1F	1.030803568	2.043162	0.004926805
203851_at	insulin-like growth factor binding protein 6	IGFBP6	1.026105036	2.036519	0.023508348
211126_s_at	cysteine and glycine-rich protein 2	CSRP2	1.025244645	2.035304	0.003930777
204955_at	sushi-repeat-containing protein, X-linked	SRPX	1.023912984	2.033427	0.022122228
221497_x_at	egl nine homolog 1 (C. elegans)	EGLN1	1.014335734	2.019973	0.001616605
202761_s_at	spectrin repeat containing, nuclear envelope 2	SYNE2	1.008012991	2.011139	0.028522438
202997_s_at	lysyl oxidase-like 2 /// ectonucleoside triphosphate diphosphohydrolase 4	ENTPD4 //,	0.999880743	1.999835	0.022122228
209651_at	transforming growth factor beta 1 induced transcript 1	TGFB1I1	0.992759749	1.989988	0.013967851
218145_at	tribbles homolog 3 (Drosophila)	TRIB3	0.976221875	1.967307	0.013529536
202422_s_at	acyl-CoA synthetase long-chain family member 4	ACSL4	0.974600654	1.965097	0.041260074
210524_x_at			0.967645307	1.955646	0.0102878
201397_at	phosphoglycerate dehydrogenase	PHGDH	0.966451538	1.954029	0.015364265
203282_at	glucan (1,4-alpha-), branching enzyme 1 (glycogen branching enzyme, Andersen disease, glycogen storage disease type IV)	GBE1	0.953832119	1.937011	0.013682976
202686_s_at	AXL receptor tyrosine kinase	AXL	0.945469022	1.925815	0.028641503
205676_at	cytochrome P450, family 27, subfamily B, polypeptide 1	CYP27B1	0.941586364	1.920639	0.048665054
202973_x_at	family with sequence similarity 13, member A1	FAM13A1	0.940247431	1.918857	0.031124797
209373_at	mal, T-cell differentiation protein-like	MALL	0.934295333	1.910957	0.02901003
201666_at	TIMP metallopeptidase inhibitor 1	TIMP1	0.918193976	1.889748	0.016850455
211527_x_at	vascular endothelial growth factor A	VEGFA	0.910792186	1.880078	0.030569297

206686_at	pyruvate dehydrogenase kinase, isozyme 1	PDK1	0.906138634	1.874023	0.005627889
201983_s_at	epidermal growth factor receptor (erythroblastic leukemia viral (v-erb-b) oncogene homolog, avian)	EGFR	0.903019693	1.869976	0.020225356
204326_x_at	metallothionein 1X	MT1X	0.896842796	1.861987	0.035308672
200771_at	laminin, gamma 1 (formerly LAMB2)	LAMC1	0.885189062	1.847007	0.034949293
202952_s_at	ADAM metallopeptidase domain 12 (meltrin alpha)	ADAM12	0.884018669	1.845509	0.032075577
221864_at	ORAI calcium release-activated calcium modulator 3	ORAI3	0.879990072	1.840363	0.030816874
217165_x_at	metallothionein 1F	MT1F	0.863670826	1.819662	0.021162931
208703_s_at	amyloid beta (A4) precursor-like protein 2	APLP2	0.854780138	1.808483	0.018190072
217047_s_at	family with sequence similarity 13, member A1	FAM13A1	0.853251233	1.806568	0.025799468
204900_x_at	Sin3A-associated protein, 30kDa	SAP30	0.84938113	1.801728	0.017917278
206400_at	lectin, galactoside-binding, soluble, 7 (galectin 7) /// galectin-7 /// similar to Galectin-7 (Gal-7) (HKL-14) (PI7) (p53-induced protein 1)	GAL7 /// L(	0.841699101	1.79216	0.049729879
212171_x_at	vascular endothelial growth factor A	VEGFA	0.833348452	1.781816	0.028320515
207390_s_at	smoothelin	SMTN	0.832912649	1.781278	0.048877822
208704_x_at	amyloid beta (A4) precursor-like protein 2	APLP2	0.817743579	1.762647	0.030883173
201034_at	adducin 3 (gamma)	ADD3	0.817223252	1.762011	0.042597818
202833_s_at	serpin peptidase inhibitor, clade A (alpha-1 antiproteinase, antitrypsin), member 1	SERPINA1	0.817206769	1.761991	0.047872736
212647_at	related RAS viral (r-ras) oncogene homolog	RRAS	0.81249522	1.756246	0.003901445
211098_x_at	transmembrane and coiled-coil domains 1	TMCO1	0.809627965	1.752759	0.047825402
200737_at	phosphoglycerate kinase 1	PGK1	0.805884717	1.748218	0.018190072
222217_s_at	solute carrier family 27 (fatty acid transporter), member 3	SLC27A3	0.802907605	1.744614	0.001527096
210513_s_at	vascular endothelial growth factor A	VEGFA	0.794712981	1.734732	0.023163977

219545_at	potassium channel tetramerisation domain containing 14	KCTD14	0.794257741	1.734185	0.034446605
215210_s_at	dihydrolipoamide S-succinyltransferase (E2 component of 2-oxo-glutarate complex) /// dihydrolipoamide S-succinyltransferase pseudogene (E2 component of 2-oxo-glutarate complex)	DLST /// DI	0.79379654	1.733631	0.009110746
203476_at	trophoblast glycoprotein	TPBG	0.783142293	1.720875	0.009825362
207559_s_at	zinc finger, MYM-type 3	ZMYM3	0.776392365	1.712842	0.007106782
219622_at	RAB20, member RAS oncogene family	RAB20	0.774065503	1.710082	0.001616605
201774_s_at	non-SMC condensin I complex, subunit D2	NCAPD2	0.770188419	1.705493	0.047706878
209264_s_at	tetraspanin 4	TSPAN4	0.764982197	1.699349	0.025699386
218274_s_at	ankyrin repeat and zinc finger domain containing 1	ANKZF1	0.759158108	1.692503	0.006765098
201753_s_at	adducin 3 (gamma)	ADD3	0.756328637	1.689187	0.00982943
201125_s_at	integrin, beta 5	ITGB5	0.755452205	1.688161	0.020104218
201551_s_at	lysosomal-associated membrane protein 1	LAMP1	0.75074257	1.682659	0.022760006
202336_s_at	peptidylglycine alpha-amidating monooxygenase	PAM	0.748061195	1.679534	0.04562343
210792_x_at	SIVA1, apoptosis-inducing factor	SIVA1	0.747184939	1.678514	0.024625133
209263_x_at	tetraspanin 4	TSPAN4	0.742768905	1.673384	0.009849164
200762_at	dihydropyrimidinase-like 2	DPYSL2	0.740271894	1.670491	0.02597525
205462_s_at	hippocalcin-like 1	HPCAL1	0.738746695	1.668726	0.010789862
217949_s_at	vitamin K epoxide reductase complex, subunit 1	VKORC1	0.738578046	1.66853	0.010582924
218848_at	THO complex 6 homolog (Drosophila)	THOC6	0.734780301	1.664144	0.001277571
200827_at	procollagen-lysine 1, 2-oxoglutarate 5-dioxygenase 1 /// hypothetical protein LOC100130069	LOC10013(	0.733941324	1.663177	0.016350607
203510_at	met proto-oncogene (hepatocyte growth factor receptor)	MET	0.727898718	1.656225	0.011833306
217370_x_at	fusion (involved in t(12;16) in malignant liposarcoma)	FUS	0.723232016	1.650876	0.010545841
204243_at	rearranged L-myc fusion	RLF	0.722057226	1.649533	0.003930777

202331_at	branched chain keto acid dehydrogenase E1, alpha polypeptide	BCKDHA	0.715478569	1.642028	0.014239772
202235_at	solute carrier family 16, member 1 (monocarboxylic acid transporter 1)	SLC16A1	0.712401783	1.63853	0.020104218
212552_at	hippocalcin-like 1	HPCAL1	0.711879544	1.637937	0.010853986
208964_s_at	fatty acid desaturase 1 /// fatty acid desaturase 3	FADS1 /// I	0.707958876	1.633491	0.036229085
218382_s_at	U2 small nuclear RNA auxiliary factor 2	U2AF2	0.707732838	1.633236	0.038057519
204688_at	sarcoglycan, epsilon	SGCE	0.703542574	1.628499	0.001616605
201968_s_at	phosphoglucomutase 1	PGM1	0.701851445	1.626591	0.004868451
201628_s_at	Ras-related GTP binding A	RRAGA	0.695262433	1.619179	0.004387256
209170_s_at	glycoprotein M6B	GPM6B	0.694339479	1.618143	0.015984393
214620_x_at	peptidylglycine alpha-amidating monooxygenase	PAM	0.693729258	1.617459	0.041519922
208962_s_at	fatty acid desaturase 1 /// fatty acid desaturase 3	FADS1 /// I	0.691732723	1.615222	0.021637422
204899_s_at	Sin3A-associated protein, 30kDa	SAP30	0.687010352	1.609944	0.003930777
218330_s_at	neuron navigator 2	NAV2	0.686809049	1.609719	0.049639322
213455_at	family with sequence similarity 114, member A1	FAM114A1	0.685502228	1.608262	0.008546266
205899_at	cyclin A1	CCNA1	0.68249769	1.604916	0.008103638
208858_s_at	family with sequence similarity 62 (C2 domain containing), member A	FAM62A	0.678687682	1.600683	0.017071662
218498_s_at	ERO1-like (S. cerevisiae)	ERO1L	0.677103229	1.598926	0.01808607
209318_x_at	pleiomorphic adenoma gene-like 1	PLAGL1	0.675000647	1.596597	0.020646414
212958_x_at	peptidylglycine alpha-amidating monooxygenase	PAM	0.673292243	1.594708	0.038035106
201350_at	flotillin 2	FLOT2	0.672122772	1.593416	0.030495392
218849_s_at	protein phosphatase 1, regulatory (inhibitor) subunit 13 like	PPP1R13L	0.670073844	1.591154	0.046688116
208886_at	H1 histone family, member 0	H1F0	0.669932385	1.590998	0.030228794
212094_at	paternally expressed 10	PEG10	0.669683623	1.590724	0.02449946
201984_s_at	epidermal growth factor receptor (erythroblastic leukemia viral (v-erb-b) oncogene homolog, avian)	EGFR	0.668502275	1.589422	0.011534663
221957_at	pyruvate dehydrogenase kinase, isozyme 3	PDK3	0.6672539	1.588047	0.022122228
212689_s_at	jumonji domain containing 1A	JMJD1A	0.664601435	1.58513	0.027374458

212366_at	zinc finger protein 292	ZNF292	0.664318147	1.584819	0.035341012
222001_x_at	family with sequence similarity 91, member A2	FAM91A2	0.66090354	1.581073	0.009110746
209584_x_at	apolipoprotein B mRNA editing enzyme, catalytic polypeptide-like 3C	APOBEC3C	0.660803481	1.580963	0.007435414
207717_s_at	plakophilin 2	PKP2	0.660108619	1.580202	0.031729696
210984_x_at	epidermal growth factor receptor (erythroblastic leukemia viral (v-erb-b) oncogene homolog, avian)	EGFR	0.660107665	1.580201	0.010739422
213605_s_at	hypothetical protein LOC100134282 /// hypothetical protein LOC100134401	LOC10013	0.657582852	1.577438	0.039353364
221211_s_at	chromosome 21 open reading frame 7	C21orf7	0.65738899	1.577226	0.045510962
209289_at	nuclear factor I/B	NFIB	0.655526981	1.575191	0.029644613
219203_at	chromosome 14 open reading frame 122	C14orf122	0.6491052	1.568195	0.00887733
201260_s_at	synaptophysin-like 1	SYPL1	0.648761564	1.567822	0.048665054
209285_s_at	chromosome 3 open reading frame 63	C3orf63	0.647830122	1.56681	0.030955214
217356_s_at	phosphoglycerate kinase 1	PGK1	0.646844893	1.56574	0.037095131
218383_at	chromosome 14 open reading frame 94	C14orf94	0.645898178	1.564713	0.017917278
201953_at	calcium and integrin binding 1 (calmyrin)	CIB1	0.640804297	1.559198	0.020646414
205822_s_at	3-hydroxy-3-methylglutaryl-Coenzyme A synthase 1 (soluble)	HMGCS1	0.640346811	1.558704	0.032839923
203489_at	SIVA1, apoptosis-inducing factor	SIVA1	0.635467364	1.553441	0.012973447
202472_at	mannose phosphate isomerase	MPI	0.632859228	1.550635	0.010192972
202945_at	folylpolyglutamate synthase	FPGS	0.632476481	1.550224	0.01989318
211607_x_at	epidermal growth factor receptor (erythroblastic leukemia viral (v-erb-b) oncogene homolog, avian)	EGFR	0.630835304	1.548461	0.026383236
209167_at	glycoprotein M6B	GPM6B	0.630462663	1.548061	0.020505006
219155_at	phosphatidylinositol transfer protein, cytoplasmic 1	PITPNC1	0.622972581	1.540045	0.013481296
204165_at	WAS protein family, member 1	WASF1	0.619532702	1.536377	0.017917278
205076_s_at	myotubularin related protein 11	MTMR11	0.619389924	1.536225	0.020104218

201752_s_at	adducin 3 (gamma)	ADD3	0.616054505	1.532678	0.038594859
44702_at	synapse defective 1, Rho GTPase, homolog 1 (C. elegans)	SYDE1	0.61573845	1.532342	0.012978115
210426_x_at	RAR-related orphan receptor A	RORA	0.614368173	1.530887	0.004926805
204017_at	KDEL (Lys-Asp-Glu-Leu) endoplasmic reticulum protein retention receptor 3	KDELR3	0.608131272	1.524284	0.033843707
208796_s_at	cyclin G1	CCNG1	0.60220836	1.518038	0.042324593
214835_s_at	succinate-CoA ligase, GDP-forming, beta subunit	SUCLG2	0.598552347	1.514196	0.045562906
202733_at	procollagen-proline, 2-oxoglutarate 4-dioxygenase (proline 4-hydroxylase), alpha polypeptide II	P4HA2	0.589373502	1.504593	0.029641257
215111_s_at	TSC22 domain family, member 1	TSC22D1	0.586875656	1.50199	0.016352277
210640_s_at	G protein-coupled estrogen receptor 1	GPER	0.585179154	1.500225	0.037676207
212873_at	histocompatibility (minor) HA-1	HMHA1	0.58346458	1.498443	0.022346379
221958_s_at	G protein-coupled receptor 177	GPR177	0.57615254	1.490868	0.045899526
214268_s_at	myotubularin related protein 4	MTMR4	0.576108479	1.490822	0.032430413
203439_s_at	stanniocalcin 2	STC2	0.575724561	1.490426	0.008601198
218093_s_at	ankyrin repeat domain 10	ANKRD10	0.575522323	1.490217	0.026912528
208103_s_at	acidic (leucine-rich) nuclear phosphoprotein 32 family, member E	ANP32E	0.575437006	1.490129	0.036846349
212494_at	tensin like C1 domain containing phosphatase (tensin 2)	TENC1	0.571166206	1.485724	0.015830371
200710_at	acyl-Coenzyme A dehydrogenase, very long chain	ACADVL	0.568746587	1.483234	0.004868451
203683_s_at	vascular endothelial growth factor B	VEGFB	0.56832039	1.482796	0.03871616
213664_at	solute carrier family 1 (neuronal/epithelial high affinity glutamate transporter, system Xag), member 1	SLC1A1	0.564307776	1.478678	0.013481296
218017_s_at	heparan-alpha-glucosaminide N-acetyltransferase	HGSNAT	0.562520378	1.476847	0.025223999

201719_s_at	erythrocyte membrane protein band 4.1-like 2	EPB41L2	0.562092261	1.476409	0.033843707
218592_s_at	cat eye syndrome chromosome region, candidate 5	CECR5	0.560968119	1.475259	0.022219699
206246_at	6-phosphofructo-2-kinase/fructose-2,6-biphosphatase 4	PFKFB4	0.560380405	1.474658	0.020059581
203946_s_at	arginase, type II	ARG2	0.560295986	1.474572	0.009825362
206070_s_at	EPH receptor A3	EPHA3	0.556554244	1.470752	0.047825402
216548_x_at	high-mobility group (nonhistone chromosomal) protein 4-like	HMG4L	0.55284773	1.466979	0.027517515
213029_at	nuclear factor I/B	NFIB	0.552367674	1.46649	0.010192972
36829_at	period homolog 1 (Drosophila)	PER1	0.549943424	1.464028	0.022122228
201249_at	solute carrier family 2 (facilitated glucose transporter), member 1	SLC2A1	0.548916746	1.462987	0.018965453
203188_at	UDP-GlcNAc:betaGal beta-1,3-N-acetylglucosaminyltransferase 1	B3GNT1	0.546597797	1.460637	0.037676207
205251_at	period homolog 2 (Drosophila)	PER2	0.545027181	1.459048	0.046039246
211299_s_at	flotillin 2	FLOT2	0.539367473	1.453335	0.008601198
201037_at	phosphofructokinase, platelet	PFKP	0.538744175	1.452707	0.022459318
219715_s_at	tyrosyl-DNA phosphodiesterase 1	TDP1	0.538275351	1.452235	0.04110309
205996_s_at	adenylate kinase 2	AK2	0.537724224	1.451681	0.03555525
219034_at	poly (ADP-ribose) polymerase family, member 16	PARP16	0.53723599	1.45119	0.009776226
206354_at	solute carrier organic anion transporter family, member 1B1 /// solute carrier organic anion transporter family, member 1B3 /// organic anion transporter LST-3b	LST-3TM12	0.537030462	1.450983	0.006203774
218586_at	chromosome 20 open reading frame 20	C20orf20	0.535580755	1.449526	0.026406296
214437_s_at	serine hydroxymethyltransferase 2 (mitochondrial)	SHMT2	0.534649883	1.448591	0.006293525
40446_at	PHD finger protein 1	PHF1	0.531175185	1.445106	0.033620767
218045_x_at	parathymosin	PTMS	0.530266652	1.444196	0.016520387

206103_at	ras-related C3 botulinum toxin substrate 3 (rho family, small GTP binding protein Rac3)	RAC3	0.529041016	1.44297	0.008191654
214096_s_at	serine hydroxymethyltransferase 2 (mitochondrial)	SHMT2	0.529036773	1.442965	0.012973447
221911_at	ets variant gene 1	ETV1	0.527843459	1.441772	0.044254012
209015_s_at	DnaJ (Hsp40) homolog, subfamily B, member 6	DNAJB6	0.519431388	1.43339	0.03150325
221565_s_at	family with sequence similarity 26, member B	FAM26B	0.518421296	1.432387	0.010853986
203192_at	ATP-binding cassette, sub-family B (MDR/TAP), member 6	ABCB6	0.517412394	1.431386	0.009825362
212277_at	myotubularin related protein 4	MTMR4	0.517190555	1.431166	0.035726339
218788_s_at	SET and MYND domain containing 3	SMYD3	0.516716153	1.430695	0.021540206
218204_s_at	FYVE and coiled-coil domain containing 1	FYCO1	0.514369358	1.42837	0.029174693
206307_s_at	forkhead box D1	FOXD1	0.514260707	1.428262	0.035444819
58780_s_at	hypothetical protein FLJ10357	FLJ10357	0.513692953	1.4277	0.004926805
202499_s_at	solute carrier family 2 (facilitated glucose transporter), member 3	SLC2A3	0.513187625	1.4272	0.044751829
212345_s_at	cAMP responsive element binding protein 3-like 2	CREB3L2	0.511591366	1.425622	0.033620767
219862_s_at	nuclear prelamin A recognition factor	NARF	0.50912829	1.42319	0.003901445
210347_s_at	B-cell CLL/lymphoma 11A (zinc finger protein)	BCL11A	0.506543616	1.420643	0.022760006
91816_f_at	mex-3 homolog D (C. elegans)	MEX3D	0.503570485	1.417718	0.032075577
221567_at	nucleolar protein 3 (apoptosis repressor with CARD domain)	NOL3	0.501272191	1.415461	0.015183691
217872_at	PIH1 domain containing 1	PIH1D1	0.496713043	1.410995	0.015924285
212561_at	RAB6 interacting protein 1	RAB6IP1	0.495179961	1.409497	0.046345183
201043_s_at	acidic (leucine-rich) nuclear phosphoprotein 32 family, member A /// hypothetical protein LOC100128146	ANP32A //.	0.49478163	1.409107	0.015973524
220255_at	Fanconi anemia, complementation group E	FANCE	0.492696659	1.407072	0.013677352
218529_at	CD320 molecule	CD320	0.489511195	1.403969	0.026584734

204766_s_at	nudix (nucleoside diphosphate linked moiety X)-type motif 1	NUDT1	0.488520708	1.403006	0.019624023
57715_at	family with sequence similarity 26, member B	FAM26B	0.488470723	1.402957	0.021384427
203874_s_at	SWI/SNF related, matrix associated, actin dependent regulator of chromatin, subfamily a, member 1	SMARCA1	0.48820374	1.402697	0.011534663
203300_x_at	adaptor-related protein complex 1, sigma 2 subunit	AP1S2	0.487636489	1.402146	0.028076262
59625_at	nucleolar protein 3 (apoptosis repressor with CARD domain)	NOL3	0.48401985	1.398635	0.02901003
220225_at	iroquois homeobox 4	IRX4	0.482496488	1.397159	0.033798226
212922_s_at	SET and MYND domain containing 2	SMYD2	0.482205325	1.396877	0.040078673
203027_s_at	mevalonate (diphospho) decarboxylase	MVD	0.480912176	1.395626	0.035263304
218697_at	NCK interacting protein with SH3 domain	NCKIPSD	0.478358579	1.393158	0.012241734
212414_s_at	septin 6 /// cytokine-like nuclear factor n-pac	N-PAC /// S	0.478009886	1.392821	0.042282472
202304_at	fibronectin type III domain containing 3A	FNDC3A	0.477980853	1.392793	0.023822395
202920_at	ankyrin 2, neuronal	ANK2	0.476512436	1.391376	0.008601198
222129_at	Family with sequence similarity 134, member A	FAM134A	0.475446351	1.390348	0.007610795
207722_s_at	BTB (POZ) domain containing 2	BTBD2	0.475080076	1.389995	0.047825402
396_f_at	erythropoietin receptor	EPOR	0.474487356	1.389424	0.014679603
218988_at	solute carrier family 35, member E3	SLC35E3	0.47356615	1.388538	0.02897476
203492_x_at	centrosomal protein 57kDa	CEP57	0.472478973	1.387492	0.030228794
209680_s_at	kinesin family member C1	KIFC1	0.471784852	1.386824	0.033843707
220725_x_at	Dynein, axonemal, heavy chain 3	DNAH3	0.467596305	1.382804	0.047324525
219264_s_at	protein phosphatase 2 (formerly 2A), regulatory subunit B'', beta /// similar to protein phosphatase 2 (formerly 2A), regulatory subunit B, beta	LOC10013	0.464426439	1.379769	0.029288928
218945_at	chromosome 16 open reading frame 68	C16orf68	0.463567463	1.378947	0.007043637
202735_at	emopamil binding protein (sterol isomerase)	EBP	0.461452735	1.376928	0.027725725

217289_s_at	solute carrier family 37 (glucose-6-phosphate transporter), member 4	SLC37A4	0.460664225	1.376175	0.022593789
207735_at	ring finger protein 125	RNF125	0.460469643	1.37599	0.01814502
202605_at	glucuronidase, beta	GUSB	0.460234356	1.375765	0.01199649
202685_s_at	AXL receptor tyrosine kinase	AXL	0.458812906	1.37441	0.047719364
204638_at	acid phosphatase 5, tartrate resistant	ACP5	0.457477391	1.373139	0.020400033
202459_s_at	lipin 2	LPIN2	0.455404132	1.371167	0.035342149
217952_x_at	PHD finger protein 3	PHF3	0.455301806	1.37107	0.022760006
205127_at	prostaglandin-endoperoxide synthase 1 (prostaglandin G/H synthase and cyclooxygenase)	PTGS1	0.455036917	1.370818	0.04032077
206348_s_at	pyruvate dehydrogenase kinase, isozyme 3	PDK3	0.454347252	1.370163	0.010192972
213787_s_at	emopamil binding protein (sterol isomerase)	EBP	0.453998019	1.369831	0.047825402
212851_at	DCN1, defective in cullin neddylation 1, domain containing 4 (S. cerevisiae)	DCUN1D4	0.453531709	1.369388	0.044254012
202830_s_at	solute carrier family 37 (glucose-6-phosphate transporter), member 4	SLC37A4	0.452885687	1.368775	0.0200001
201234_at	integrin-linked kinase	ILK	0.450945735	1.366936	0.027725725
214978_s_at	protein tyrosine phosphatase, receptor type, f polypeptide (PTPRF), interacting protein (liprin), alpha 4	PPFIA4	0.449579022	1.365642	0.030553407
207265_s_at	KDEL (Lys-Asp-Glu-Leu) endoplasmic reticulum protein retention receptor 3	KDELR3	0.449478435	1.365546	0.037178897
46665_at	sema domain, immunoglobulin domain (Ig), transmembrane domain (TM) and short cytoplasmic domain, (semaphorin) 4C	SEMA4C	0.443555882	1.359952	0.023508348
221016_s_at	transcription factor 7-like 1 (T-cell specific, HMG-box)	TCF7L1	0.443317012	1.359727	0.046352483
206027_at	S100 calcium binding protein A3	S100A3	0.443110396	1.359532	0.038386945
220127_s_at	F-box and leucine-rich repeat protein 12	FBXL12	0.442889194	1.359324	0.006949425
219122_s_at	tRNA-histidine guanylyltransferase 1-like (S. cerevisiae)	THG1L	0.439637957	1.356264	0.01165288

205945_at	interleukin 6 receptor	IL6R	0.438177044	1.354891	0.047324525
210479_s_at	RAR-related orphan receptor A	RORA	0.437188743	1.353963	0.014546584
214442_s_at	protein inhibitor of activated STAT, 2	PIAS2	0.435960762	1.352811	0.041932291
207130_at	zinc finger, MYND-type containing 8	ZMYND8	0.435826943	1.352686	0.011550549
201718_s_at	erythrocyte membrane protein band 4.1-like 2	EPB41L2	0.433098833	1.35013	0.016824371
206857_s_at	FK506 binding protein 1B, 12.6 kDa	FKBP1B	0.431806435	1.348922	0.02144349
211272_s_at	diacylglycerol kinase, alpha 80kDa	DGKA	0.431084432	1.348247	0.041095513
215734_at	chromosome 19 open reading frame 36	C19orf36	0.42923365	1.346518	0.015796973
219655_at	chromosome 7 open reading frame 10	C7orf10	0.428582512	1.345911	0.02597525
209529_at	phosphatidic acid phosphatase type 2C	PPAP2C	0.427461627	1.344865	0.009825362
220036_s_at	limb region 1 homolog (mouse)-like	LMBR1L	0.426903602	1.344345	0.006949425
220081_x_at	hydroxysteroid (17-beta) dehydrogenase 7	HSD17B7	0.426360575	1.343839	0.032430413
219745_at	transmembrane protein 180	TMEM180	0.418680159	1.336704	0.018190072
206746_at	beaded filament structural protein 1, filensin	BFSP1	0.418154256	1.336217	0.005627889
202009_at	twinfilin, actin-binding protein, homolog 2 (Drosophila)	TWF2	0.415046732	1.333342	0.027540443
204416_x_at	apolipoprotein C-I	APOC1	0.412639141	1.331119	0.025799468
220040_x_at	KIAA1166	KIAA1166	0.412195518	1.330709	0.005746175
202982_s_at	acyl-CoA thioesterase 2 /// acyl-CoA thioesterase 1	ACOT1 ///	0.411334565	1.329915	0.015364265
212813_at	junctional adhesion molecule 3 /// hypothetical protein LOC100133502	JAM3 /// L	0.410874704	1.329492	0.02115951
207127_s_at	heterogeneous nuclear ribonucleoprotein H3 (2H9)	HNRNPH3	0.410205036	1.328875	0.032075577
218366_x_at	methyltransferase 11 domain containing 1 /// similar to methyltransferase 11 domain containing 1 isoform 2	LOC73160	0.409485428	1.328212	0.03266687
213471_at	nephronophthisis 4	NPHP4	0.407116327	1.326033	0.021162931
91703_at	EH domain binding protein 1-like 1	EHBP1L1	0.406363261	1.325341	0.025699386
212086_x_at	lamin A/C	LMNA	0.405432514	1.324486	0.049628339

213603_s_at	ras-related C3 botulinum toxin substrate 2 (rho family, small GTP binding protein Rac2)	RAC2	0.4053476	1.324408	0.020677472
202140_s_at	CDC-like kinase 3	CLK3	0.404810217	1.323915	0.010320629
208091_s_at	EGFR-coamplified and overexpressed protein	ECOP	0.403293556	1.322524	0.030228794
220310_at	tubulin, alpha-like 3	TUBAL3	0.40210133	1.321431	0.01055221
202477_s_at	tubulin, gamma complex associated protein 2	TUBGCP2	0.401915334	1.321261	0.012143749
216017_s_at	NGFI-A binding protein 2 (EGR1 binding protein 2)	NAB2	0.399036683	1.318627	0.039944948
211603_s_at	ets variant gene 4 (E1A enhancer binding protein, E1AF)	ETV4	0.394499124	1.314486	0.044831576
206298_at	Rho GTPase activating protein 22	ARHGAP22	0.392928644	1.313056	0.006334784
209862_s_at	centrosomal protein 57kDa	CEP57	0.389598432	1.310029	0.018665191
202587_s_at	adenylate kinase 1	AK1	0.387656355	1.308266	0.013677352
212169_at	FK506 binding protein 9, 63 kDa	FKBP9	0.387418634	1.308051	0.03789462
43427_at	acetyl-Coenzyme A carboxylase beta	ACACB	0.387027059	1.307696	0.022300352
216236_s_at	solute carrier family 2 (facilitated glucose transporter), member 3 /// solute carrier family 2 (facilitated glucose transporter), member 14	SLC2A14 //	0.385610428	1.306412	0.013829495
202030_at	branched chain ketoacid dehydrogenase kinase	BCKDK	0.378903739	1.300353	0.017095271
218225_at	ECSIT homolog (Drosophila)	ECSIT	0.378293407	1.299803	0.027220355
222056_s_at	fumarylacetoacetate hydrolase domain containing 2A	FAHD2A	0.378176978	1.299698	0.030553407
202497_x_at	solute carrier family 2 (facilitated glucose transporter), member 3	SLC2A3	0.377693945	1.299263	0.026683706
203657_s_at	cathepsin F	CTSF	0.375789531	1.297549	0.041932291
221755_at	EH domain binding protein 1-like 1	EHBP1L1	0.373236308	1.295255	0.028076262
219379_x_at	zinc finger protein 358	ZNF358	0.372375972	1.294483	0.025464846
204788_s_at	protoporphyrinogen oxidase	PPOX	0.371418594	1.293624	0.03789462
203875_at	SWI/SNF related, matrix associated, actin dependent regulator of chromatin, subfamily a, member 1	SMARCA1	0.37082982	1.293096	0.036557389

212895_s_at	active BCR-related gene	ABR	0.369254127	1.291685	0.039869083
64440_at	interleukin 17 receptor C	IL17RC	0.365475738	1.288306	0.012241734
205180_s_at	ADAM metallopeptidase domain 8	ADAM8	0.365380277	1.288221	0.009195803
34206_at	centaurin, delta 2	CENTD2	0.358839164	1.282394	0.027540443
209535_s_at			0.358036135	1.28168	0.033997175
202564_x_at	ADP-ribosylation factor-like 2 /// sorting nexin 15	ARL2 /// SN	0.357155122	1.280898	0.02597525
204399_s_at	echinoderm microtubule associated protein like 2	EML2	0.355578565	1.279499	0.024839654
51146_at	phosphatidylinositol glycan anchor biosynthesis, class V	PIGV	0.35503668	1.279018	0.046901507
202718_at	insulin-like growth factor binding protein 2, 36kDa	IGFBP2	0.354438025	1.278487	0.024905824
212817_at	DnaJ (Hsp40) homolog, subfamily B, member 5	DNAJB5	0.353028811	1.277239	0.030545459
219238_at	phosphatidylinositol glycan anchor biosynthesis, class V	PIGV	0.349807807	1.274391	0.017639897
204764_at	farnesyltransferase, CAAX box, beta	FNTB	0.347285202	1.272164	0.015996216
204882_at	Rho GTPase activating protein 25	ARHGAP25	0.342755826	1.268177	0.01579422
202135_s_at	ARP1 actin-related protein 1 homolog B, centractin beta (yeast)	ACTR1B	0.342490776	1.267944	0.049628339
209608_s_at	acetyl-Coenzyme A acetyltransferase 2	ACAT2	0.340782642	1.266443	0.018190072
210874_s_at	N-acetyltransferase 6	NAT6	0.339951012	1.265714	0.009373565
212942_s_at	KIAA1199	KIAA1199	0.337197767	1.2633	0.0102878
215028_at	sema domain, transmembrane domain (TM), and cytoplasmic domain, (semaphorin) 6A	SEMA6A	0.337193729	1.263297	0.048786095
213728_at	lysosomal-associated membrane protein 1	LAMP1	0.336918756	1.263056	0.048210253
207992_s_at	adenosine monophosphate deaminase (isoform E)	AMPD3	0.335046783	1.261418	0.045867156
205854_at	tubby like protein 3	TULP3	0.334288361	1.260755	0.033997175
204773_at	interleukin 11 receptor, alpha	IL11RA	0.333822966	1.260349	0.047363623
221934_s_at	DALR anticodon binding domain containing 3 /// similar to DALR anticodon binding domain containing 3	DALRD3 //,	0.330068947	1.257073	0.048877822
210718_s_at	ADP-ribosylation factor-like 17 pseudogene 1	ARL17P1	0.326856005	1.254277	0.028976133

215294_s_at	SWI/SNF related, matrix associated, actin dependent regulator of chromatin, subfamily a, member 1	SMARCA1	0.32637609	1.25386	0.010872647
218451_at	CUB domain containing protein 1	CDCP1	0.325345027	1.252964	0.036868007
220258_s_at	WD repeat domain 79	WDR79	0.325256555	1.252887	0.034583198
202667_s_at	solute carrier family 39 (zinc transporter), member 7	SLC39A7	0.324479647	1.252213	0.046039246
209615_s_at	p21/Cdc42/Rac1-activated kinase 1 (STE20 homolog, yeast)	PAK1	0.31722668	1.245933	0.024395723
214095_at	serine hydroxymethyltransferase 2 (mitochondrial)	SHMT2	0.317062755	1.245792	0.028652297
201380_at	cartilage associated protein	CRTAP	0.31603727	1.244906	0.048270899
208971_at	uroporphyrinogen decarboxylase	UROD	0.313043058	1.242325	0.042497739
217691_x_at	solute carrier family 16, member 3 (monocarboxylic acid transporter 4)	SLC16A3	0.311784715	1.241242	0.041260074
204918_s_at	myeloid/lymphoid or mixed-lineage leukemia (trithorax homolog, Drosophila); translocated to, 3	MLLT3	0.310736853	1.240341	0.03555525
208250_s_at	deleted in malignant brain tumors 1	DMBT1	0.308658316	1.238555	0.02543191
221939_at	Yip1 domain family, member 2	YIPF2	0.307605011	1.237651	0.045019726
205349_at	guanine nucleotide binding protein (G protein), alpha 15 (Gq class)	GNA15	0.306257368	1.236496	0.026683706
1773_at	farnesyltransferase, CAAX box, beta	FNTB	0.305699114	1.236017	0.015820294
206037_at	cysteine conjugate-beta lyase, cytoplasmic	CCBL1	0.305101295	1.235505	0.025738674
216326_s_at	histone deacetylase 3	HDAC3	0.304592023	1.235069	0.027878759
221544_s_at	mediator complex subunit 16	MED16	0.304414086	1.234917	0.046179549
204543_at	Rap guanine nucleotide exchange factor (GEF) 1	RAPGEF1	0.302272128	1.233085	0.040987387
213553_x_at	apolipoprotein C-I	APOC1	0.301961957	1.23282	0.017601515
220326_s_at	hypothetical protein FLJ10357	FLJ10357	0.298159024	1.229574	0.008880514
203799_at	CD302 molecule	CD302	0.298043331	1.229476	0.026406296
204056_s_at	mevalonate kinase	MVK	0.295002921	1.226887	0.035726339
57532_at	dishevelled, dsh homolog 2 (Drosophila)	DVL2	0.294566108	1.226516	0.009194033

218620_s_at	HemK methyltransferase family member 1	HEMK1	0.29314315	1.225307	0.012522351
218845_at	dual specificity phosphatase 22	DUSP22	0.286954716	1.220062	0.047324525
213789_at	Transcribed locus, moderately similar to NP_006570.1 emopamil binding protein (sterol isomerase) [Homo sapiens]		0.284694335	1.218152	0.047825402
215608_at			0.282365202	1.216187	0.030816874
207035_at	solute carrier family 30 (zinc transporter), member 3	SLC30A3	0.28143763	1.215405	0.038565301
206945_at	lactase	LCT	0.280616536	1.214714	0.030553407
201035_s_at	hydroxyacyl-Coenzyme A dehydrogenase	HADH	0.274870159	1.209885	0.02897476
206043_s_at	ATPase, Ca++ transporting, type 2C, member 2	ATP2C2	0.273248013	1.208526	0.042105187
206980_s_at	fms-related tyrosine kinase 3 ligand	FLT3LG	0.270442437	1.206178	0.042957853
217310_s_at	forkhead box J3	FOXJ3	0.263272062	1.200198	0.022219699
37425_g_at	coiled-coil alpha-helical rod protein 1	CCHCR1	0.260971504	1.198285	0.033997465
207727_s_at	mutY homolog (E. coli)	MUTYH	0.259452184	1.197024	0.036846349
219680_at	NLR family member X1	NLRX1	0.258018939	1.195835	0.016199453
217395_at	metallothionein 4	MT4	0.257283142	1.195226	0.04353849
202926_at	neuroblastoma-amplified protein	NAG	0.257209488	1.195165	0.046356918
218818_at	four and a half LIM domains 3	FHL3	0.2561677	1.194302	0.03383209
215855_s_at	TATA element modulatory factor 1	TMF1	0.25194571	1.190812	0.047782169
211430_s_at	immunoglobulin heavy locus /// immunoglobulin heavy constant gamma 1 (G1m marker) /// immunoglobulin heavy constant gamma 2 (G2m marker) /// immunoglobulin heavy constant mu /// immunoglobulin heavy variable 4-31	IGH@ /// I	0.251083532	1.190101	0.043327649
218636_s_at	mannosidase, alpha, class 1B, member 1	MAN1B1	0.249096819	1.188463	0.048877822
207709_at	protein kinase, AMP-activated, alpha 2 catalytic subunit	PRKAA2	0.247863387	1.187447	0.0200001
202986_at	aryl-hydrocarbon receptor nuclear translocator 2	ARNT2	0.245554615	1.185548	0.023727684

204294_at	aminomethyltransferase	AMT	0.240971263	1.181788	0.018965453
215907_at	CDNA: FLJ23540 fis, clone LNG08239		0.240805308	1.181652	0.045510962
210185_at	calcium channel, voltage-dependent, beta 1 subunit	CACNB1	0.236650544	1.178254	0.049444401
211554_s_at	apoptotic peptidase activating factor 1	APAF1	0.23658016	1.178196	0.038565301
215700_x_at	copine VI (neuronal)	CPNE6	0.235167331	1.177043	0.036557389
204392_at	calcium/calmodulin-dependent protein kinase I	CAMK1	0.233778462	1.175911	0.041325032
213264_at	poly(rC) binding protein 2	PCBP2	0.233348405	1.17556	0.040987387
219516_at	transient receptor potential cation channel, subfamily V, member 4	TRPV4	0.232587408	1.17494	0.034616092
218154_at	gasdermin domain containing 1	GSDMDC1	0.226656798	1.17012	0.035263304
47571_at	zinc finger protein 236	ZNF236	0.214145842	1.160017	0.044344292
219371_s_at	Kruppel-like factor 2 (lung)	KLF2	0.212252517	1.158496	0.041621834
213675_at	CDNA FLJ25106 fis, clone CBR01467		0.211293245	1.157726	0.030553407
211731_x_at	synovial sarcoma, X breakpoint 3	SSX3	0.210576241	1.15715	0.036520037
219019_at	leucine-rich repeats and death domain containing	LRDD	0.20930005	1.156127	0.037676207
207227_x_at	ret finger protein-like 2	RFPL2	0.20839363	1.155401	0.037963867
217495_x_at	calcitonin-related polypeptide alpha	CALCA	0.207753511	1.154888	0.045525685
206520_x_at	sialic acid binding Ig-like lectin 6	SIGLEC6	0.204397442	1.152205	0.037318715
205359_at	A kinase (PRKA) anchor protein 6	AKAP6	0.202665104	1.150822	0.041932291
210586_x_at	Rh blood group, D antigen	RHD	0.202654979	1.150814	0.045301166
215594_at	PTR2 mRNA for repetitive sequence		0.193369378	1.143431	0.049017911
216704_at	Transcribed locus		0.191787404	1.142178	0.048505724
220008_at	NKF3 kinase family member	SGK269	0.187786834	1.139015	0.039856933
214957_at	actin-like 8	ACTL8	-0.19936894	-1.1482	0.039944948
213196_at	zinc finger protein 629	ZNF629	-0.21420606	-1.16007	0.034446605
221088_s_at	protein phosphatase 1, regulatory (inhibitor) subunit 9A	PPP1R9A	-0.21616243	-1.16164	0.045728765
217749_at	coatomer protein complex, subunit gamma	COPG	-0.23826968	-1.17958	0.02297483
210790_s_at	SAR1 gene homolog A (S. cerevisiae)	SAR1A	-0.24414957	-1.18439	0.026683706
218122_s_at	SUMO1/sentrin/SMT3 specific peptidase 2	SENP2	-0.24472679	-1.18487	0.029594023

212576_at	mahogunin, ring finger 1	MGRN1	-0.24676287	-1.18654	0.046643175
206175_x_at	zinc finger protein 222	ZNF222	-0.24999702	-1.1892	0.025799468
210112_at	Hermansky-Pudlak syndrome 1	HPS1	-0.2516159	-1.19054	0.038057519
221951_at	transmembrane protein 80	TMEM80	-0.25426236	-1.19273	0.039245729
63825_at	abhydrolase domain containing 2	ABHD2	-0.2570825	-1.19506	0.020400033
213303_x_at	zinc finger and BTB domain containing 7A	ZBTB7A	-0.25825653	-1.19603	0.046269754
212747_at	ankyrin repeat and sterile alpha motif domain containing 1A	ANKS1A	-0.25866066	-1.19637	0.02012995
215130_s_at	IQ motif containing K	IQCK	-0.26117092	-1.19845	0.03669617
218869_at	malonyl-CoA decarboxylase	MLYCD	-0.261792	-1.19897	0.034308379
209395_at	chitinase 3-like 1 (cartilage glycoprotein-39)	CHI3L1	-0.26289612	-1.19988	0.016606812
204843_s_at	protein kinase, cAMP-dependent, regulatory, type II, alpha	PRKAR2A	-0.26426349	-1.20102	0.020112851
219128_at	chromosome 2 open reading frame 42	C2orf42	-0.2650744	-1.2017	0.038334795
209818_s_at	hyaluronan binding protein 4	HABP4	-0.26865401	-1.20468	0.010853986
205266_at	leukemia inhibitory factor (cholinergic differentiation factor)	LIF	-0.26978809	-1.20563	0.023727684
209992_at	6-phosphofructo-2-kinase/fructose-2,6-biphosphatase 2	PFKFB2	-0.2719328	-1.20742	0.045424984
215150_at	YOD1 OTU deubiquinating enzyme 1 homolog (S. cerevisiae)	YOD1	-0.27379049	-1.20898	0.030495392
210188_at	GA binding protein transcription factor, alpha subunit 60kDa	GABPA	-0.27493628	-1.20994	0.047011358
202288_at	FK506 binding protein 12-rapamycin associated protein 1	FRAP1	-0.28094519	-1.21499	0.020891228
44696_at	TBC1 domain family, member 13	TBC1D13	-0.28205895	-1.21593	0.025338267
212848_s_at	chromosome 9 open reading frame 3	C9orf3	-0.28457753	-1.21805	0.045019726
218329_at	PR domain containing 4	PRDM4	-0.2863203	-1.21953	0.047782169
208184_s_at	transmembrane protein 1	TMEM1	-0.28651149	-1.21969	0.03669617
204350_s_at	mediator complex subunit 7	MED7	-0.28722105	-1.22029	0.030883173
221780_s_at	DEAD (Asp-Glu-Ala-Asp) box polypeptide 27	DDX27	-0.2876454	-1.22065	0.043867818

210573_s_at	polymerase (RNA) III (DNA directed) polypeptide C (62kD)	POLR3C	-0.28995188	-1.2226	0.047825402
220444_at	zinc finger protein 557	ZNF557	-0.29164872	-1.22404	0.029409314
214077_x_at	Meis homeobox 3 pseudogene 1	MEIS3P1	-0.2936682	-1.22575	0.04847513
50374_at	chromosome 17 open reading frame 90	C17orf90	-0.29397963	-1.22602	0.037676207
219617_at	chromosome 2 open reading frame 34	C2orf34	-0.2945507	-1.2265	0.015830371
217930_s_at	toll interacting protein	TOLLIP	-0.29504029	-1.22692	0.030553407
201830_s_at	neuroepithelial cell transforming gene 1	NET1	-0.29573555	-1.22751	0.037676207
221002_s_at	tetraspanin 14	TSPAN14	-0.29734387	-1.22888	0.03669617
221799_at	chondroitin sulfate glucuronyltransferase	CSGlcA-T	-0.29860816	-1.22996	0.045899526
208952_s_at	La ribonucleoprotein domain family, member 5	LARP5	-0.30368797	-1.2343	0.043045162
216400_at	glucosidase, beta; acid (includes glucosylceramidase) /// glucosidase, beta; acid, pseudogene	GBA /// GB	-0.30462259	-1.2351	0.029594023
201686_x_at	apoptosis inhibitor 5	API5	-0.30638807	-1.23661	0.030553407
219406_at	chromosome 1 open reading frame 50	C1orf50	-0.30710928	-1.23723	0.048110721
203659_s_at	tripartite motif-containing 13	TRIM13	-0.30764929	-1.23769	0.009110746
202486_at	AFG3 ATPase family gene 3-like 2 (yeast)	AFG3L2	-0.3086825	-1.23858	0.016278635
212837_at	KIAA0157	KIAA0157	-0.31217164	-1.24158	0.047363623
205995_x_at	IQ motif containing B1	IQCB1	-0.31237	-1.24175	0.024778461
218766_s_at	tryptophanyl tRNA synthetase 2, mitochondrial	WARS2	-0.313028	-1.24231	0.022300352
218587_s_at	KTEL (Lys-Tyr-Glu-Leu) containing 1	KTELC1	-0.31797937	-1.24658	0.033997175
218596_at	TBC1 domain family, member 13	TBC1D13	-0.31806143	-1.24665	0.031032444
203682_s_at	isovaleryl Coenzyme A dehydrogenase	IVD	-0.31938487	-1.2478	0.047692556
218113_at	transmembrane protein 2	TMEM2	-0.32006757	-1.24839	0.031921312
202831_at	glutathione peroxidase 2 (gastrointestinal)	GPX2	-0.32121418	-1.24938	0.039944948
209912_s_at	KIAA0415	KIAA0415	-0.32131329	-1.24947	0.036868007
212197_x_at	myosin phosphatase-Rho interacting protein	M-RIP	-0.32149639	-1.24963	0.030304577
214741_at	zinc finger protein 131	ZNF131	-0.32199987	-1.25006	0.019500168

213462_at	neuronal PAS domain protein 2	NPAS2	-0.32233276	-1.25035	0.009849164
208523_x_at	histone cluster 1, H2bg /// histone cluster 1, H2bf /// histone cluster 1, H2be /// histone cluster 1, H2bi /// histone cluster 1, H2bc	HIST1H2BC	-0.32272137	-1.25069	0.04032077
221745_at	WD repeat domain 68	WDR68	-0.32334566	-1.25123	0.046352483
218682_s_at	solute carrier family 4 (anion exchanger), member 1, adaptor protein	SLC4A1AP	-0.32565857	-1.25324	0.033442044
205131_x_at	C-type lectin domain family 11, member A	CLEC11A	-0.32779409	-1.25509	0.008488821
212474_at	KIAA0241	KIAA0241	-0.32872866	-1.25591	0.041548563
221877_at	CDNA FLJ38849 fis, clone MESAN2008936		-0.3315332	-1.25835	0.044000439
213126_at	mediator complex subunit 8	MED8	-0.33168873	-1.25849	0.035268202
204605_at	cell growth regulator with ring finger domain 1	CGRRF1	-0.33207881	-1.25883	0.03669617
210461_s_at	actin binding LIM protein 1	ABLIM1	-0.33245956	-1.25916	0.009825362
202851_at	hypothetical protein FLJ11506	FLJ11506	-0.33271971	-1.25939	0.018190072
211004_s_at	aldehyde dehydrogenase 3 family, member B1	ALDH3B1	-0.33371154	-1.26025	0.044259029
37943_at	zinc finger, FYVE domain containing 26	ZFYVE26	-0.33380359	-1.26033	0.022122228
212878_s_at	kinesin light chain 1	KLC1	-0.33416373	-1.26065	0.026406296
212834_at	DEAD (Asp-Glu-Ala-Asp) box polypeptide 52	DDX52	-0.33425674	-1.26073	0.031921312
213392_at	IQ motif containing K	IQCK	-0.334467	-1.26091	0.008191654
219710_at	SH3 domain and tetratricopeptide repeats 2	SH3TC2	-0.33625729	-1.26248	0.031851146
205873_at	phosphatidylinositol glycan anchor biosynthesis, class L	PIGL	-0.3369067	-1.26305	0.020331276
204872_at	transducin-like enhancer of split 4 (E(sp1) homolog, Drosophila)	TLE4	-0.33878936	-1.26469	0.012241734
212260_at	GRB10 interacting GYF protein 2	GIGYF2	-0.34034263	-1.26606	0.045125221
219603_s_at	zinc finger protein 226	ZNF226	-0.34244163	-1.2679	0.03394891
213184_at	SUMO1/sentrin specific peptidase 5	SENP5	-0.34278842	-1.26821	0.042570882
202908_at	Wolfram syndrome 1 (wolframin)	WFS1	-0.34376435	-1.26906	0.045931814
205322_s_at	metal-regulatory transcription factor 1	MTF1	-0.34595357	-1.27099	0.040948632

212290_at	solute carrier family 7 (cationic amino acid transporter, y+ system), member 1	SLC7A1	-0.34769555	-1.27253	0.042323058
213090_s_at	TAF4 RNA polymerase II, TATA box binding protein (TBP)-associated factor, 135kDa	TAF4	-0.34959901	-1.27421	0.047064544
219675_s_at	UDP-glucuronate decarboxylase 1	UXS1	-0.350009	-1.27457	0.049639322
221946_at	chromosome 9 open reading frame 116	C9orf116	-0.35023712	-1.27477	0.02012995
220642_x_at	G protein-coupled receptor 89B /// G protein-coupled receptor 89A /// G protein-coupled receptor 89C	GPR89A //,	-0.35164037	-1.27601	0.03376599
212497_at	mitogen-activated protein kinase 1 interacting protein 1-like	MAPK1IP1I	-0.35247668	-1.27675	0.025699386
202818_s_at	transcription elongation factor B (SIII), polypeptide 3 (110kDa, elongin A)	TCEB3	-0.35298667	-1.2772	0.033997175
212325_at	LIM and calponin homology domains 1	LIMCH1	-0.35299039	-1.27721	0.035341012
212896_at	superkiller viralicidic activity 2-like 2 (S. cerevisiae)	SKIV2L2	-0.35427138	-1.27834	0.041789977
210048_at	N-ethylmaleimide-sensitive factor attachment protein, gamma	NAPG	-0.35524977	-1.27921	0.023632473
57539_at	zinc finger, CCCH-type with G patch domain	ZGPAT	-0.35719089	-1.28093	0.040856338
202108_at	peptidase D	PEPD	-0.35734753	-1.28107	0.03555525
217926_at	chromosome 19 open reading frame 53	C19orf53	-0.3588749	-1.28243	0.04032077
203456_at	PRA1 domain family, member 2	PRAF2	-0.35945292	-1.28294	0.039944948
213889_at	phosphatidylinositol glycan anchor biosynthesis, class L	PIGL	-0.36051898	-1.28389	0.045510962
209895_at	protein tyrosine phosphatase, non-receptor type 11 (Noonan syndrome 1)	PTPN11	-0.36327242	-1.28634	0.012714488
201512_s_at	translocase of outer mitochondrial membrane 70 homolog A (S. cerevisiae)	TOMM70A	-0.36434895	-1.2873	0.046356918

209799_at	protein kinase, AMP-activated, alpha 1 catalytic subunit	PRKAA1	-0.36459398	-1.28752	0.04220401
221059_s_at	coactosin-like 1 (Dictyostelium)	COTL1	-0.36461809	-1.28754	0.048210253
203630_s_at	component of oligomeric golgi complex 5	COG5	-0.36771676	-1.29031	0.033837739
59437_at	chromosome 9 open reading frame 116	C9orf116	-0.3683767	-1.2909	0.039419532
204210_s_at	phosphate cytidylyltransferase 1, choline, alpha	PCYT1A	-0.36851943	-1.29103	0.038652244
213111_at	phosphatidylinositol-3-phosphate/phosphatidylinositol 5-kinase, type III	PIP5K3	-0.36918585	-1.29162	0.03394891
201174_s_at	telomeric repeat binding factor 2, interacting protein	TERF2IP	-0.36946926	-1.29188	0.033997175
205246_at	peroxisome biogenesis factor 13	PEX13	-0.36991992	-1.29228	0.030703647
205280_at	glycine receptor, beta	GLRB	-0.37102159	-1.29327	0.020225356
212034_s_at	exocyst complex component 7	EXOC7	-0.37166948	-1.29385	0.021969948
218314_s_at	chromosome 11 open reading frame 57	C11orf57	-0.37209663	-1.29423	0.030686502
204590_x_at	vacuolar protein sorting 33 homolog A (S. cerevisiae)	VPS33A	-0.37338263	-1.29539	0.022122228
214869_x_at	GTPase activating protein and VPS9 domains 1	GAPVD1	-0.37420031	-1.29612	0.037676207
203039_s_at	NADH dehydrogenase (ubiquinone) Fe-S protein 1, 75kDa (NADH-coenzyme Q reductase)	NDUFS1	-0.37476914	-1.29663	0.036078545
204071_s_at	topoisomerase I binding, arginine/serine-rich	TOPORS	-0.37558678	-1.29737	0.026683706
213311_s_at	transcription factor 25 (basic helix-loop-helix)	TCF25	-0.37580624	-1.29756	0.035341012
87100_at	abhydrolase domain containing 2	ABHD2	-0.37617683	-1.2979	0.0200001
219244_s_at	mitochondrial ribosomal protein L46	MRPL46	-0.37767797	-1.29925	0.037739164
214527_s_at	polyglutamine binding protein 1	PQBP1	-0.37783718	-1.29939	0.041519922
212731_at	ankyrin repeat domain 46	ANKRD46	-0.37823845	-1.29975	0.012476914
209268_at	vacuolar protein sorting 45 homolog (S. cerevisiae)	VPS45	-0.3792763	-1.30069	0.030675364

217681_at	wingless-type MMTV integration site family, member 7B	WNT7B	-0.38060425	-1.30189	0.026683706
209445_x_at	chromosome 7 open reading frame 44	C7orf44	-0.3808174	-1.30208	0.039353364
214946_x_at	family with sequence similarity 21, member B /// family with sequence similarity 21, member C /// family with sequence similarity 21, member A /// family with sequence similarity 21, member D	FAM21A //	-0.38260266	-1.30369	0.034142802
212216_at	prolyl endopeptidase-like	PREPL	-0.38382369	-1.3048	0.035341012
1487_at	estrogen-related receptor alpha	ESRRA	-0.38386404	-1.30483	0.034029487
212211_at	ankyrin repeat domain 17	ANKRD17	-0.3845323	-1.30544	0.033997175
219922_s_at	latent transforming growth factor beta binding protein 3	LTBP3	-0.38488174	-1.30575	0.040856338
201836_s_at	suppressor of Ty 7 (S. cerevisiae)-like	SUPT7L	-0.38642165	-1.30715	0.028976133
203212_s_at	myotubularin related protein 2	MTMR2	-0.38676602	-1.30746	0.006921301
203579_s_at	solute carrier family 7 (cationic amino acid transporter, y+ system), member 6 /// transient receptor potential cation channel, subfamily V, member 6	SLC7A6 ///	-0.38686779	-1.30755	0.029644613
202843_at	DnaJ (Hsp40) homolog, subfamily B, member 9	DNAJB9	-0.38907619	-1.30955	0.032128563
204075_s_at	KIAA0562	KIAA0562	-0.38976025	-1.31018	0.030553407
221765_at	UDP-glucose ceramide glucosyltransferase	UGCG	-0.39145228	-1.31171	0.017353002
204630_s_at	golgi SNAP receptor complex member 1	GOSR1	-0.39324308	-1.31334	0.028368833
218928_s_at	solute carrier family 37 (glycerol-3-phosphate transporter), member 1	SLC37A1	-0.39401195	-1.31404	0.028694993
209064_x_at	poly(A) binding protein interacting protein 1	PAIP1	-0.39457141	-1.31455	0.045867156
214791_at	hypothetical protein BC004921	LOC93349	-0.39479896	-1.31476	0.027725725
209412_at	transmembrane protein 1	TMEM1	-0.39635524	-1.31618	0.021311374
218285_s_at	3-hydroxybutyrate dehydrogenase, type 2	BDH2	-0.3969674	-1.31674	0.048951461

221541_at	cysteine-rich secretory protein LCCL domain containing 2	CRISPLD2	-0.39780021	-1.3175	0.009849164
221848_at	zinc finger, CCCH-type with G patch domain	ZGPAT	-0.39802983	-1.31771	0.01671059
203592_s_at	follistatin-like 3 (secreted glycoprotein)	FSTL3	-0.39915566	-1.31874	0.030553407
207510_at	bradykinin receptor B1	BDKRB1	-0.39915631	-1.31874	0.039384241
205541_s_at	G1 to S phase transition 2	GSPT2	-0.39927802	-1.31885	0.029644613
201712_s_at	RAN binding protein 2	RANBP2	-0.40121481	-1.32062	0.031124797
74694_s_at	rabaptin, RAB GTPase binding effector protein 2 /// similar to RABEP2 protein	LOC10013:	-0.40139185	-1.32078	0.030816874
206764_x_at	metallophosphoesterase 1	MPPE1	-0.40239039	-1.3217	0.017771058
210676_x_at	RANBP2-like and GRIP domain containing 5 /// RANBP2-like and GRIP domain containing 8 /// RANBP2-like and GRIP domain containing 6	RGPD5 ///	-0.40263532	-1.32192	0.04084065
221895_at	motile sperm domain containing 2	MOSPD2	-0.40292436	-1.32219	0.016520387
221884_at	ecotropic viral integration site 1	EVI1	-0.40346235	-1.32268	0.047706878
39549_at	neuronal PAS domain protein 2	NPAS2	-0.4037616	-1.32295	0.009110746
222304_x_at	olfactory receptor, family 7, subfamily E, member 47 pseudogene	OR7E47P	-0.4046627	-1.32378	0.01323345
203487_s_at	armadillo repeat containing 8	ARMC8	-0.40602138	-1.32503	0.026683706
205210_at	transforming growth factor, beta receptor associated protein 1	TGFBRAP1	-0.40683667	-1.32578	0.012932821
202453_s_at	general transcription factor IIH, polypeptide 1, 62kDa	GTF2H1	-0.40692095	-1.32585	0.030553407
203115_at	ferrochelatase (protoporphyria)	FECH	-0.40821618	-1.32704	0.010739422
218318_s_at	nemo-like kinase	NLK	-0.40823074	-1.32706	0.030051009
210085_s_at	annexin A9	ANXA9	-0.4086447	-1.32744	0.013533787
203748_x_at	RNA binding motif, single stranded interacting protein 1	RBMS1	-0.40893449	-1.3277	0.045983569
218763_at	syntaxin 18	STX18	-0.40928378	-1.32803	0.011347725
218749_s_at	solute carrier family 24 (sodium/potassium/calcium exchanger), member 6	SLC24A6	-0.41016751	-1.32884	0.026584734
205279_s_at	glycine receptor, beta	GLRB	-0.4113859	-1.32996	0.014546584

209755_at	nicotinamide nucleotide adenylyltransferase 2 /// hypothetical protein LOC100131795	LOC10013[illegible]	-0.41198589	-1.33052	0.022122228
215285_s_at	putative homeodomain transcription factor 1	PHTF1	-0.41294406	-1.3314	0.007131295
201837_s_at	suppressor of Ty 7 (S. cerevisiae)-like	SUPT7L	-0.41367575	-1.33208	0.028652297
217981_s_at	fracture callus 1 homolog (rat)	FXC1	-0.41403996	-1.33241	0.039768489
202744_at	solute carrier family 20 (phosphate transporter), member 2	SLC20A2	-0.41524332	-1.33352	0.033997175
200629_at	tryptophanyl-tRNA synthetase	WARS	-0.41611049	-1.33433	0.017541446
204231_s_at	fatty acid amide hydrolase	FAAH	-0.41643117	-1.33462	0.045141397
211285_s_at	ubiquitin protein ligase E3A (human papilloma virus E6-associated protein, Angelman syndrome)	UBE3A	-0.41679581	-1.33496	0.027517515
218672_at	sodium channel modifier 1	SCNM1	-0.41844054	-1.33648	0.020891228
209394_at	acetylserotonin O-methyltransferase-like	ASMTL	-0.41860357	-1.33663	0.006921301
203128_at	serine palmitoyltransferase, long chain base subunit 2	SPTLC2	-0.41867544	-1.3367	0.007610795
217811_at	selenoprotein T	SELT	-0.41869689	-1.33672	0.047825402
218219_s_at	LanC lantibiotic synthetase component C-like 2 (bacterial)	LANCL2	-0.41936588	-1.33734	0.037735868
209533_s_at	phospholipase A2-activating protein	PLAA	-0.41944544	-1.33741	0.030767069
60471_at	Ras and Rab interactor 3	RIN3	-0.42025452	-1.33816	0.00601739
219229_at	solute carrier organic anion transporter family, member 3A1	SLCO3A1	-0.42073504	-1.33861	0.035134898
201521_s_at	nuclear cap binding protein subunit 2, 20kDa	NCBP2	-0.42205416	-1.33983	0.022794017
57540_at	ribokinase	RBKS	-0.42264431	-1.34038	0.028884791
202578_s_at	DEAD (Asp-Glu-Ala-As) box polypeptide 19A	DDX19A	-0.423098	-1.3408	0.041052549
216958_s_at	isovaleryl Coenzyme A dehydrogenase	IVD	-0.42359937	-1.34127	0.020059581
64488_at	CDNA FLJ38849 fis, clone MESAN2008936		-0.42361903	-1.34129	0.016278635
208953_at	La ribonucleoprotein domain family, member 5	LARP5	-0.42367344	-1.34134	0.020400033
203245_s_at	FLJ35348	FLJ35348	-0.42398049	-1.34162	0.012571055

220172_at	chromosome 2 open reading frame 37	C2orf37	-0.42402698	-1.34167	0.022122228
200604_s_at	protein kinase, cAMP-dependent, regulatory, type I, alpha (tissue specific extinguisher 1)	PRKAR1A	-0.42574805	-1.34327	0.026406296
200609_s_at	WD repeat domain 1	WDR1	-0.42579837	-1.34332	0.048665054
209232_s_at	dynactin 5 (p25)	DCTN5	-0.42598522	-1.34349	0.008191654
204047_s_at	phosphatase and actin regulator 2	PHACTR2	-0.42604868	-1.34355	0.033843707
217185_s_at	zinc finger protein 259 /// similar to zinc finger protein 259	LOC44224(	-0.4263799	-1.34386	0.039353364
209234_at	kinesin family member 1B	KIF1B	-0.42679604	-1.34424	0.02449946
218343_s_at	general transcription factor IIIC, polypeptide 3, 102kDa	GTF3C3	-0.42744879	-1.34485	0.01672165
203427_at	ASF1 anti-silencing function 1 homolog A (S. cerevisiae)	ASF1A	-0.42779332	-1.34517	0.031415364
211801_x_at	mitofusin 1	MFN1	-0.42861631	-1.34594	0.041260074
213127_s_at	mediator complex subunit 8	MED8	-0.42870661	-1.34603	0.016278635
45526_g_at	hypothetical protein FLJ14154	FLJ14154	-0.42944952	-1.34672	0.013677352
202297_s_at	RER1 retention in endoplasmic reticulum 1 homolog (S. cerevisiae)	RER1	-0.43013604	-1.34736	0.031124797
208580_x_at		Multiple G(	-0.43056028	-1.34776	0.028805485
212312_at	BCL2-like 1	BCL2L1	-0.43265477	-1.34971	0.031236577
218344_s_at	REST corepressor 3	RCOR3	-0.433523	-1.35053	0.015020188
211709_s_at	C-type lectin domain family 11, member A	CLEC11A	-0.43401592	-1.35099	0.01323345
217935_s_at	ubiquinol-cytochrome c reductase complex chaperone, CBP3 homolog (yeast)	UQCC	-0.43488351	-1.3518	0.026683706
213140_s_at	synovial sarcoma translocation gene on chromosome 18-like 1	SS18L1	-0.43497845	-1.35189	0.028076262
212802_s_at	GTPase activating protein and VPS9 domains 1	GAPVD1	-0.43551217	-1.35239	0.039944948
215075_s_at	growth factor receptor-bound protein 2	GRB2	-0.43613524	-1.35298	0.020677472
208490_x_at	histone cluster 1, H2bg /// histone cluster 1, H2bf /// histone cluster 1, H2be /// histone cluster 1, H2bi /// histone cluster 1, H2bc	HIST1H2BC	-0.43668012	-1.35349	0.037044614
216698_x_at		LOC44145:	-0.43682244	-1.35362	0.013682976

221815_at	abhydrolase domain containing 2	ABHD2	-0.43794135	-1.35467	0.025490308
218295_s_at	nucleoporin 50kDa	NUP50	-0.43833996	-1.35504	0.014546584
218612_s_at	tumor suppressing subtransferable candidate 4	TSSC4	-0.43933664	-1.35598	0.033620767
217150_s_at	neurofibromin 2 (merlin)	NF2	-0.43940806	-1.35605	0.040948632
221513_s_at	UTP14, U3 small nucleolar ribonucleoprotein, homolog C (yeast) /// UTP14, U3 small nucleolar ribonucleoprotein, homolog A (yeast)	UTP14A //,	-0.44189217	-1.35838	0.030835939
212370_x_at	family with sequence similarity 21, member B /// family with sequence similarity 21, member A	FAM21A //	-0.4425154	-1.35897	0.020059581
203767_s_at	steroid sulfatase (microsomal), isozyme S	STS	-0.44284946	-1.35929	0.023378717
201583_s_at	Sec23 homolog B (S. cerevisiae)	SEC23B	-0.44357429	-1.35997	0.006921301
218527_at	aprataxin	APTX	-0.44365592	-1.36005	0.025699386
218283_at	synovial sarcoma translocation gene on chromosome 18-like 2	SS18L2	-0.44405529	-1.36042	0.035834164
210653_s_at	branched chain keto acid dehydrogenase E1, beta polypeptide (maple syrup urine disease)	BCKDHB	-0.44446936	-1.36081	0.035264267
203616_at	polymerase (DNA directed), beta	POLB	-0.44564884	-1.36193	0.039944948
212658_at	lipoma HMGIC fusion partner-like 2	LHFPL2	-0.44598955	-1.36225	0.019886651
213315_x_at	chromosome X open reading frame 40A	CXorf40A	-0.44626231	-1.36251	0.029066198
219395_at	RNA binding motif protein 35B	RBM35B	-0.44669792	-1.36292	0.016278635
202347_s_at	ubiquitin-conjugating enzyme E2K (UBC1 homolog, yeast)	UBE2K	-0.44727988	-1.36347	0.035726339
212961_x_at	chromosome X open reading frame 40A /// chromosome X open reading frame 40B	CXorf40A /	-0.44750831	-1.36368	0.022673136
218837_s_at	ubiquitin-conjugating enzyme E2D 4 (putative)	UBE2D4	-0.44779065	-1.36395	0.015830371
218333_at	Der1-like domain family, member 2	DERL2	-0.44811102	-1.36425	0.037676207
204495_s_at	chromosome 15 open reading frame 39	C15orf39	-0.44825603	-1.36439	0.02901003

201632_at	eukaryotic translation initiation factor 2B, subunit 1 alpha, 26kDa	EIF2B1	-0.44865143	-1.36476	0.019258724
222140_s_at	G protein-coupled receptor 89B /// G protein-coupled receptor 89A /// G protein-coupled receptor 89C	GPR89A //,	-0.44871091	-1.36482	0.037178897
202680_at	general transcription factor IIE, polypeptide 2, beta 34kDa	GTF2E2	-0.44907056	-1.36516	0.02325585
221543_s_at	ER lipid raft associated 2	ERLIN2	-0.44984849	-1.3659	0.034485301
221847_at	hypothetical protein LOC100129361	LOC10012[illegible]	-0.45100496	-1.36699	0.030553407
214855_s_at	GTPase activating Rap/RanGAP domain-like 1	GARNL1	-0.45132297	-1.36729	0.022300352
202271_at	F-box protein 28	FBXO28	-0.45159189	-1.36755	0.028368833
218304_s_at	oxysterol binding protein-like 11	OSBPL11	-0.45218842	-1.36811	0.022300352
218479_s_at	exportin 4	XPO4	-0.45281744	-1.36871	0.017583366
203116_s_at	ferrochelatase (protoporphyria)	FECH	-0.45318098	-1.36906	0.010853986
208822_s_at	death associated protein 3	DAP3	-0.45364469	-1.3695	0.030534502
203166_at	craniofacial development protein 1	CFDP1	-0.45366377	-1.36951	0.006949425
205588_s_at	FGFR1 oncogene partner	FGFR1OP	-0.45389879	-1.36974	0.049639322
203517_at	metaxin 2	MTX2	-0.45523462	-1.37101	0.035238023
202165_at	protein phosphatase 1, regulatory (inhibitor) subunit 2	PPP1R2	-0.45592796	-1.37166	0.017797984
200596_s_at	eukaryotic translation initiation factor 3, subunit A	EIF3A	-0.45679046	-1.37249	0.015628504
204374_s_at	galactokinase 1	GALK1	-0.45757518	-1.37323	0.00887733
222116_s_at	TBC1 domain family, member 16	TBC1D16	-0.45802708	-1.37366	0.020331276
205285_s_at	FYN binding protein (FYB-120/130)	FYB	-0.45827374	-1.3739	0.022219699
219343_at	cell division cycle 37 homolog (S. cerevisiae)-like 1	CDC37L1	-0.45918799	-1.37477	0.017353002
218375_at	nudix (nucleoside diphosphate linked moiety X)-type motif 9	NUDT9	-0.4598952	-1.37544	0.02597525
221503_s_at	karyopherin alpha 3 (importin alpha 4)	KPNA3	-0.45990592	-1.37545	0.033843707
203556_at	zinc fingers and homeoboxes 2	ZHX2	-0.4617977	-1.37726	0.015183691

213877_x_at	transcription elongation factor B (SIII), polypeptide 2 (18kDa, elongin B)	TCEB2	-0.46382922	-1.3792	0.013044506
201854_s_at	ATM interactor	ATMIN	-0.46398511	-1.37935	0.016824371
213021_at	golgi SNAP receptor complex member 1	GOSR1	-0.46404634	-1.37941	0.012749147
36554_at	acetylserotonin O-methyltransferase-like	ASMTL	-0.46412393	-1.37948	0.025501804
219496_at	ankyrin repeat domain 57	ANKRD57	-0.46429119	-1.37964	0.020112851
212666_at	SMAD specific E3 ubiquitin protein ligase 1	SMURF1	-0.46442117	-1.37976	0.045983569
214545_s_at	proline synthetase co-transcribed homolog (bacterial)	PROSC	-0.4667049	-1.38195	0.016278635
217971_at	mitogen-activated protein kinase kinase 1 interacting protein 1	MAP2K1IP1	-0.46676882	-1.38201	0.040987387
209922_at	BRCA1 associated protein	BRAP	-0.46718582	-1.38241	0.013358527
210802_s_at	DIM1 dimethyladenosine transferase 1-like (S. cerevisiae)	DIMT1L	-0.46763999	-1.38285	0.034249673
219821_s_at	glucose-fructose oxidoreductase domain containing 1	GFOD1	-0.46817039	-1.38335	0.009110746
203578_s_at	solute carrier family 7 (cationic amino acid transporter, y+ system), member 6 /// transient receptor potential cation channel, subfamily V, member 6	SLC7A6 ///	-0.46820771	-1.38339	0.023508348
55093_at	chondroitin sulfate glucuronyltransferase	CSGlcA-T	-0.46834221	-1.38352	0.008103638
203974_at	haloacid dehalogenase-like hydrolase domain containing 1A	HDHD1A	-0.46845141	-1.38362	0.036520037
219499_at	Sec61 alpha 2 subunit (S. cerevisiae)	SEC61A2	-0.46861263	-1.38378	0.020445743
213310_at	Eukaryotic translation initiation factor 2C, 2	EIF2C2	-0.46908074	-1.38423	0.03789462
211754_s_at	solute carrier family 25 (mitochondrial carrier; peroxisomal membrane protein, 34kDa), member 17	SLC25A17	-0.46941503	-1.38455	0.036846349
221669_s_at	acyl-Coenzyme A dehydrogenase family, member 8	ACAD8	-0.46994017	-1.38505	0.028976133

220318_at	epsin 3	EPN3	-0.47066715	-1.38575	0.0350019
221277_s_at	pseudouridylate synthase 3	PUS3	-0.47128096	-1.38634	0.011149119
219340_s_at	ceroid-lipofuscinosis, neuronal 8 (epilepsy, progressive with mental retardation)	CLN8	-0.4725815	-1.38759	0.013677352
202594_at	leptin receptor overlapping transcript-like 1	LEPROTL1	-0.47271752	-1.38772	0.03601083
211068_x_at	family with sequence similarity 21, member C /// family with sequence similarity 21, member D	FAM21C //	-0.47296675	-1.38796	0.014546584
213798_s_at	CAP, adenylate cyclase-associated protein 1 (yeast)	CAP1	-0.47481335	-1.38974	0.046553557
204986_s_at	TAO kinase 2	TAOK2	-0.47594235	-1.39083	0.03216506
212660_at	PHD finger protein 15	PHF15	-0.47602558	-1.39091	0.023632473
213604_at	CDNA FLJ42849 fis, A-BRHIP2004902		-0.47608736	-1.39097	0.034249673
208810_at	DnaJ (Hsp40) homolog, subfamily B, member 6	DNAJB6	-0.47850237	-1.3933	0.036846349
218571_s_at	chromatin modifying protein 4A	CHMP4A	-0.47862938	-1.39342	0.023015457
219460_s_at	transmembrane protein 127	TMEM127	-0.47889886	-1.39368	0.017071662
209905_at	homeobox A9	HOXA9	-0.47929291	-1.39406	0.027725725
208685_x_at	bromodomain containing 2	BRD2	-0.47963978	-1.3944	0.017353002
209896_s_at	protein tyrosine phosphatase, non-receptor type 11 (Noonan syndrome 1)	PTPN11	-0.47984001	-1.39459	0.018190072
209587_at	paired-like homeodomain 1	PITX1	-0.48074253	-1.39546	0.021384427
208832_at	ataxin 10	ATXN10	-0.48164857	-1.39634	0.014672661
221535_at	large subunit GTPase 1 homolog (S. cerevisiae)	LSG1	-0.48210322	-1.39678	0.035341012
203164_at	solute carrier family 33 (acetyl-CoA transporter), member 1	SLC33A1	-0.48285715	-1.39751	0.033997175
202884_s_at	protein phosphatase 2 (formerly 2A), regulatory subunit A, beta isoform	PPP2R1B	-0.48326098	-1.3979	0.008488821
214948_s_at	TATA element modulatory factor 1	TMF1	-0.48348067	-1.39811	0.008459494
200867_at	zinc finger protein 313	ZNF313	-0.48369208	-1.39832	0.032015473
202819_s_at	transcription elongation factor B (SIII), polypeptide 3 (110kDa, elongin A)	TCEB3	-0.48451394	-1.39911	0.017238997
212340_at	Yip1 domain family, member 6	YIPF6	-0.48455534	-1.39915	0.009425165

212644_s_at	mitogen-activated protein kinase 1 interacting protein 1-like	MAPK1IP1l	-0.48470269	-1.3993	0.016988706
200729_s_at	ARP2 actin-related protein 2 homolog (yeast)	ACTR2	-0.48475327	-1.39935	0.031945762
217882_at	transmembrane protein 111	TMEM111	-0.48658586	-1.40113	0.040753506
218488_at	eukaryotic translation initiation factor 2B, subunit 3 gamma, 58kDa	EIF2B3	-0.48784116	-1.40234	0.041087454
220659_s_at	chromosome 7 open reading frame 43	C7orf43	-0.48837215	-1.40286	0.006949425
219217_at	asparaginyl-tRNA synthetase 2, mitochondrial (putative)	NARS2	-0.48930477	-1.40377	0.040897992
210191_s_at	putative homeodomain transcription factor 1	PHTF1	-0.49015072	-1.40459	0.020400033
218572_at	chromatin modifying protein 4A	CHMP4A	-0.49070025	-1.40513	0.013481296
221571_at	TNF receptor-associated factor 3	TRAF3	-0.49108565	-1.4055	0.043534978
219215_s_at	solute carrier family 39 (zinc transporter), member 4	SLC39A4	-0.49128466	-1.4057	0.015364265
218065_s_at	TMEM9 domain family, member B	TMEM9B	-0.49142566	-1.40583	0.045715027
209858_x_at	metallophosphoesterase 1	MPPE1	-0.49171084	-1.40611	0.015434646
209679_s_at	small trans-membrane and glycosylated protein	LOC57228	-0.49208774	-1.40648	0.032801415
31807_at	DEAD (Asp-Glu-Ala-Asp) box polypeptide 49	DDX49	-0.49308097	-1.40745	0.011534663
213902_at	N-acylsphingosine amidohydrolase (acid ceramidase) 1	ASAH1	-0.49345077	-1.40781	0.01814502
220145_at	microtubule-associated protein 9	MAP9	-0.49365744	-1.40801	0.016850455
213410_at	chromosome 10 open reading frame 137	C10orf137	-0.49403859	-1.40838	0.020085667
217797_at	ubiquitin-fold modifier conjugating enzyme 1	UFC1	-0.49467666	-1.409	0.047706878
203738_at	chromosome 5 open reading frame 22	C5orf22	-0.49469693	-1.40902	0.045983569
221190_s_at	chromosome 18 open reading frame 8	C18orf8	-0.49501959	-1.40934	0.026599732
213119_at	solute carrier family 36 (proton/amino acid symporter), member 1	SLC36A1	-0.49553932	-1.40985	0.005627889
204067_at	sulfite oxidase	SUOX	-0.49605756	-1.41035	0.018665191
218374_s_at	chromosome 12 open reading frame 4	C12orf4	-0.49669517	-1.41098	0.024461961

213727_x_at	metallophosphoesterase 1	MPPE1	-0.49713846	-1.41141	0.014664842
218327_s_at	synaptosomal-associated protein, 29kDa	SNAP29	-0.49906686	-1.4133	0.020059581
201711_x_at	RAN binding protein 2	RANBP2	-0.49926319	-1.41349	0.017236527
210959_s_at	steroid-5-alpha-reductase, alpha polypeptide 1 (3-oxo-5 alpha-steroid delta 4-dehydrogenase alpha 1)	SRD5A1	-0.49943071	-1.41366	0.04032077
220038_at	serum/glucocorticoid regulated kinase family, member 3 /// chromosome 8 open reading frame 44	C8orf44 //,	-0.499731	-1.41395	0.039856933
212804_s_at	GTPase activating protein and VPS9 domains 1	GAPVD1	-0.50046724	-1.41467	0.04793172
209231_s_at	dynactin 5 (p25)	DCTN5	-0.50050708	-1.41471	0.022300352
205661_s_at	FAD1 flavin adenine dinucleotide synthetase homolog (S. cerevisiae)	FLAD1	-0.50176769	-1.41595	0.047363623
213153_at	SET domain containing 1B	SETD1B	-0.50206803	-1.41624	0.012241734
213571_s_at	eukaryotic translation initiation factor 4E family member 2	EIF4E2	-0.50212916	-1.4163	0.020743425
203428_s_at	ASF1 anti-silencing function 1 homolog A (S. cerevisiae)	ASF1A	-0.50232517	-1.41649	0.032426062
217722_s_at	neugrin, neurite outgrowth associated	NGRN	-0.50265589	-1.41682	0.046356918
218028_at	elongation of very long chain fatty acids (FEN1/Elo2, SUR4/Elo3, yeast)-like 1	ELOVL1	-0.50444125	-1.41857	0.015796973
203580_s_at	solute carrier family 7 (cationic amino acid transporter, y+ system), member 6 /// transient receptor potential cation channel, subfamily V, member 6	SLC7A6 ///	-0.50538919	-1.41951	0.017797984
218416_s_at	hypothetical protein FLJ20489	FLJ20489	-0.50602325	-1.42013	0.020891228
209751_s_at	trafficking protein particle complex 2 /// spondyloepiphyseal dysplasia, late, pseudogene /// zinc finger protein 547	SEDLP /// T	-0.50605597	-1.42016	0.035334764
212101_at	karyopherin alpha 6 (importin alpha 7)	KPNA6	-0.50824223	-1.42232	0.045565067
203415_at	programmed cell death 6	PDCD6	-0.50833068	-1.4224	0.045899526

203054_s_at	T-cell leukemia translocation altered gene	TCTA	-0.508807	-1.42287	0.02597525
221736_at	KIAA1219	KIAA1219	-0.50901589	-1.42308	0.049733581
213357_at	general transcription factor IIH, polypeptide 5	GTF2H5	-0.50901783	-1.42308	0.042105187
214949_at	CDNA FLJ31919 fis, clone NT2RP7004964		-0.50926507	-1.42332	0.008546266
205809_s_at	Wiskott-Aldrich syndrome-like	WASL	-0.50933178	-1.42339	0.02897476
210811_s_at	DEAD (Asp-Glu-Ala-Asp) box polypeptide 49	DDX49	-0.50933405	-1.42339	0.008191654
201935_s_at	eukaryotic translation initiation factor 4 gamma, 3	EIF4G3	-0.50964387	-1.4237	0.015797948
214314_s_at	eukaryotic translation initiation factor 5B	EIF5B	-0.51032422	-1.42437	0.041547223
204179_at	myoglobin	MB	-0.51055395	-1.4246	0.047363623
202296_s_at	RER1 retention in endoplasmic reticulum 1 homolog (S. cerevisiae)	RER1	-0.511521	-1.42555	0.019624023
221536_s_at	large subunit GTPase 1 homolog (S. cerevisiae)	LSG1	-0.51160429	-1.42563	0.049628339
215684_s_at	activating signal cointegrator 1 complex subunit 2	ASCC2	-0.5116218	-1.42565	0.037146889
201733_at	chloride channel 3	CLCN3	-0.51199824	-1.42602	0.03149693
220144_s_at	ankyrin repeat domain 5	ANKRD5	-0.51202822	-1.42605	0.018965453
208066_s_at	general transcription factor IIB	GTF2B	-0.51255419	-1.42657	0.020891228
201023_at	TAF7 RNA polymerase II, TATA box binding protein (TBP)-associated factor, 55kDa	TAF7	-0.51458447	-1.42858	0.020112851
201776_s_at	KIAA0494	KIAA0494	-0.51476395	-1.42876	0.022760006
212877_at	kinesin light chain 1	KLC1	-0.51487927	-1.42887	0.014788456
219474_at	chromosome 3 open reading frame 52	C3orf52	-0.51575052	-1.42974	0.02449946
212601_at	zinc finger, ZZ-type with EF-hand domain 1	ZZEF1	-0.51582444	-1.42981	0.003901445
213478_at	kazrin	RP1-21O18	-0.51675976	-1.43074	0.040987387
203781_at	mitochondrial ribosomal protein L33	MRPL33	-0.51738334	-1.43136	0.045441388
219909_at	matrix metallopeptidase 28	MMP28	-0.51757135	-1.43154	0.034375372
204058_at	malic enzyme 1, NADP(+)-dependent, cytosolic	ME1	-0.51794489	-1.43191	0.046968211
204278_s_at	estrogen receptor binding site associated, antigen, 9	EBAG9	-0.51814616	-1.43211	0.031032444

221210_s_at	N-acetylneuraminate pyruvate lyase (dihydrodipicolinate synthase)	NPL	-0.51998674	-1.43394	0.006765098
221582_at	histone cluster 3, H2a	HIST3H2A	-0.52003159	-1.43399	0.034142802
218538_s_at	MRS2 magnesium homeostasis factor homolog (S. cerevisiae)	MRS2	-0.52022126	-1.43418	0.017027365
203360_s_at	c-myc binding protein	MYCBP	-0.52061296	-1.43456	0.038182423
203226_s_at	tetraspanin 31	TSPAN31	-0.52072798	-1.43468	0.029644613
57163_at	elongation of very long chain fatty acids (FEN1/Elo2, SUR4/Elo3, yeast)-like 1	ELOVL1	-0.52113718	-1.43509	0.019624023
201274_at	proteasome (prosome, macropain) subunit, alpha type, 5	PSMA5	-0.5211824	-1.43513	0.041095513
203266_s_at	mitogen-activated protein kinase kinase 4	MAP2K4	-0.52147852	-1.43543	0.015909665
206953_s_at	latrophilin 2	LPHN2	-0.52191777	-1.43586	0.016850455
220761_s_at	TAO kinase 3	TAOK3	-0.5226027	-1.43654	0.02597525
204204_at	solute carrier family 31 (copper transporters), member 2	SLC31A2	-0.52299521	-1.43694	0.028312284
207430_s_at	microseminoprotein, beta-	MSMB	-0.52462392	-1.43856	0.016606812
205690_s_at	BUD31 homolog (S. cerevisiae)	BUD31	-0.52627639	-1.44021	0.015434646
219597_s_at	dual oxidase 1	DUOX1	-0.52723657	-1.44117	0.018372499
213351_s_at	transmembrane and coiled-coil domain family 1	TMCC1	-0.52724027	-1.44117	0.006921301
200728_at	ARP2 actin-related protein 2 homolog (yeast)	ACTR2	-0.52783362	-1.44176	0.020400033
219600_s_at	transmembrane protein 50B	TMEM50B	-0.52848347	-1.44241	0.032430413
218347_at	tRNA-yW synthesizing protein 1 homolog (S. cerevisiae)	TYW1	-0.52851661	-1.44245	0.007083702
209463_s_at	TAF12 RNA polymerase II, TATA box binding protein (TBP)-associated factor, 20kDa	TAF12	-0.52889315	-1.44282	0.015889948
218840_s_at	NAD synthetase 1	NADSYN1	-0.52907016	-1.443	0.012959052
209384_at	proline synthetase co-transcribed homolog (bacterial)	PROSC	-0.52907563	-1.443	0.02325585
221256_s_at	haloacid dehalogenase-like hydrolase domain containing 3	HDHD3	-0.52950601	-1.44343	0.038182423
221542_s_at	ER lipid raft associated 2	ERLIN2	-0.52996024	-1.44389	0.044130621

202070_s_at	isocitrate dehydrogenase 3 (NAD+) alpha	IDH3A	-0.53138714	-1.44532	0.022760006
203272_s_at	tumor suppressor candidate 2	TUSC2	-0.53327043	-1.44721	0.012973447
201192_s_at	phosphatidylinositol transfer protein, alpha	PITPNA	-0.53401265	-1.44795	0.01323345
215884_s_at	ubiquilin 2	UBQLN2	-0.53480095	-1.44874	0.023195616
218260_at	DET1 and DDB1 associated 1	DDA1	-0.53492087	-1.44886	0.043855347
213637_at	Transcribed locus		-0.53517429	-1.44912	0.006765098
209157_at	DnaJ (Hsp40) homolog, subfamily A, member 2	DNAJA2	-0.53520062	-1.44914	0.010060198
211423_s_at	sterol-C5-desaturase (ERG3 delta-5-desaturase homolog, S. cerevisiae)-like	SC5DL	-0.53689421	-1.45085	0.022300352
214455_at	histone cluster 1, H2bg /// histone cluster 1, H2bf /// histone cluster 1, H2be /// histone cluster 1, H2bi /// histone cluster 1, H2bc	HIST1H2BC	-0.53766649	-1.45162	0.016291216
220414_at	calmodulin-like 5	CALML5	-0.53792339	-1.45188	0.018190072
214911_s_at	bromodomain containing 2	BRD2	-0.53921868	-1.45319	0.0092838
201142_at	eukaryotic translation initiation factor 2, subunit 1 alpha, 35kDa	EIF2S1	-0.54210115	-1.45609	0.019886651
218797_s_at	sirtuin (silent mating type information regulation 2 homolog) 7 (S. cerevisiae)	SIRT7	-0.54288131	-1.45688	0.027725725
213073_at	zinc finger, FYVE domain containing 26	ZFYVE26	-0.5430382	-1.45704	0.011345489
203762_s_at	dynein, cytoplasmic 2, light intermediate chain 1	DYNC2LI1	-0.54366546	-1.45767	0.009825362
202595_s_at	leptin receptor overlapping transcript-like 1	LEPROTL1	-0.54398596	-1.458	0.006994923
209926_at	myocyte enhancer factor 2B	MEF2B	-0.54557613	-1.4596	0.005627889
208899_x_at	ATPase, H+ transporting, lysosomal 34kDa, V1 subunit D	ATP6V1D	-0.54664546	-1.46069	0.039353364
209186_at	ATPase, Ca++ transporting, cardiac muscle, slow twitch 2	ATP2A2	-0.54761273	-1.46167	0.028368833
211413_s_at	peptidyl arginine deiminase, type IV	PADI4	-0.54765233	-1.46171	0.009110746
201024_x_at	eukaryotic translation initiation factor 5B	EIF5B	-0.54910532	-1.46318	0.026683706
216905_s_at	suppression of tumorigenicity 14 (colon carcinoma)	ST14	-0.54925079	-1.46333	0.042348015

222105_s_at	NFKB inhibitor interacting Ras-like 2	NKIRAS2	-0.55113687	-1.46524	0.030686502
212643_at	mitogen-activated protein kinase 1 interacting protein 1-like	MAPK1IP1l	-0.55121814	-1.46532	0.018965453
218135_at	ERGIC and golgi 2	ERGIC2	-0.55144167	-1.46555	0.023431113
202069_s_at	isocitrate dehydrogenase 3 (NAD+) alpha	IDH3A	-0.55220253	-1.46632	0.042138914
220202_s_at	ring finger and CCCH-type zinc finger domains 2	RC3H2	-0.55404275	-1.46819	0.036846349
214830_at	solute carrier family 38, member 6	SLC38A6	-0.55525584	-1.46943	0.022965983
203159_at	glutaminase	GLS	-0.55580566	-1.46999	0.042323058
214598_at	claudin 8	CLDN8	-0.55689811	-1.4711	0.03539949
212070_at	G protein-coupled receptor 56	GPR56	-0.55855991	-1.4728	0.049628339
213352_at	transmembrane and coiled-coil domain family 1	TMCC1	-0.55995482	-1.47422	0.022048788
204177_s_at	kelch-like 20 (Drosophila)	KLHL20	-0.56559033	-1.47999	0.017702307
219760_at	lin-7 homolog B (C. elegans)	LIN7B	-0.56594757	-1.48036	0.010235373
220998_s_at	unc-93 homolog B1 (C. elegans)	UNC93B1	-0.56629708	-1.48072	0.00887733
218262_at	required for meiotic nuclear division 5 homolog B (S. cerevisiae)	RMND5B	-0.56677166	-1.48121	0.016101691
208114_s_at	interferon stimulated exonuclease gene 20kDa-like 2	ISG20L2	-0.56719846	-1.48164	0.049261748
212451_at	KIAA0256 gene product	KIAA0256	-0.56954452	-1.48405	0.049942643
214695_at	ubiquitin associated protein 2-like	UBAP2L	-0.56963621	-1.48415	0.011300989
209425_at	alpha-methylacyl-CoA racemase /// C1q and tumor necrosis factor related protein 3	AMACR ///	-0.56973948	-1.48426	0.004895765
218224_at	paraneoplastic antigen MA1	PNMA1	-0.57145034	-1.48602	0.008459494
217794_at	proline rich 13	PRR13	-0.57337567	-1.488	0.011833306
209580_s_at	methyl-CpG binding domain protein 4	MBD4	-0.57501958	-1.4897	0.012448129
219832_s_at	homeobox C13	HOXC13	-0.57547026	-1.49016	0.006994923
208945_s_at	beclin 1, autophagy related	BECN1	-0.57568984	-1.49039	0.047719364
212481_s_at	tropomyosin 4	TPM4	-0.57585961	-1.49057	0.030951478
217751_at	glutathione S-transferase kappa 1	GSTK1	-0.57614132	-1.49086	0.028552561
219696_at	chromosome 1 open reading frame 218 /// DENN/MADD domain containing 1B	C1orf218 /,	-0.57614991	-1.49087	0.030816874

213448_at	CDNA FLJ31688 fis, clone NT2RI2005520		-0.57819121	-1.49298	0.018922783
202306_at	polymerase (RNA) II (DNA directed) polypeptide G	POLR2G	-0.5789937	-1.49381	0.01671059
212908_at	DnaJ (Hsp40) homolog, subfamily C, member 16	DNAJC16	-0.57905545	-1.49387	0.006921301
213220_at	hypothetical LOC92482	LOC92482	-0.57926935	-1.49409	0.017095271
202128_at	KIAA0317	KIAA0317	-0.57952835	-1.49436	0.017917278
218915_at	neurofibromin 2 (merlin)	NF2	-0.57964541	-1.49448	0.02449946
217912_at	dihydrouridine synthase 1-like (S. cerevisiae)	DUS1L	-0.58180719	-1.49672	0.03376599
208898_at	ATPase, H+ transporting, lysosomal 34kDa, V1 subunit D	ATP6V1D	-0.58264513	-1.49759	0.042148929
203665_at	heme oxygenase (decycling) 1	HMOX1	-0.58362127	-1.49861	0.047064544
201821_s_at	translocase of inner mitochondrial membrane 17 homolog A (yeast)	TIMM17A	-0.58427814	-1.49929	0.027147145
208546_x_at	histone cluster 1, H2bh	HIST1H2BH	-0.58493742	-1.49997	0.012973447
209300_s_at	NECAP endocytosis associated 1	NECAP1	-0.58585706	-1.50093	0.006293525
201349_at	solute carrier family 9 (sodium/hydrogen exchanger), member 3 regulator 1	SLC9A3R1	-0.58717208	-1.5023	0.022122228
205259_at	nuclear receptor subfamily 3, group C, member 2	NR3C2	-0.58742575	-1.50256	0.003901445
206448_at	zinc finger protein 365	ZNF365	-0.59060867	-1.50588	0.017353002
218032_at	stannin	SNN	-0.59189185	-1.50722	0.046269754
204059_s_at	malic enzyme 1, NADP(+)-dependent, cytosolic	ME1	-0.59195624	-1.50729	0.033679601
202125_s_at	trafficking protein, kinesin binding 2	TRAK2	-0.5932626	-1.50865	0.0102878
221122_at	HRAS-like suppressor 2	HRASLS2	-0.59350735	-1.50891	0.010853986
214168_s_at	tight junction protein 1 (zona occludens 1)	TJP1	-0.59744698	-1.51304	0.017797984
218403_at	TP53 regulated inhibitor of apoptosis 1	TRIAP1	-0.59957917	-1.51527	0.016698837
211712_s_at	annexin A9	ANXA9	-0.59972122	-1.51542	0.036557389
214721_x_at	CDC42 effector protein (Rho GTPase binding) 4	CDC42EP4	-0.60010403	-1.51583	0.040783358
204242_s_at	acyl-Coenzyme A oxidase 3, pristanoyl	ACOX3	-0.60060812	-1.51636	0.009555655
219770_at	glycosyltransferase-like domain containing 1	GTDC1	-0.6012403	-1.51702	0.017979101

220647_s_at	coiled-coil-helix-coiled-coil-helix domain containing 8	CHCHD8	-0.60230653	-1.51814	0.032276785
201971_s_at	ATPase, H+ transporting, lysosomal 70kDa, V1 subunit A	ATP6V1A	-0.60751396	-1.52363	0.0307204
209424_s_at	alpha-methylacyl-CoA racemase /// C1q and tumor necrosis factor related protein 3	AMACR ///	-0.60960808	-1.52584	0.005627889
209385_s_at	proline synthetase co-transcribed homolog (bacterial)	PROSC	-0.61005223	-1.52631	0.039944948
204137_at	G protein-coupled receptor 137B	GPR137B	-0.61051005	-1.5268	0.012522351
210075_at	membrane-associated ring finger (C3HC4) 2	2-Mar	-0.61180627	-1.52817	0.015830371
213083_at	solute carrier family 35, member D2	SLC35D2	-0.61210995	-1.52849	0.010853986
218357_s_at	translocase of inner mitochondrial membrane 8 homolog B (yeast)	TIMM8B	-0.61419201	-1.5307	0.02597525
201768_s_at	clathrin interactor 1	CLINT1	-0.61433257	-1.53085	0.045510962
218126_at	family with sequence similarity 82, member C	FAM82C	-0.61458078	-1.53111	0.009825362
203123_s_at	solute carrier family 11 (proton-coupled divalent metal ion transporters), member 2	SLC11A2	-0.61514321	-1.53171	0.049565682
201732_s_at	chloride channel 3	CLCN3	-0.61564532	-1.53224	0.008601198
211676_s_at	interferon gamma receptor 1	IFNGR1	-0.62033344	-1.53723	0.048665054
200965_s_at	actin binding LIM protein 1	ABLIM1	-0.62188162	-1.53888	0.006765098
208527_x_at	histone cluster 1, H2bg /// histone cluster 1, H2bf /// histone cluster 1, H2be /// histone cluster 1, H2bi /// histone cluster 1, H2bc	HIST1H2BC	-0.6250554	-1.54227	0.011960948
209486_at	UTP3, small subunit (SSU) processome component, homolog (S. cerevisiae)	UTP3	-0.62510619	-1.54232	0.025799468
213082_s_at	solute carrier family 35, member D2	SLC35D2	-0.62737594	-1.54475	0.009152332
213031_s_at	WD repeat domain 73	WDR73	-0.62869639	-1.54617	0.010739422
202395_at	N-ethylmaleimide-sensitive factor	NSF	-0.62936346	-1.54688	0.03150325
212993_at	BTB (POZ) domain containing 14A	BTBD14A	-0.63189339	-1.5496	0.001616605

218942_at	phosphatidylinositol-5-phosphate 4-kinase, type II, gamma	PIP4K2C	-0.63309275	-1.55089	0.02597525
203608_at	aldehyde dehydrogenase 5 family, member A1 (succinate-semialdehyde dehydrogenase)	ALDH5A1	-0.63339052	-1.55121	0.005627889
217835_x_at	chromosome 20 open reading frame 24	C20orf24	-0.63387964	-1.55173	0.026683706
203253_s_at	histidine acid phosphatase domain containing 1	HISPPD1	-0.63477571	-1.5527	0.019624023
204341_at	tripartite motif-containing 16	TRIM16	-0.63683103	-1.55491	0.037399893
205749_at	cytochrome P450, family 1, subfamily A, polypeptide 1	CYP1A1	-0.63932075	-1.5576	0.010165029
203304_at	BMP and activin membrane-bound inhibitor homolog (Xenopus laevis)	BAMBI	-0.63952756	-1.55782	0.003901445
206235_at	ligase IV, DNA, ATP-dependent	LIG4	-0.64642847	-1.56529	0.012476914
201190_s_at	phosphatidylinositol transfer protein, alpha	PITPNA	-0.64722982	-1.56616	0.00887733
213704_at	Rab geranylgeranyltransferase, beta subunit	RABGGTB	-0.64867158	-1.56772	0.042587712
205966_at	TAF13 RNA polymerase II, TATA box binding protein (TBP)-associated factor, 18kDa	TAF13	-0.64878342	-1.56785	0.036694406
216048_s_at	Rho-related BTB domain containing 3	RHOBTB3	-0.65083626	-1.57008	0.045334327
203158_s_at	glutaminase	GLS	-0.65664122	-1.57641	0.006949425
206295_at	interleukin 18 (interferon-gamma-inducing factor)	IL18	-0.66018018	-1.58028	0.036520037
202842_s_at	DnaJ (Hsp40) homolog, subfamily B, member 9	DNAJB9	-0.66224692	-1.58255	0.006949425
204667_at	forkhead box A1	FOXA1	-0.66372455	-1.58417	0.005627889
221046_s_at	GTP-binding protein 8 (putative)	GTPBP8	-0.66471266	-1.58525	0.040001317
209094_at	dimethylarginine dimethylaminohydrolase 1	DDAH1	-0.66495681	-1.58552	0.015021093
221787_at	chromosome 6 open reading frame 120	C6orf120	-0.66616423	-1.58685	0.017163466
205155_s_at	spectrin, beta, non-erythrocytic 2	SPTBN2	-0.66618843	-1.58687	0.023822395
203273_s_at	tumor suppressor candidate 2	TUSC2	-0.66864164	-1.58958	0.002990116

204241_at	acyl-Coenzyme A oxidase 3, pristanoyl	ACOX3	-0.66925546	-1.59025	0.010853986
206061_s_at	dicer 1, ribonuclease type III	DICER1	-0.66967348	-1.59071	0.033624312
209426_s_at	alpha-methylacyl-CoA racemase /// C1q and tumor necrosis factor related protein 3	AMACR ///	-0.67237816	-1.5937	0.004387256
218150_at	ADP-ribosylation factor-like 5A	ARL5A	-0.6727616	-1.59412	0.018190072
202149_at	neural precursor cell expressed, developmentally down-regulated 9	NEDD9	-0.6728883	-1.59426	0.016108825
211383_s_at	WD repeat domain 37	WDR37	-0.67330097	-1.59472	0.007131295
222165_x_at	chromosome 9 open reading frame 16	C9orf16	-0.6761547	-1.59788	0.009825362
202626_s_at	v-yes-1 Yamaguchi sarcoma viral related oncogene homolog	LYN	-0.67827503	-1.60023	0.02897476
214651_s_at	homeobox A9	HOXA9	-0.67927554	-1.60134	0.012522351
209665_at	cytochrome b-561 domain containing 2	CYB561D2	-0.68132747	-1.60361	0.016278635
205770_at	glutathione reductase	GSR	-0.68175005	-1.60408	0.010853986
208510_s_at	peroxisome proliferator-activated receptor gamma	PPARG	-0.68272318	-1.60517	0.009110746
206118_at	signal transducer and activator of transcription 4	STAT4	-0.68577598	-1.60857	0.047363623
201996_s_at	spen homolog, transcriptional regulator (Drosophila)	SPEN	-0.68703495	-1.60997	0.035860651
218853_s_at	motile sperm domain containing 1	MOSPD1	-0.68990711	-1.61318	0.01579422
203869_at	ubiquitin specific peptidase 46	USP46	-0.69113828	-1.61456	0.006765098
201191_at	phosphatidylinositol transfer protein, alpha	PITPNA	-0.6926668	-1.61627	0.027540443
204881_s_at	UDP-glucose ceramide glucosyltransferase	UGCG	-0.69538564	-1.61932	0.012448129
211985_s_at	calmodulin 1 (phosphorylase kinase, delta) /// calmodulin 2 (phosphorylase kinase, delta) /// calmodulin 3 (phosphorylase kinase, delta)	CALM1 ///	-0.69623067	-1.62027	0.046356918
214719_at	solute carrier family 46, member 3	SLC46A3	-0.69774285	-1.62197	0.016108825
209304_x_at	growth arrest and DNA-damage-inducible, beta	GADD45B	-0.69880838	-1.62316	0.039944948

201975_at	CAP-GLY domain containing linker protein 1	CLIP1	-0.69909929	-1.62349	0.04302319
201735_s_at	chloride channel 3	CLCN3	-0.70265461	-1.6275	0.013481296
203743_s_at	thymine-DNA glycosylase	TDG	-0.70272108	-1.62757	0.022981502
209600_s_at	acyl-Coenzyme A oxidase 1, palmitoyl	ACOX1	-0.7050067	-1.63015	0.022346379
210589_s_at	glucosidase, beta; acid (includes glucosylceramidase) /// glucosidase, beta; acid, pseudogene	GBA /// GE	-0.70721722	-1.63265	0.006293525
205473_at	ATPase, H+ transporting, lysosomal 56/58kDa, V1 subunit B1 (Renal tubular acidosis with deafness)	ATP6V1B1	-0.70845821	-1.63406	0.009849164
215171_s_at	translocase of inner mitochondrial membrane 17 homolog A (yeast)	TIMM17A	-0.70906468	-1.63474	0.035264267
202124_s_at	trafficking protein, kinesin binding 2	TRAK2	-0.7108598	-1.63678	0.014664842
210130_s_at	transmembrane 7 superfamily member 2	TM7SF2	-0.71305295	-1.63927	0.024704744
203454_s_at	ATX1 antioxidant protein 1 homolog (yeast)	ATOX1	-0.71447693	-1.64089	0.032430413
201340_s_at	ectodermal-neural cortex (with BTB-like domain)	ENC1	-0.71989879	-1.64707	0.013044506
209694_at	6-pyruvoyltetrahydropterin synthase	PTS	-0.72043398	-1.64768	0.026965005
203053_at	breast carcinoma amplified sequence 2	BCAS2	-0.72172339	-1.64915	0.009825362
210720_s_at	N-terminal EF-hand calcium binding protein 3	NECAB3	-0.724567	-1.6524	0.00887733
213203_at	small nuclear RNA activating complex, polypeptide 5, 19kDa	SNAPC5	-0.72493986	-1.65283	0.018311616
207540_s_at	spleen tyrosine kinase	SYK	-0.72914669	-1.65766	0.005533101
31846_at	ras homolog gene family, member D	RHOD	-0.73000497	-1.65864	0.043925801
214733_s_at	Yip1 domain family, member 1	YIPF1	-0.73019677	-1.65887	0.022300352
212875_s_at	C2 calcium-dependent domain containing 2	C2CD2	-0.73077704	-1.65953	0.007610795
218536_at	MRS2 magnesium homeostasis factor homolog (S. cerevisiae)	MRS2	-0.73170135	-1.6606	0.001616605
212441_at	KIAA0232	KIAA0232	-0.73285449	-1.66192	0.017818048

205442_at	microfibrillar-associated protein 3-like	MFAP3L	-0.73506289	-1.66447	0.008191654
204068_at	serine/threonine kinase 3 (STE20 homolog, yeast)	STK3	-0.74075392	-1.67105	0.047706878
212415_at	septin 6	6-Sep	-0.74180086	-1.67226	0.006293525
205640_at	aldehyde dehydrogenase 3 family, member B1	ALDH3B1	-0.7430995	-1.67377	0.018190072
219099_at	chromosome 12 open reading frame 5	C12orf5	-0.74389294	-1.67469	0.034142802
218706_s_at	GRAM domain containing 3	GRAMD3	-0.74511873	-1.67611	0.004902615
212624_s_at	chimerin (chimaerin) 1	CHN1	-0.74665525	-1.6779	0.033775564
210980_s_at	N-acylsphingosine amidohydrolase (acid ceramidase) 1	ASAH1	-0.74672501	-1.67798	0.045562906
204048_s_at	phosphatase and actin regulator 2	PHACTR2	-0.74759272	-1.67899	0.007257672
203691_at	peptidase inhibitor 3, skin-derived (SKALP)	PI3	-0.74962606	-1.68136	0.040987387
203594_at	RNA terminal phosphate cyclase domain 1	RTCD1	-0.75145627	-1.68349	0.007051473
218417_s_at	hypothetical protein FLJ20489	FLJ20489	-0.75604598	-1.68886	0.014283294
207601_at	sulfotransferase family, cytosolic, 1B, member 1	SULT1B1	-0.76648336	-1.70112	0.002990116
221622_s_at	transmembrane protein 126B	TMEM126I	-0.77203354	-1.70768	0.015820294
218976_at	DnaJ (Hsp40) homolog, subfamily C, member 12	DNAJC12	-0.77849358	-1.71534	0.006293525
205248_at	dopey family member 2	DOPEY2	-0.77887739	-1.7158	0.007257672
202948_at	interleukin 1 receptor, type I	IL1R1	-0.78040188	-1.71761	0.038182423
201079_at	synaptogyrin 2	SYNGR2	-0.78079459	-1.71808	0.022760006
209093_s_at	glucosidase, beta; acid (includes glucosylceramidase) /// glucosidase, beta; acid, pseudogene	GBA /// GE	-0.78132114	-1.7187	0.005627889
219428_s_at	peroxisomal membrane protein 4, 24kDa	PXMP4	-0.7820371	-1.71956	0.047324525
207843_x_at	cytochrome b5 type A (microsomal)	CYB5A	-0.78377842	-1.72163	0.023195616
210297_s_at	microseminoprotein, beta-	MSMB	-0.7838985	-1.72178	0.004387256
202951_at	serine/threonine kinase 38	STK38	-0.78558143	-1.72379	0.011512478
211795_s_at	FYN binding protein (FYB-120/130)	FYB	-0.78679134	-1.72523	0.011424114
212528_at	CDNA clone IMAGE:3878236		-0.7868141	-1.72526	0.004895765
204435_at	nucleoporin like 1	NUPL1	-0.79333	-1.73307	0.036078545

201143_s_at	eukaryotic translation initiation factor 2, subunit 1 alpha, 35kDa	EIF2S1	-0.80118216	-1.74253	0.006293525
206599_at	solute carrier family 16, member 5 (monocarboxylic acid transporter 6) /// similar to MCT	LOC10013:	-0.80368683	-1.74556	0.003572379
209180_at	Rab geranylgeranyltransferase, beta subunit	RABGGTB	-0.80536408	-1.74759	0.011534663
208744_x_at	heat shock 105kDa/110kDa protein 1	HSPH1	-0.80941382	-1.7525	0.020336612
203559_s_at	amiloride binding protein 1 (amine oxidase (copper-containing))	ABP1	-0.81263965	-1.75642	0.021823482
221786_at	chromosome 6 open reading frame 120	C6orf120	-0.81355497	-1.75754	0.016199453
221552_at	abhydrolase domain containing 6	ABHD6	-0.8148179	-1.75908	0.003901445
214696_at	chromosome 17 open reading frame 91	C17orf91	-0.81579243	-1.76026	0.011512478
48106_at	hypothetical protein FLJ20489	FLJ20489	-0.81757032	-1.76244	0.006808225
209310_s_at	caspase 4, apoptosis-related cysteine peptidase	CASP4	-0.81985113	-1.76522	0.004387256
209366_x_at	cytochrome b5 type A (microsomal)	CYB5A	-0.82529506	-1.7719	0.024228831
45288_at	abhydrolase domain containing 6	ABHD6	-0.82691688	-1.77389	0.004895765
213702_x_at	N-acylsphingosine amidohydrolase (acid ceramidase) 1	ASAH1	-0.82793747	-1.77515	0.034446605
207121_s_at	mitogen-activated protein kinase 6	MAPK6	-0.82848301	-1.77582	0.040010642
209882_at	Ras-like without CAAX 1	RIT1	-0.82852415	-1.77587	0.021384427
65517_at	adaptor-related protein complex 1, mu 2 subunit	AP1M2	-0.83061336	-1.77844	0.019984778
218261_at	adaptor-related protein complex 1, mu 2 subunit	AP1M2	-0.83154677	-1.77959	0.036212513
217499_x_at	olfactory receptor, family 7, subfamily E, member 37 pseudogene	OR7E37P	-0.83637865	-1.78556	0.007043637
206059_at	zinc finger protein 91	ZNF91	-0.8372735	-1.78667	0.016606812
209950_s_at	villin-like	VILL	-0.83982634	-1.78983	0.003930777
218532_s_at	family with sequence similarity 134, member B	FAM134B	-0.85299911	-1.80625	0.035341012

209210_s_at	fermitin family homolog 2 (Drosophila)	FERMT2	-0.85454842	-1.80819	0.020336612
215726_s_at	cytochrome b5 type A (microsomal)	CYB5A	-0.85704006	-1.81132	0.030412658
218953_s_at	prenylcysteine oxidase 1 like	PCYOX1L	-0.85772229	-1.81218	0.011833306
206833_s_at	acylphosphatase 2, muscle type	ACYP2	-0.8585975	-1.81327	0.00740596
209155_s_at	5'-nucleotidase, cytosolic II	NT5C2	-0.86052889	-1.8157	0.018372499
210609_s_at	tumor protein p53 inducible protein 3	TP53I3	-0.87365046	-1.83229	0.044187722
209885_at	ras homolog gene family, member D	RHOD	-0.88119485	-1.8419	0.02784478
213501_at	acyl-Coenzyme A oxidase 1, palmitoyl	ACOX1	-0.88129622	-1.84203	0.005627889
204076_at	ectonucleoside triphosphate diphosphohydrolase 4	ENTPD4	-0.88165913	-1.84249	0.003930777
208579_x_at	H2B histone family, member S	H2BFS	-0.88196031	-1.84288	0.001616605
205807_s_at	tuftelin 1	TUFT1	-0.88262984	-1.84373	0.006334784
221679_s_at	abhydrolase domain containing 6	ABHD6	-0.89026256	-1.85351	0.001616605
209209_s_at	fermitin family homolog 2 (Drosophila)	FERMT2	-0.89075407	-1.85414	0.013348314
210117_at	sperm associated antigen 1	SPAG1	-0.89823687	-1.86379	0.015971168
209836_x_at	bolA homolog 2 (E. coli) /// bolA homolog 2B (E. coli)	BOLA2 ///	-0.89905396	-1.86484	0.006341619
206115_at	early growth response 3	EGR3	-0.89996941	-1.86603	0.004868451
214212_x_at	fermitin family homolog 2 (Drosophila)	FERMT2	-0.90672272	-1.87478	0.0046331
212111_at	syntaxin 12	STX12	-0.90819985	-1.8767	0.006921301
209083_at	coronin, actin binding protein, 1A	CORO1A	-0.91414148	-1.88445	0.007083702
203167_at	TIMP metallopeptidase inhibitor 2	TIMP2	-0.91591583	-1.88677	0.010853986
209806_at	histone cluster 1, H2bk	HIST1H2BK	-0.92039183	-1.89263	0.011512478
216074_x_at	WW and C2 domain containing 1	WWC1	-0.92312744	-1.89622	0.011534663
204544_at	Hermansky-Pudlak syndrome 5	HPS5	-0.92735812	-1.90179	0.005627889
219628_at	zinc finger, matrin type 3	ZMAT3	-0.92737794	-1.90182	0.014129109
204259_at	matrix metallopeptidase 7 (matrilysin, uterine)	MMP7	-0.92763926	-1.90216	0.006949425
202708_s_at	histone cluster 2, H2be	HIST2H2BE	-0.92772625	-1.90228	0.045867156
219403_s_at	heparanase	HPSE	-0.93099157	-1.90659	0.009110746
207826_s_at	inhibitor of DNA binding 3, dominant negative helix-loop-helix protein	ID3	-0.9345611	-1.91131	0.03555525

204720_s_at	DnaJ (Hsp40) homolog, subfamily C, member 6	DNAJC6	-0.95360941	-1.93671	0.012825789
212724_at	Rho family GTPase 3	RND3	-0.95391827	-1.93713	0.005627889
203870_at	ubiquitin specific peptidase 46	USP46	-0.95801787	-1.94264	0.005627889
41047_at	chromosome 9 open reading frame 16	C9orf16	-0.95974469	-1.94497	0.012228762
202659_at	proteasome (prosome, macropain) subunit, beta type, 10	PSMB10	-0.96551134	-1.95276	0.013881707
210397_at	defensin, beta 1	DEFB1	-0.96736044	-1.95526	0.041052549
207469_s_at	pirin (iron-binding nuclear protein)	PIR	-0.97378175	-1.96398	0.028846728
210986_s_at	tropomyosin 1 (alpha)	TPM1	-0.97662063	-1.96785	0.008601198
208737_at	ATPase, H+ transporting, lysosomal 13kDa, V1 subunit G1	ATP6V1G1	-0.97888011	-1.97093	0.006293525
212112_s_at	syntaxin 12	STX12	-0.98102068	-1.97386	0.009110746
206094_x_at		UGT1A1 //,	-0.98327707	-1.97695	0.007257672
214639_s_at	homeobox A1	HOXA1	-0.99411563	-1.99186	0.001543625
210540_s_at	UDP-Gal:betaGlcNAc beta 1,4-galactosyltransferase, polypeptide 4	B4GALT4	-1.00065451	-2.00091	0.034142802
212445_s_at	neural precursor cell expressed, developmentally down-regulated 4-like	NEDD4L	-1.00114232	-2.00158	0.022760006
218109_s_at	major facilitator superfamily domain containing 1	MFSD1	-1.03468417	-2.04867	0.017238997
206117_at	tropomyosin 1 (alpha)	TPM1	-1.04303878	-2.06056	0.01254069
211548_s_at	hydroxyprostaglandin dehydrogenase 15-(NAD)	HPGD	-1.05386965	-2.07609	0.030133423
210987_x_at	tropomyosin 1 (alpha)	TPM1	-1.05728705	-2.08101	0.008601198
201289_at	cysteine-rich, angiogenic inducer, 61	CYR61	-1.06386645	-2.09053	0.037146313
221484_at	UDP-Gal:betaGlcNAc beta 1,4-galactosyltransferase, polypeptide 5	B4GALT5	-1.06398638	-2.0907	0.026683706
219858_s_at	FLJ20160 protein	FLJ20160	-1.06584641	-2.0934	0.030553407
203925_at	glutamate-cysteine ligase, modifier subunit	GCLM	-1.06898817	-2.09796	0.005533101
219397_at	coenzyme Q10 homolog B (S. cerevisiae)	COQ10B	-1.07493195	-2.10662	0.006334784
201058_s_at	myosin, light chain 9, regulatory	MYL9	-1.088787	-2.12695	0.022219699
219014_at	placenta-specific 8	PLAC8	-1.09942948	-2.1427	0.022346379
213085_s_at	WW and C2 domain containing 1	WWC1	-1.10094711	-2.14495	0.009825362

204480_s_at	chromosome 9 open reading frame 16	C9orf16	-1.10207216	-2.14663	0.003901445
220001_at	peptidyl arginine deiminase, type IV	PADI4	-1.10607101	-2.15259	0.006341619
206197_at	non-metastatic cells 5, protein expressed in (nucleoside-diphosphate kinase)	NME5	-1.10714739	-2.15419	0.003101146
218162_at	olfactomedin-like 3	OLFML3	-1.10992217	-2.15834	0.004895765
202976_s_at	Rho-related BTB domain containing 3	RHOBTB3	-1.1111758	-2.16022	0.045289649
219995_s_at	zinc finger protein 750	ZNF750	-1.11493714	-2.16586	0.016486948
213680_at	keratin 6B	KRT6B	-1.12665012	-2.18351	0.005627889
209398_at	histone cluster 1, H1c	HIST1H1C	-1.14265898	-2.20788	0.004895765
206116_s_at	tropomyosin 1 (alpha)	TPM1	-1.1535387	-2.22459	0.003901445
202545_at	protein kinase C, delta	PRKCD	-1.16436395	-2.24134	0.013677352
203914_x_at	hydroxyprostaglandin dehydrogenase 15-(NAD)	HPGD	-1.1702437	-2.2505	0.047363623
212640_at	protein tyrosine phosphatase-like (proline instead of catalytic arginine), member b	PTPLB	-1.18069463	-2.26686	0.046159827
201266_at	thioredoxin reductase 1	TXNRD1	-1.18863455	-2.27937	0.00887733
217551_at	similar to olfactory receptor, family 7, subfamily A, member 17	LOC44145[illegible]	-1.18909008	-2.28009	0.003901445
218280_x_at	histone cluster 2, H2aa3 /// histone cluster 2, H2aa4	HIST2H2A[illegible]	-1.20433753	-2.30431	0.007257672
205623_at	aldehyde dehydrogenase 3 family, memberA1	ALDH3A1	-1.20497321	-2.30533	0.015796973
201468_s_at	NAD(P)H dehydrogenase, quinone 1	NQO1	-1.22404496	-2.33601	0.010853986
202975_s_at	Rho-related BTB domain containing 3	RHOBTB3	-1.24385788	-2.36831	0.014546584
210633_x_at	keratin 10 (epidermolytic hyperkeratosis; keratosis palmaris et plantaris)	KRT10	-1.24466881	-2.36964	0.004868451
212669_at	calcium/calmodulin-dependent protein kinase (CaM kinase) II gamma	CAMK2G	-1.24815124	-2.37537	0.015225077
214290_s_at	histone cluster 2, H2aa3 /// histone cluster 2, H2aa4	HIST2H2A[illegible]	-1.25085744	-2.37983	0.018190072
211597_s_at	HOP homeobox	HOPX	-1.2511356	-2.38029	0.035362626
207126_x_at		UGT1A1 //[illegible]	-1.29133003	-2.44754	0.003901445
213287_s_at	keratin 10 (epidermolytic hyperkeratosis; keratosis palmaris et plantaris)	KRT10	-1.29892663	-2.46046	0.006293525

205074_at	solute carrier family 22 (organic cation/carnitine transporter), member 5	SLC22A5	-1.29999165	-2.46227	0.008191654
204532_x_at		UGT1A1 //,	-1.30194215	-2.46561	0.005627889
201939_at	polo-like kinase 2 (Drosophila)	PLK2	-1.3048292	-2.47054	0.016824371
218231_at	N-acetylglucosamine kinase	NAGK	-1.31415632	-2.48657	0.003901445
221215_s_at	receptor-interacting serine-threonine kinase 4	RIPK4	-1.35927642	-2.56556	0.047324525
221485_at	UDP-Gal:betaGlcNAc beta 1,4-galactosyltransferase, polypeptide 5	B4GALT5	-1.39040879	-2.62153	0.020085667
206600_s_at	solute carrier family 16, member 5 (monocarboxylic acid transporter 6) /// similar to MCT	LOC10013:	-1.39268865	-2.62568	0.003572379
202786_at	serine threonine kinase 39 (STE20/SPS1 homolog, yeast)	STK39	-1.39449198	-2.62896	0.006054254
208539_x_at	small proline-rich protein 2B	SPRR2B	-1.41153065	-2.66019	0.035726339
203962_s_at	nebulette	NEBL	-1.42063854	-2.67704	0.048325018
203961_at	nebulette	NEBL	-1.4343218	-2.70255	0.028368833
207023_x_at	keratin 10 (epidermolytic hyperkeratosis; keratosis palmaris et plantaris)	KRT10	-1.444271	-2.72125	0.006334784
204602_at	dickkopf homolog 1 (Xenopus laevis)	DKK1	-1.45021124	-2.73248	0.005879063
207147_at	distal-less homeobox 2	DLX2	-1.45941253	-2.74996	0.03244829
208596_s_at		UGT1A1 //,	-1.4847415	-2.79867	0.008103638
208711_s_at	cyclin D1	CCND1	-1.48845248	-2.80588	0.003901445
213590_at	solute carrier family 16, member 5 (monocarboxylic acid transporter 6) /// similar to MCT	LOC10013:	-1.49136409	-2.81155	0.001421337
203002_at	angiomotin like 2	AMOTL2	-1.50015332	-2.82873	0.004838106
215125_s_at		UGT1A1 //,	-1.50816041	-2.84447	0.005943342
212757_s_at	calcium/calmodulin-dependent protein kinase (CaM kinase) II gamma	CAMK2G	-1.51225669	-2.85256	0.012635523
219344_at	solute carrier family 29 (nucleoside transporters), member 3	SLC29A3	-1.5171564	-2.86226	0.003901445
201743_at	CD14 molecule	CD14	-1.53291369	-2.8937	0.003901445
218729_at	latexin	LXN	-1.54795291	-2.92402	0.015434646
209791_at	peptidyl arginine deiminase, type II	PADI2	-1.57640184	-2.98225	0.005627889
208651_x_at	CD24 molecule	CD24	-1.64007177	-3.11681	0.037399893
216379_x_at	CD24 molecule	CD24	-1.64374643	-3.12476	0.020290376

209771_x_at	CD24 molecule	CD24	-1.65086903	-3.14023	0.019186611
205759_s_at	sulfotransferase family, cytosolic, 2B, member 1	SULT2B1	-1.67309381	-3.18898	0.003930777
266_s_at	CD24 molecule	CD24	-1.68268061	-3.21024	0.027725725
208650_s_at	CD24 molecule	CD24	-1.72700286	-3.31039	0.022122228
219934_s_at	sulfotransferase family 1E, estrogen-preferring, member 1	SULT1E1	-1.8038756	-3.49157	0.001277571
201565_s_at	inhibitor of DNA binding 2, dominant negative helix-loop-helix protein	ID2	-1.81546541	-3.51973	0.035341012
214599_at	involucrin	IVL	-1.82087945	-3.53296	0.004868451
210096_at	cytochrome P450, family 4, subfamily B, polypeptide 1	CYP4B1	-1.86813986	-3.65062	0.026995831
206488_s_at	CD36 molecule (thrombospondin receptor)	CD36	-1.89815377	-3.72736	0.03149693
208712_at	cyclin D1	CCND1	-1.90322488	-3.74048	0.003672628
204130_at	hydroxysteroid (11-beta) dehydrogenase 2	HSD11B2	-2.03911021	-4.10992	0.018188987
202435_s_at	cytochrome P450, family 1, subfamily B, polypeptide 1	CYP1B1	-2.05457216	-4.1542	0.016108825
218963_s_at	keratin 23 (histone deacetylase inducible)	KRT23	-2.07004603	-4.199	0.003901445
219722_s_at	glycerophosphodiester phosphodiesterase domain containing 3	GDPD3	-2.08062197	-4.2299	0.00887733
209772_s_at	CD24 molecule	CD24	-2.11400124	-4.3289	0.023579078
202437_s_at	cytochrome P450, family 1, subfamily B, polypeptide 1	CYP1B1	-2.1295861	-4.37592	0.009110746
214456_x_at	serum amyloid A1 /// serum amyloid A2	SAA1 /// SA	-2.17926596	-4.52923	0.016278635
208607_s_at	serum amyloid A1 /// serum amyloid A2	SAA1 /// SA	-2.25229174	-4.76439	0.019822177
219529_at	chloride intracellular channel 3	CLIC3	-2.39940208	-5.27584	0.016698837
203180_at	aldehyde dehydrogenase 1 family, member A3	ALDH1A3	-2.44128472	-5.43125	0.00245319
202436_s_at	cytochrome P450, family 1, subfamily B, polypeptide 1	CYP1B1	-2.47847581	-5.57308	0.006334784
206884_s_at	sciellin	SCEL	-2.50815335	-5.68891	0.029594023
205319_at	prostate stem cell antigen	PSCA	-2.729491	-6.63222	0.009825362
204351_at	S100 calcium binding protein P	S100P	-2.87697166	-7.34607	0.012973447
214549_x_at	small proline-rich protein 1A	SPRR1A	-3.05933374	-8.33588	0.004926805
206392_s_at	retinoic acid receptor responder (tazarotene induced) 1	RARRES1	-3.15718048	-8.92085	0.027885596

205064_at	small proline-rich protein 1B (cornifin)	SPRR1B	-3.19705171	-9.17083	0.006765098
221872_at	retinoic acid receptor responder (tazarotene induced) 1	RARRES1	-3.35181061	-10.2093	0.026584734
213796_at	small proline-rich protein 1A	SPRR1A	-4.11411186	-17.3169	0.003930777
