# Supplementary material for: Tumor Suppressor Function of Syk in Human MCF10A In Vitro and Normal Mouse Mammary Epithelium In Vivo
Source: PLoS One. 2009 Oct 15;4(10):e7445. doi: 10.1371/journal.pone.0007445 (PMC2759536; doi:10.1371/journal.pone.0007445)
Supplement: Table S2 — List of gene probe sets from merged networks 1, 2, and 10. Gene probe sets that were differentially regulated following Syk knockdown of MCF10A cells cultured on collagen (Supplementary Table 1) were submitted to Ingenuity for analysis. Networks 1, 2 and 10 identified by analysis with Ingenuity were merged and the gene probes listed here. The majority of entries were represented by gene probe sets whose expression was differentially regulated following Syk knockdown of MCF10A cells cultured on collagen as indicated. (0.01 MB PDF) [file pone.0007445.s003.pdf]

Differentially expressed on collagen
Networks 1, 2, 10

Â© 2000-2009 Ingenuity Systems, Inc. All rights reserved.

Symbol	Entrez Gene Name	Affymetrix	Fold Change	Location	Family
ALDH1A3	aldehyde dehydrogenase 1 family, member A3	203180_at	-5.43125168	Cytoplasm	enzyme
CD24	CD24 molecule	209772_s_at	-4.32890229	Plasma Membrane	other
HSD11B2	hydroxysteroid (11-beta) dehydrogenase 2	204130_at	-4.1099197	Cytoplasm	enzyme
AMOTL2	angiomotin like 2	203002_at	-2.82872773	Plasma Membrane	other
DLX2	distal-less homeobox 2	207147_at	-2.74996362	Nucleus	transcription regulator
RIPK4	receptor-interacting serine-threonine kinase 4	221215_s_at	-2.56556472	Nucleus	kinase
PLK2	polo-like kinase 2 (Drosophila)	201939_at	-2.47054478	Nucleus	kinase
ALDH3A1	aldehyde dehydrogenase 3 family, memberA1	205623_at	-2.30532989	Cytoplasm	enzyme
TXNRD1	thioredoxin reductase 1	201266_at	-2.27936909	Cytoplasm	enzyme
CYR61	cysteine-rich, angiogenic inducer, 61	201289_at	-2.09052668	Extracellular Space	other
FERMT2	fermitin family homolog 2 (Drosophila)	214212_x_at	-1.87478183	Cytoplasm	other
RIT1	Ras-like without CAAX 1	209882_at	-1.77586775	Plasma Membrane	enzyme
MSMB	microseminoprotein, beta-	210297_s_at	-1.72177723	Extracellular Space	other
PI3	peptidase inhibitor 3, skin-derived	203691_at	-1.68135697	Extracellular Space	other
ALDH3B1	aldehyde dehydrogenase 3 family, member B1	205640_at	-1.67376791	Cytoplasm	enzyme
TRAK2	trafficking protein, kinesin binding 2	202124_s_at	-1.6367793	Plasma Membrane	transporter
BAMBI	BMP and activin membrane-bound inhibitor homolog (Xenopus laevis)	203304_at	-1.55781893	Plasma Membrane	other
C20ORF24	chromosome 20 open reading frame 24	217835_x_at	-1.55173225	Cytoplasm	other
ALDH5A1	aldehyde dehydrogenase 5 family, member A1	203608_at	-1.55120625	Cytoplasm	enzyme
SLC11A2	solute carrier family 11 (proton-coupled divalent metal ion transporters), member 2	203123_s_at	-1.53171003	Plasma Membrane	transporter
CDC42EP4	CDC42 effector protein (Rho GTPase binding) 4	214721_x_at	-1.51582586	Cytoplasm	other
BRD2	bromodomain containing 2	214911_s_at	-1.4531853	Nucleus	kinase
ERLIN2	ER lipid raft associated 2	221542_s_at	-1.44388941	Plasma Membrane	other
BUD31 (includes EG:8896)	BUD31 homolog (S. cerevisiae)	205690_s_at	-1.44020721	Nucleus	transcription regulator
KPNA6	karyopherin alpha 6 (importin alpha 7)	212101_at	-1.4223162	Nucleus	transporter
EIF2B3	eukaryotic translation initiation factor 2B, subunit 3 gamma, 58kDa	218488_at	-1.40234484	Cytoplasm	translation regulator

ATXN10	ataxin 10	208832_at	-1.39633836	Cytoplasm	other
PHF15	PHD finger protein 15	212660_at	-1.39090664	Unknown	other
EIF2B1	eukaryotic translation initiation factor 2B, subunit 1 alpha, 26kDa	201632_at	-1.36476394	Cytoplasm	translation regulator
ZNF259	zinc finger protein 259	217185_s_at	-1.34385725	Nucleus	other
FSTL3	follistatin-like 3 (secreted glycoprotein)	203592_s_at	-1.31873589	Extracellular Space	other
LTBP3	latent transforming growth factor beta binding protein 3	219922_s_at	-1.30575275	Extracellular Space	other
VPS33A	vacuolar protein sorting 33 homolog A (S. cerevisiae)	204590_x_at	-1.29538652	Cytoplasm	transporter
TERF2IP	telomeric repeat binding factor 2, interacting protein	201174_s_at	-1.29187749	Nucleus	other
PIP5K3	phosphatidylinositol-3-phosphate/phosphatidylinositol 5-kinase, type III	213111_at	-1.29162373	Cytoplasm	kinase
SLC7A1	solute carrier family 7 (cationic amino acid transporter, y+ system), member 1	212290_at	-1.27252637	Plasma Membrane	transporter
WFS1	Wolfram syndrome 1 (wolframin)	202908_at	-1.26906358	Cytoplasm	enzyme
TLE4	transducin-like enhancer of split 4 (E(sp1) homolog, Drosophila)	204872_at	-1.26469487	Nucleus	transcription regulator
MPRIP	myosin phosphatase Rho interacting protein	212197_x_at	-1.24962601	Cytoplasm	other
TOLLIP	toll interacting protein	217930_s_at	-1.22691924	Cytoplasm	other
POLR3C	polymerase (RNA) III (DNA directed) polypeptide C (62kD)	210573_s_at	-1.2225995	Nucleus	enzyme
C9ORF3	chromosome 9 open reading frame 3	212848_s_at	-1.21805353	Cytoplasm	peptidase
ANKS1A	ankyrin repeat and sterile alpha motif domain containing 1A	212747_at	-1.19636753	Cytoplasm	other
RAPGEF1	Rap guanine nucleotide exchange factor (GEF) 1	204543_at	1.233084894	Cytoplasm	other
DMBT1	deleted in malignant brain tumors 1	208250_s_at	1.238555327	Plasma Membrane	transmembrane receptor
SLC16A3	solute carrier family 16, member 3 (monocarboxylic acid transporter 4)	217691_x_at	1.241242254	Plasma Membrane	transporter
KIAA1199	KIAA1199	212942_s_at	1.263300425	Unknown	other
NAT6	N-acetyltransferase 6 (GCN5-related)	210874_s_at	1.265713615	Cytoplasm	other
IGFBP2	insulin-like growth factor binding protein 2, 36kDa	202718_at	1.278487473	Extracellular Space	other
ADAM8	ADAM metallopeptidase domain 8	205180_s_at	1.288221144	Plasma Membrane	peptidase
ABR	active BCR-related gene	212895_s_at	1.291684857	Unknown	other
ECSIT	ECSIT homolog (Drosophila)	218225_at	1.299803382	Nucleus	transcription regulator

Differentially expressed on collagen
Networks 1, 2, 10

ECOP	EGFR-coamplified and overexpressed protein	208091_s_at	1.322523682	Nucleus	other
SLC37A4	solute carrier family 37 (glucose-6-phosphate transporter), member 4	217289_s_at	1.376175271	Cytoplasm	transporter
ANP32A	acidic (leucine-rich) nuclear phosphoprotein 32 family, member A	201043_s_at	1.409107453	Nucleus	other
RAC3	ras-related C3 botulinum toxin substrate 3 (rho family, small GTP binding protein Rac3)	206103_at	1.44296971	Cytoplasm	enzyme
B3GNT1	UDP-GlcNAc:betaGal beta-1,3-N-acetylglucosaminyltransferase 1	203188_at	1.460637117	Cytoplasm	enzyme
EPHA3	EPH receptor A3	206070_s_at	1.470752251	Plasma Membrane	kinase
TENC1	tensin like C1 domain containing phosphatase (tensin 2)	212494_at	1.485724074	Plasma Membrane	other
STC2	stanniocalcin 2	203439_s_at	1.490425808	Extracellular Space	other
P4HA2	prolyl 4-hydroxylase, alpha polypeptide II	202733_at	1.504593228	Cytoplasm	enzyme
KDELR3	KDEL (Lys-Asp-Glu-Leu) endoplasmic reticulum protein retention receptor 3	204017_at	1.52428352	Cytoplasm	transporter
CIB1	calcium and integrin binding 1 (calmyrin)	201953_at	1.559198164	Nucleus	other
NFIB	nuclear factor I/B	209289_at	1.575191227	Nucleus	transcription regulator
PKP2	plakophilin 2	207717_s_at	1.580201591	Plasma Membrane	other
PPP1R13L	protein phosphatase 1, regulatory (inhibitor) subunit 13 like	218849_s_at	1.591154408	Nucleus	transcription regulator
RLF	rearranged L-myc fusion	204243_at	1.649532526	Nucleus	transcription regulator
PLOD1	procollagen-lysine 1, 2-oxoglutarate 5-dioxygenase 1	200827_at	1.663176552	Cytoplasm	enzyme
ITGB5	integrin, beta 5	201125_s_at	1.688160654	Plasma Membrane	other
TSPAN4	tetraspanin 4	209264_s_at	1.699349028	Plasma Membrane	other
NCAPD2	non-SMC condensin I complex, subunit D2	201774_s_at	1.70549251	Nucleus	other
RRAS	related RAS viral (r-ras) oncogene homolog	212647_at	1.756246341	Cytoplasm	enzyme
SMTN	smoothelin	207390_s_at	1.781277947	Extracellular Space	other
LGALS7	lectin, galactoside-binding, soluble, 7	206400_at	1.792159574	Extracellular Space	other
ADAM12	ADAM metallopeptidase domain 12	202952_s_at	1.845508867	Plasma Membrane	peptidase
EGFR	epidermal growth factor receptor (erythroblastic leukemia viral (v-erb-b) oncogene homolog, avian)	201983_s_at	1.869975921	Plasma Membrane	kinase
AXL	AXL receptor tyrosine kinase	202686_s_at	1.925814869	Plasma Membrane	kinase
IGFBP6	insulin-like growth factor binding protein 6	203851_at	2.036518664	Extracellular Space	other

Differentially expressed on collagen
Networks 1, 2, 10

CARHSP1	calcium regulated heat stable protein 1, 24kDa	218384_at	2.066136852	Cytoplasm	other
ERF	Ets2 repressor factor	203643_at	2.088396999	Nucleus	transcription regulator
TGFB1	transforming growth factor, beta 1	203085_s_at	2.397413656	Extracellular Space	growth factor
SPAG4	sperm associated antigen 4	219888_at	2.578189024	Cytoplasm	other
P4HA1	prolyl 4-hydroxylase, alpha polypeptide I	207543_s_at	2.672517765	Cytoplasm	enzyme
MXI1	MAX interactor 1	202364_at	2.686706013	Nucleus	transcription regulator
PLOD2	procollagen-lysine, 2-oxoglutarate 5-dioxygenase 2	202620_s_at	2.703934291	Cytoplasm	enzyme
IGFBP7	insulin-like growth factor binding protein 7	201163_s_at	3.095274939	Extracellular Space	transporter
CA12	carbonic anhydrase XII	203963_at	3.376692262	Plasma Membrane	enzyme
LOXL2	lysyl oxidase-like 2	202998_s_at	4.341355328	Extracellular Space	enzyme
LOX	lysyl oxidase	215446_s_at	4.682900868	Extracellular Space	enzyme
Aldehyde dehydrogenase (NAD)				Unknown	group
Aldehyde dehydrogenase [NAD(P)]				Unknown	group
ALDH				Unknown	group
Collagen(s)				Unknown	complex
Creatine Kinase				Unknown	group
eIF				Unknown	complex
Igfbp				Unknown	group
Integrin				Unknown	complex
Metalloprotease				Unknown	group
NFkB (complex)				Unknown	complex
Nuclear factor 1				Unknown	group
Rac				Unknown	group
Rap1				Unknown	group
Ras homolog				Unknown	group
Rock				Unknown	group
Sos				Unknown	group
