# Supplementary material for: Tumor Suppressor Function of Syk in Human MCF10A In Vitro and Normal Mouse Mammary Epithelium In Vivo
Source: PLoS One. 2009 Oct 15;4(10):e7445. doi: 10.1371/journal.pone.0007445 (PMC2759536; doi:10.1371/journal.pone.0007445)
Supplement: Table S3 — Invadopodia gene probes that were differentially regulated following Syk knockdown in cells cultured on collagen. 139 gene probes were linked with 63 invadopodia-related proteins (proteins whose role in invadopodia function was determined by the criteria of matrix degradation on gelatin crosslinked films or localization of MT1-MMP at invadopodia [1]). Levels of 17 gene probes were significantly changed at the FDR level of 0.05 as described in Materials and Methods. (0.01 MB PDF) [file pone.0007445.s004.pdf]

Invadopodia genes differentially regulated by Syk on collagen

Symbol	probesetID	logFC.collagen	FC.collagen	adj.p.value.collagen	Name
CORO1A	209083_at	-0.914141478	-1.8844473	0.006585741	\coronin actin binding protein 1A\""
WASL	205809_s_at	-0.509331782	-1.4233908	0.012800036	Wiskott-Aldrich syndrome-like
GRB2	215075_s_at	-0.436135236	-1.3529751	0.011568722	growth factor receptor-bound protein 2
PLAUR	214866_at	-0.377133738	-1.298759	0.025548631	\plasminogen activator urokinase receptor\""
ITGB1	216178_x_at	0.311594469	1.24107858	0.03755208	\integrin beta 1 (fibronectin receptor beta polypeptide antigen CD29 includes MDF2 MSK12)\""
PAK1	209615_s_at	0.31722668	1.24593316	0.011568722	\p21/Cdc42/Rac1-activated kinase 1 (STE20 homolog yeast)\""
MAPK7	207292_s_at	0.33294946	1.25958585	0.03755208	mitogen-activated protein kinase 7
LAMP1	213728_at	0.336918756	1.26305613	0.025548631	lysosomal-associated membrane protein 1
MAPK7	35617_at	0.364197359	1.28716532	0.032093012	mitogen-activated protein kinase 7
WASF1	204165_at	0.619532702	1.53637746	0.011568722	\WAS protein family member 1\""
LAMP1	201551_s_at	0.75074257	1.68265869	0.011568722	lysosomal-associated membrane protein 1
VEGFA	210513_s_at	0.794712981	1.73473222	0.011568722	vascular endothelial growth factor A
VEGFA	212171_x_at	0.833348452	1.78181611	0.012800036	vascular endothelial growth factor A
ADAM12	202952_s_at	0.884018669	1.84550887	0.013125808	ADAM metallopeptidase domain 12 (meltrin alpha)
VEGFA	211527_x_at	0.910792186	1.88007757	0.013022393	vascular endothelial growth factor A
TGFB1	203085_s_at	1.261478857	2.39741366	0.011568722	\transforming growth factor beta 1\""
VEGFA	210512_s_at	1.47723116	2.78413885	0.011568722	vascular endothelial growth factor A
