# Supplementary figures and images for: Tumor Suppressor Function of Syk in Human MCF10A In Vitro and Normal Mouse Mammary Epithelium In Vivo
Source: PLoS One. 2009 Oct 15;4(10):e7445. doi: 10.1371/journal.pone.0007445 (PMC2759536; doi:10.1371/journal.pone.0007445)

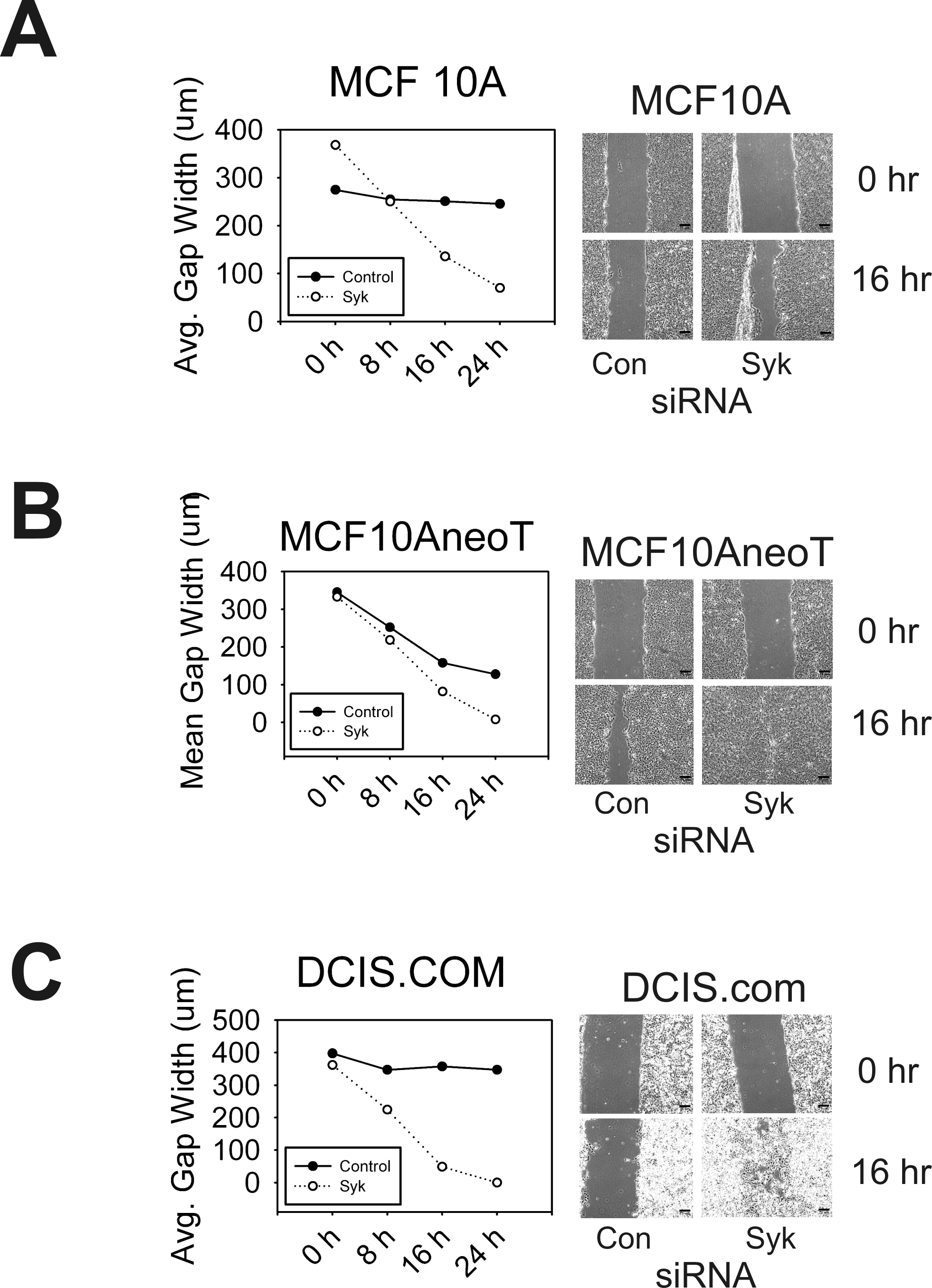

Supplement: Figure S1 — Time course of scrape/wound closure for MCF10A, MCF10AneoT, and DCIS.com human mammary epithelial cells. A scratch wound was made and the degree of closure achieved over 24-hr incubation was imaged by time lapse microscopy and measured using Metamorph Image Analysis software. Graphs and representative images from cell lines MCF10A (A), MCF10AneoT (B), and DCIS.com (C) are shown at 0 hr and 16 hrs. Scale bars correspond to 75 µm in all panels. (1.06 MB TIF) [file pone.0007445.s005.tif]

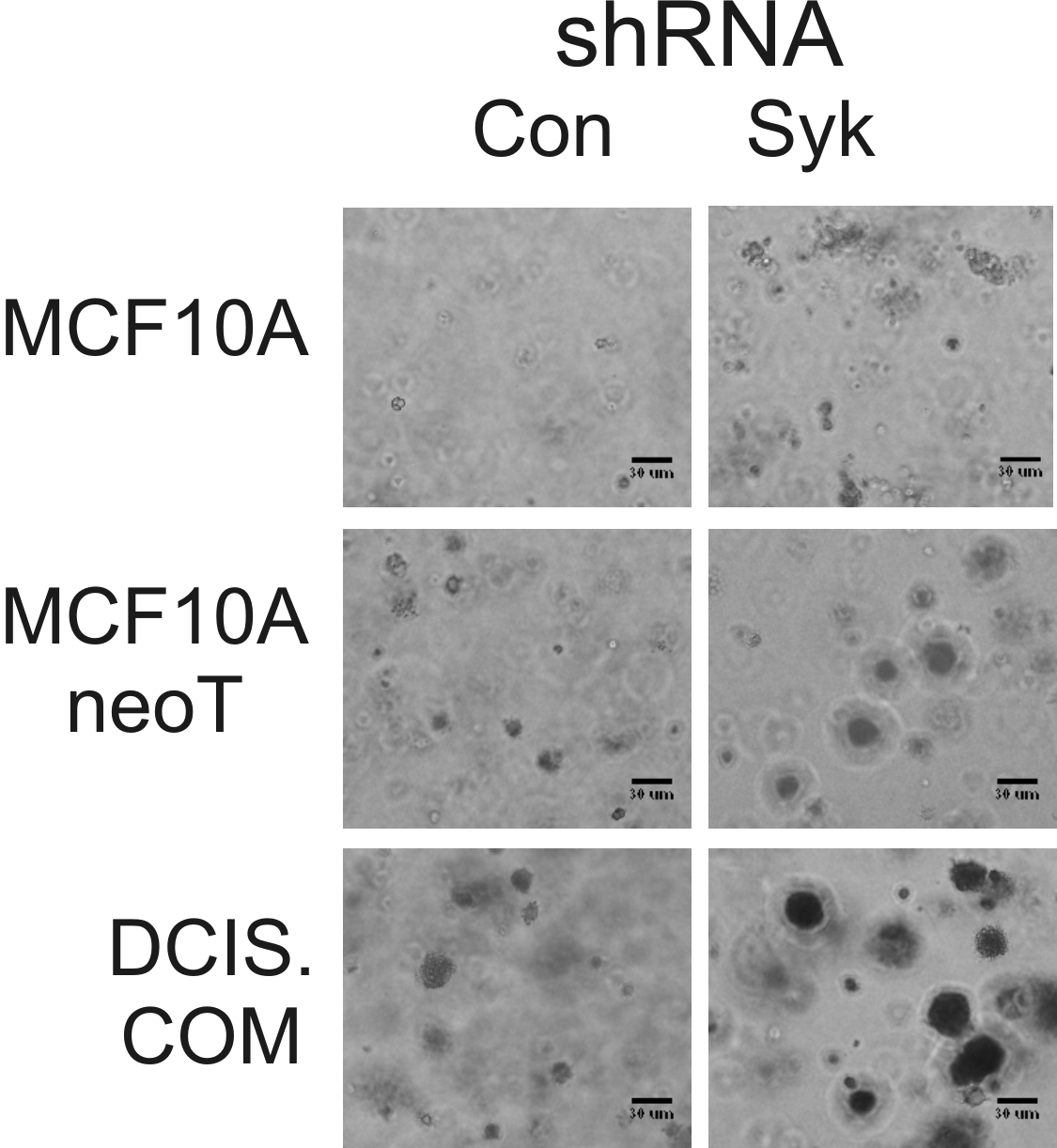

Supplement: Figure S2 — Soft agar growth assays. In soft agar assays, each of the Syk shRNA knockdown cell lines experienced increased colony formation. Representative images are shown. Scale bars are all 30 µm. (0.40 MB TIF) [file pone.0007445.s006.tif]

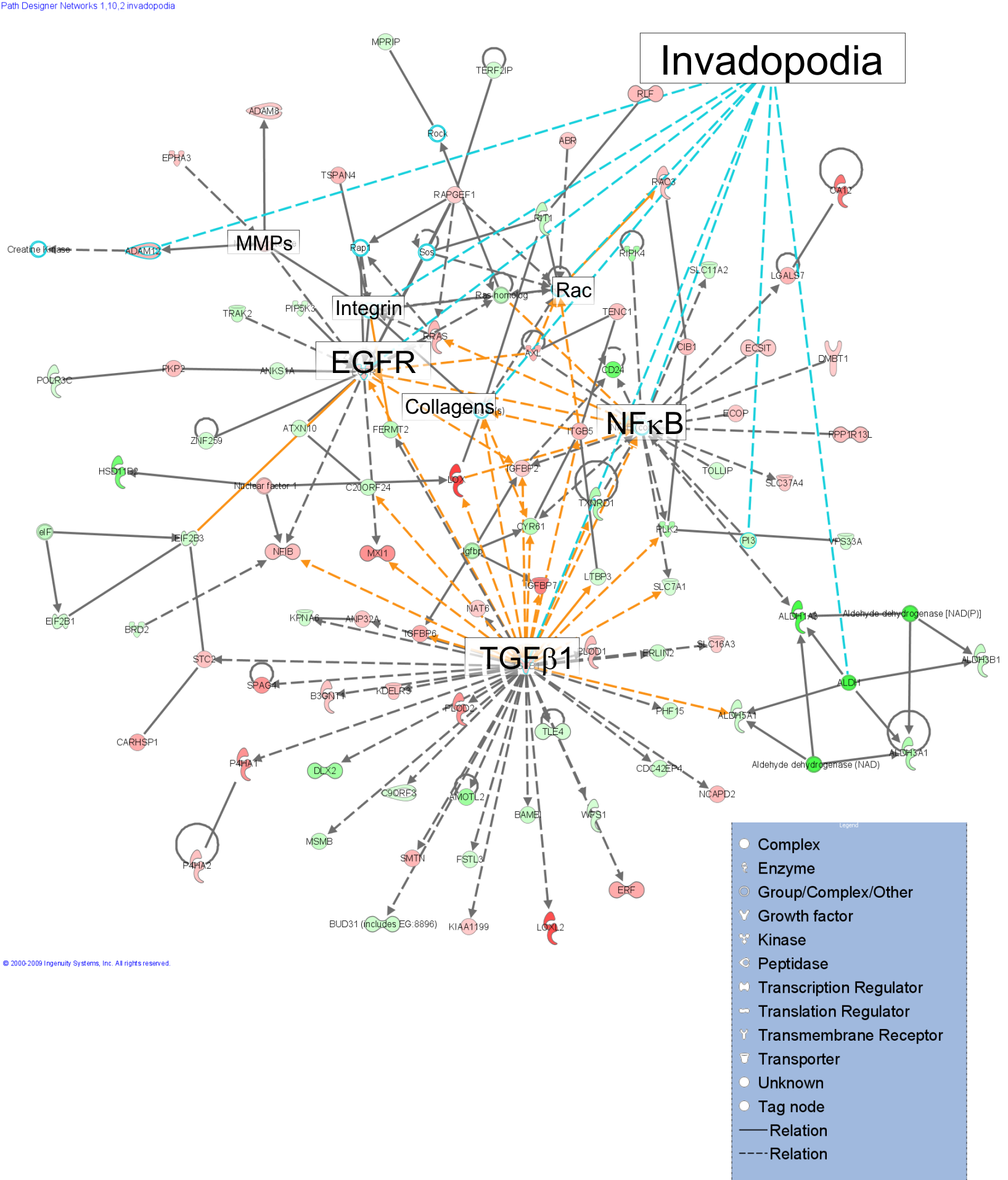

Supplement: Figure S3 — Detailed map of three merged networks. Cells cultured on collagen were analyzed to determine up- or down-regulated gene probes following Syk knockdown. TGFβ1, NFκB, and EGFR constitute major “hubs” of three networks (networks 1, 2 and 10) identified by Ingenuity (Supplementary Table 2). The links between these three networks are shown in yellow. The intersection of the invadopodia network (not shown) with the EGFR-NFκB-TGFβ1 linked networks is indicated by dashed blue lines. Probe sets up-regulated are shown in red, those down-regulated are shown in green. (0.98 MB TIF) [file pone.0007445.s007.tif]

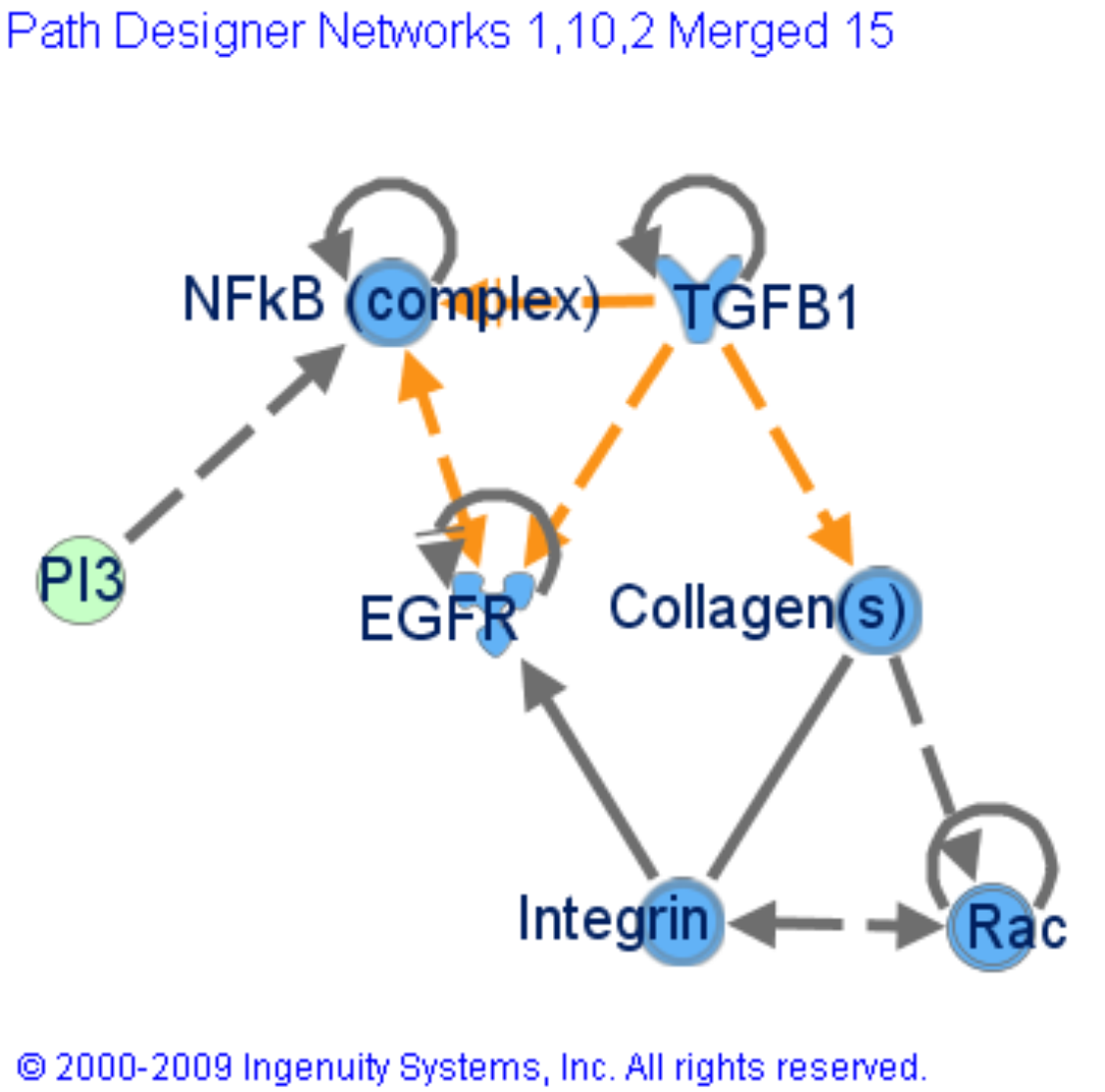

Supplement: Figure S4 — Summary of major nodes in three networks. A summary of the major nodes of interest in networks 1, 2, and 10 are shown, taken from Supplementary Figure 1. Orange lines indicate interactions among the networks. (0.37 MB TIF) [file pone.0007445.s008.tif]

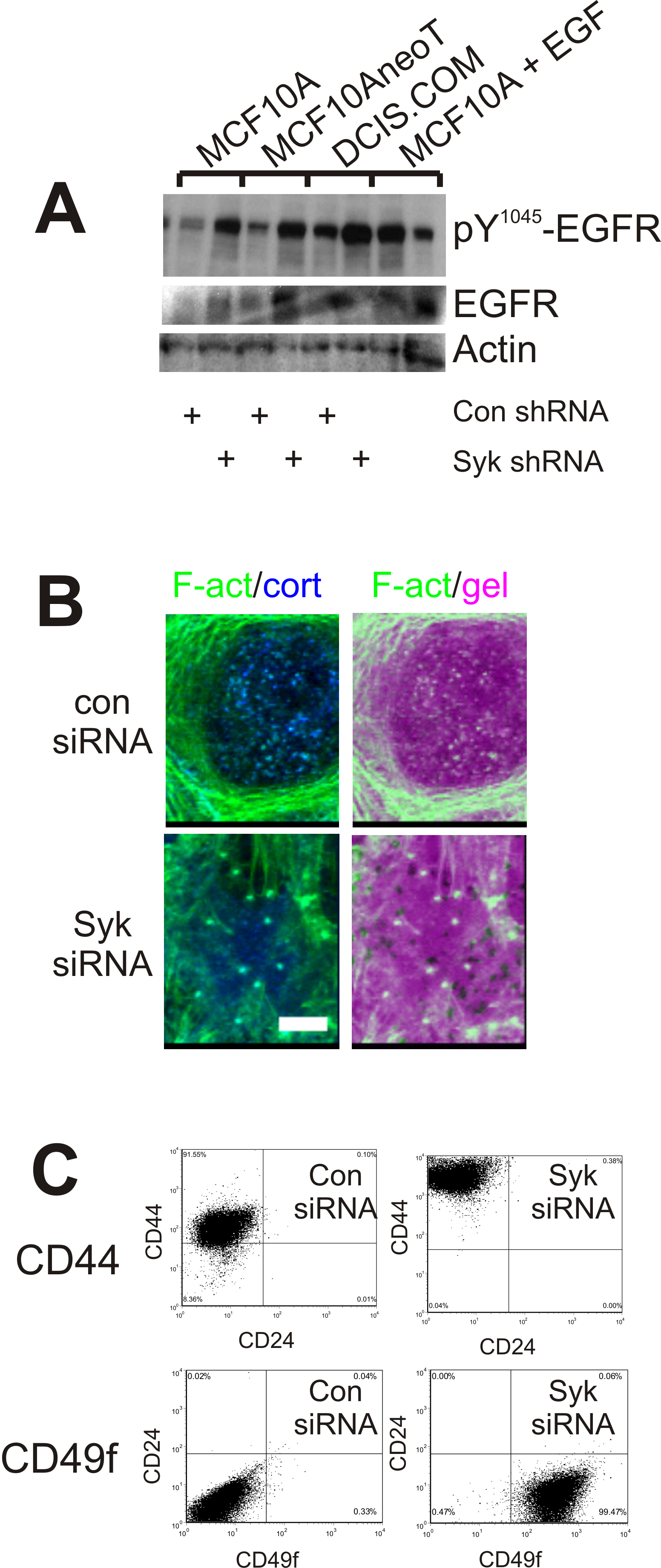

Supplement: Figure S5 — EGFR, invadopodia, and cell surface invadopodia/stem cell markers. (A) Extracts of MCF10A, MCF10AneoT, and DCIS.com transfected with control or Syk siRNA were probed for anti-pY1045-EGFR, and then stripped and re-probed for anti-EGFR and then again for anti-α-actin. Overall, activated EGFR was up-regulated by Syk knockdown. The last two lanes are two different loadings of EGF-treated MCF10A positive control cell lysates. (B) Images from the gelatin-degradation assay for invadopodia from MCF10A cells from three color confocal imaging (phalloidin, green; cortactin, blue; gelatin, magenta). Higher magnification images selected are shown here and indicated in Fig. 4D by the box. Scale bars = 10 µm. (C). Two color flow cytometry for CD44/CD24 (CD44) and CD49f (α6 integrin)/CD24 (CD49f) demonstrates increased cell surface CD44 and CD49f with unchanged or slightly decreased cell surface CD24 following Syk knockdown by siRNA and culture of MCF10A cells on plastic. (2.17 MB TIF) [file pone.0007445.s009.tif]

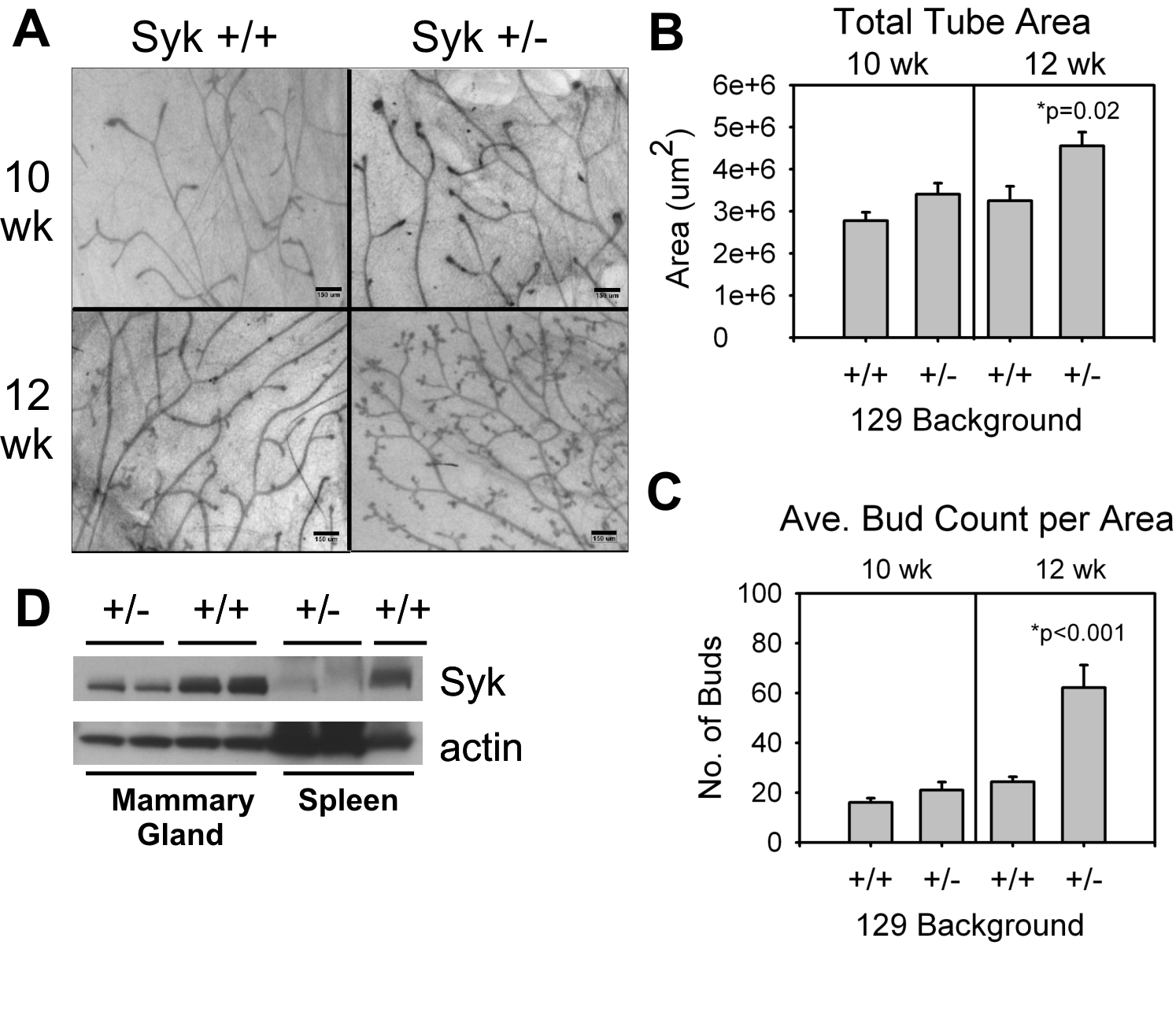

Supplement: Figure S6 — Enhanced branching morphogenesis in mammary glands of Syk +/− mice (129 background). (A) Mammary gland branching and end buds are more prominent in Syk +/− heterozygote. Scale bars correspond to 150 µm in all panels. The average number of total tube area (B) and buds (C) were counted in mammary glands from Syk +/+ wild type and Syk +/− heterozygous mice. (D) Syk protein is decreased in Western blots of mammary glands extracts (minus the mammary gland lymph node) and spleen from 12-week virgin Syk +/− heterozygous compared with Syk +/+ wild type females. Spleen extracts were used as positive control. (0.83 MB TIF) [file pone.0007445.s010.tif]

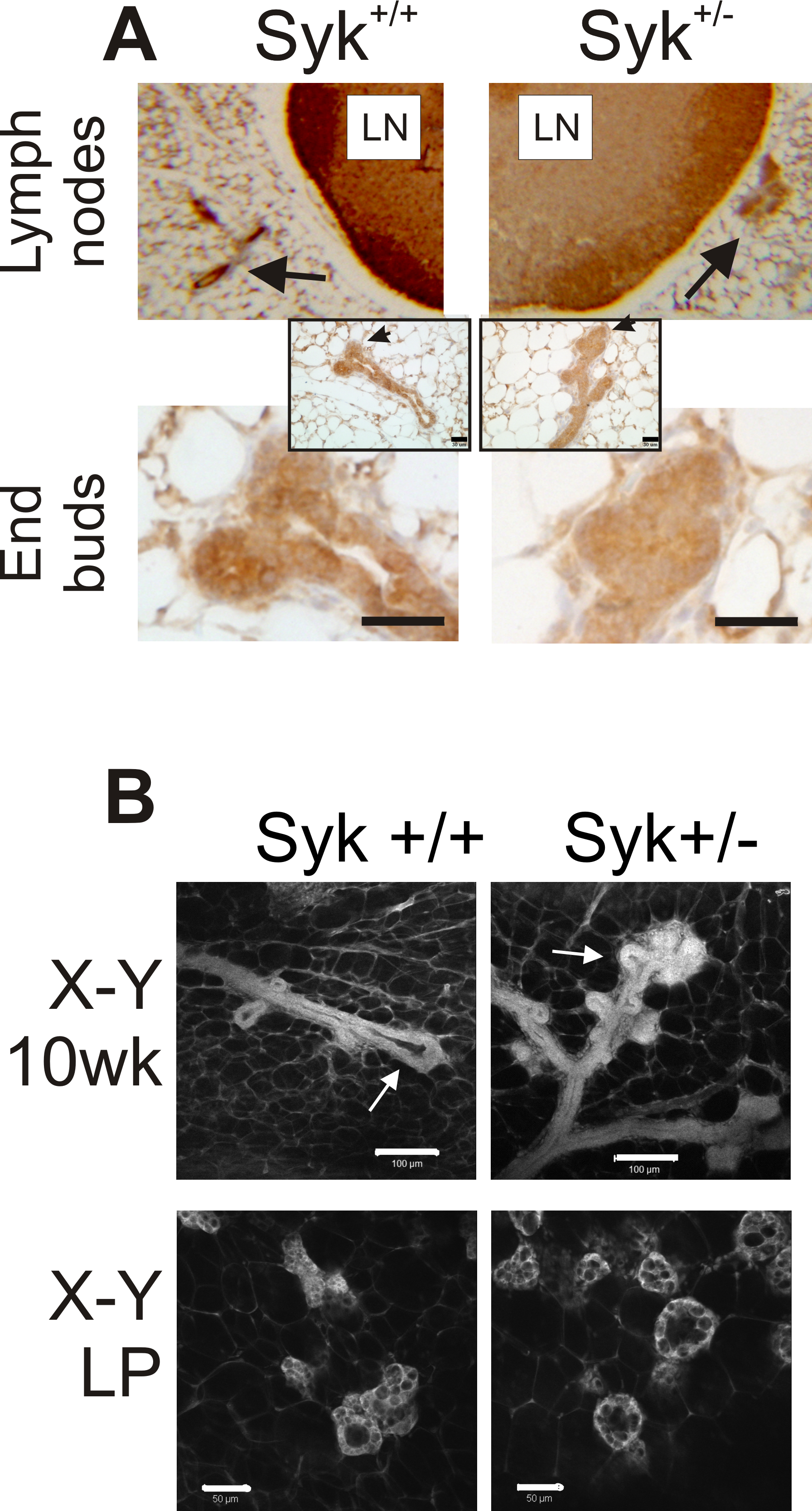

Supplement: Figure S7 — Enhanced branching morphogenesis in mammary glands of Syk +/− mice. (A) Immunohistochemistry staining using N-19 rabbit polyclonal anti-Syk antibody was performed on paraffin sections of mammary glands from 10 week females. Decreased expression of Syk protein is observed in mammary epithelial cells in Syk +/− heterozygous versus Syk +/+ wild type mice. Mammary gland lymph nodes (LN) serve as internal controls where Syk is expressed in different subpopulations of lymphocytes. Adjacent ducts are indicated by arrows. Higher magnification views of end buds are shown below, illustrating positively stained luminal epithelium. Images were taken using identical microscope settings for comparison. In the lower panel, scale bars correspond to 10 µm and in their higher magnification insets to 30 µm. (B) Mammary glands from Syk +/+ wild type and Syk +/− heterozygote knockout mice of 10 week (10 wk) virgin females and late pregnancy (LP) females were dissected out and stained with carmine red in whole mounts. Mammary gland branching and end buds are more prominent in Syk +/− heterozygote as illustrated in these micrographs. Scale bars correspond to 150 µm in all panels. (4.42 MB TIF) [file pone.0007445.s011.tif]
